# DiscERN: an automated genome mining tool for the discovery of evolutionarily related natural products

**DOI:** 10.1038/s41467-026-75491-x

**Published:** 2026-07-14

**Authors:** Jeremy G. Owen, Ethan F. Woolly, Hung-En Lai, Victoria H. Woolner, Rory F. Little

**Affiliations:** 1 School of Biological Sciences, Victoria University of Wellington, Wellington, New Zealand; 2 Maurice Wilkins Centre for Molecular Biodiscovery, Wellington, New Zealand; 3 Centre for Biodiscovery, School of Biological Sciences, Victoria University of Wellington, Wellington, New Zealand

**Keywords:** Drug screening, Data mining, Biosynthesis, Cheminformatics, Antibiotics

## Abstract

Targeted genome mining to expand known families of natural products is a powerful strategy for discovering bioactive compounds, yet it remains a significant bioinformatics challenge. While tools exist for de novo biosynthetic gene cluster identification and large-scale unsupervised clustering, dedicated methods for the targeted, hypothesis-driven expansion of user-defined BGC families are lacking. Here, we present DiscERN (Discoverer of Evolutionarily Related Natural products), a user-friendly tool designed to address this gap. DiscERN leverages a multi-modal ensemble method that integrates four complementary algorithms classifying biosynthetic gene clusters based on Pfam content, sequence homology, and predicted product structure. This approach allows users to strategically balance discovery sensitivity with predictive precision to suit diverse research goals. We demonstrate DiscERN’s utility by applying it to a large collection of actinomycete genomes and validating its predictive power through the successful isolation of discomycin A, a new calcium-dependent lipopeptide antibiotic, from a silent biosynthetic gene cluster. DiscERN provides a robust and accessible platform that streamlines the path from genomic data to a prioritised list of candidate biosynthetic gene clusters, effectively bridging the gap between in silico prediction and bioactive compound discovery.

## Abstract (web summary)

Bioactive natural products occur in evolutionarily related families. Here the authors present DiscERN, a genome mining tool that exploits this to guide discovery, as exemplified by isolation of discomycin A, a lipopeptide antibiotic from a silent gene cluster.

## Introduction

The inexorable rise of antimicrobial resistance constitutes a global health crisis, threatening to undermine pillars of modern medicine^1^. In response, there has been a renewed search for new chemical scaffolds with antibacterial activity. Historically, bacterial natural products have been the most prolific source of antibiotics^2^. These complex compounds are the result of millions of years of evolution, fine-tuning them for potent and specific biological activities^3–5^. It is now widely recognized that the true biosynthetic potential of the microbial world remains vastly underexplored, offering a rich, untapped reservoir of chemical diversity for drug discovery and biomedical research^6,7^.

Genomic data have revealed that the capacity to produce natural products is encoded within discrete, co-located sets of genes known as Biosynthetic Gene Clusters (BGCs)^8,9^, where each BGC acts as a molecular assembly line, containing the complete enzymatic “blueprint” for constructing a specific natural product^10^. The advent of high-throughput genome sequencing, coupled with advances in synthetic biology and heterologous expression, have revolutionised our ability to translate these genomic blueprints into chemical reality^11–13^. Strikingly, genome mining studies consistently show that the number of BGCs identified in microbial genomes far exceeds the number of known compounds isolated from those same organisms^6,8^. This vast collection of “silent” or cryptic BGCs represents an opportunity for discovering new molecules, reducing the reliance on bioactivity guided screening, which is stochastic and frequently confounded by the rediscovery of known compounds^2^.

Computational tools are central to unlocking this opportunity. The development of antiSMASH^14^, a widely-used platform for BGC annotation, and its rich informatics ecosystem have enabled researchers to systematically identify and classify BGCs from genomic data^11,15–17^. One of the most fruitful strategies in genome mining is the targeted expansion of known natural product families^18–22^. BGCs responsible for producing a particular class of compounds, such as the glycopeptide antibiotics^23^ or the 16-membered polyketide macrolides^24^, often share a conserved biosynthetic functions and sequence homology. By using known, well-characterised BGCs as a query, researchers can mine genomic datasets for evolutionary relatives that might produce new compounds. This genomics-driven approach has proven remarkably successful, leading to the discovery of novel structural variants with improved properties^25^, distinct biological targets^18,20,22^, or antimicrobials with activity against strains resistant to existing clinically employed relatives^21,22,26^.

While this targeted strategy is powerful, it has been hampered by a lack of dedicated, user-friendly tools. Existing state-of-the-art platforms like are designed for the valuable but distinct task of *unsupervised* clustering. They excel at organizing massive BGC collections into similarity networks, providing a global overview of biosynthetic diversity. However, they are not optimized for a hypothesis-driven workflow where a researcher wishes to ask a specific question: “Given this defined set of BGCs for my family of interest, where can I find more like them?” Consequently, researchers are often forced to develop ad hoc search strategies for each family of interest, a process that typically involves manually scripting disparate tools and lacks efficiency, scalability, and reproducibility.

While this targeted strategy is powerful, it has been hampered by a lack of dedicated tools optimized for multi-reference family expansion. Existing state-of-the-art platforms like BiG-SCAPE^16,27^ and BiG-SLiCE^15,27^ are primarily designed for unsupervised clustering and large-scale network analysis, providing a global overview of biosynthetic diversity. Although targeted mining is possible using single-reference homology searches (e.g., cblaster^28^, CORASON^16^) or by adapting clustering tools like BiG-SCAPE and GATOR-GC^29^ for multi-reference queries, these tools were not specifically designed for this task, and require extensive downstream data-mining to prioritise targets for functional study.

**Fig. 1:**
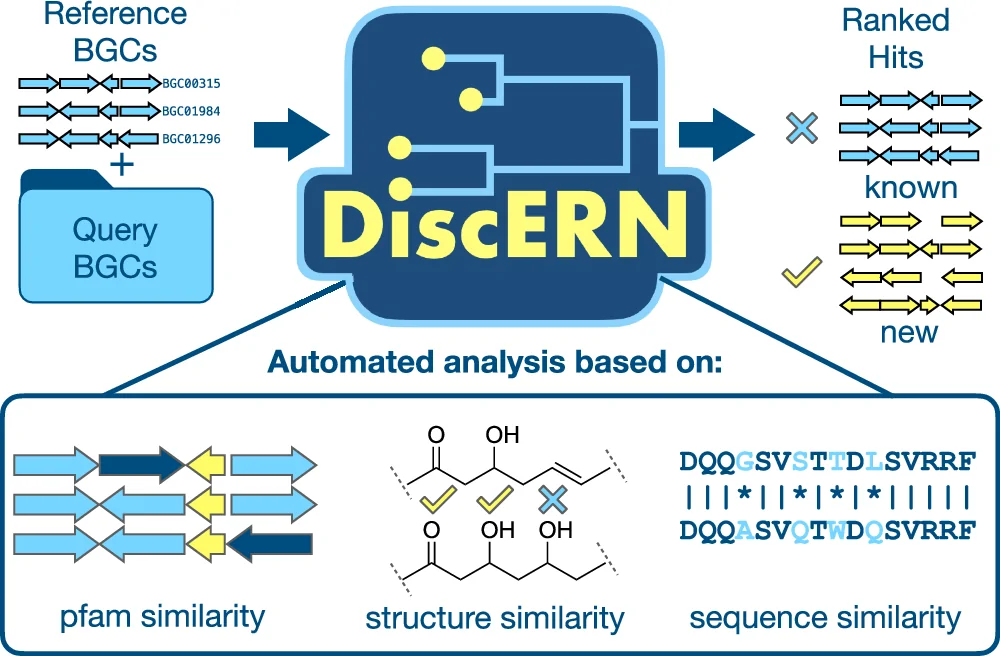
Schematic overview of DiscERN workflow. A user defined family of BGCs specified using MIBiG numbers and directory containing antiSMASH outputs for genomes to be analysed are provided as inputs. DiscERN then applies and integrates an ensemble of algorithmic approaches to identify new family members and provides Intuitive outputs to facilitate prioritisation for downstream functional analysis.

Here, we describe DiscERN (Discoverer of Evolutionarily Related Natural products), an automated and robust method for the targeted expansion of user-defined BGC families (Fig. 1). Recognising that evolutionary relatedness can be captured through different but complementary features^3–5,30^, we built DiscERN around an ensemble of four distinct algorithms. These approaches classify BGCs based on three principles: (i) overall Pfam domain content, providing an architectural fingerprint; (ii) direct protein sequence similarity; and (iii) predicted structure of the final small-molecule product, offering a functional chemical perspective. By integrating these methods into a flexible and intuitive pipeline, DiscERN provides an efficient workflow for analysing large BGC datasets to expand the diversity of target compound classes. As a demonstration of its utility, we applied our method to a collection of 3,561 actinomycete genomes. This search identified numerous BGCs putatively encoding new members of existing natural product families. We activated one of these using heterologous expression and regulatory gene overexpression, leading to the isolation discomycin A, a new member of the calcium-dependent lipopeptide family from a silent BGC in the genome of *Streptomyces kanamyceticus* ATCC12853.

## Results and discussion

### Development of complementary algorithms for BGC family expansion

To create a robust tool for targeted BGC family expansion, we used four complementary classification methods designed to capture different aspects of evolutionary relatedness. The first of these is the Pfam Vector (pfam-vec) method, which adapts the BGC vectorization approach from BiG-SLiCE^15^, and classifies BGCs based on their overall Pfam domain content. This method converts antiSMASH outputs into numerical vectors representing the presence, abundance, and amino acid sequences of Pfam domains, providing a high-level architectural fingerprint of each BGC. To provide a reference for development and benchmarking, we defined six manually curated antibiotic families from the MIBiG database^17^ (Supplementary Data 1): calcium-dependent lipopeptides^31^, glycopeptides^23^, macrolides^24^, aminoglycosides^32^, rifamycin like polyketides^26,33,34^ and polyene antifungals^22,35^. For each family, we calculated a centroid vector representing the average Pfam domain composition and determined an optimal cosine distance threshold to classify family members against a background of all other BGCs, with model performance assessed using the Matthews Correlation Coefficient (MCC)^36^. The maximum achievable MCC score varied (0.8–1.0), suggesting some families are more easily distinguished by Pfam content than others.

**Fig. 2:**
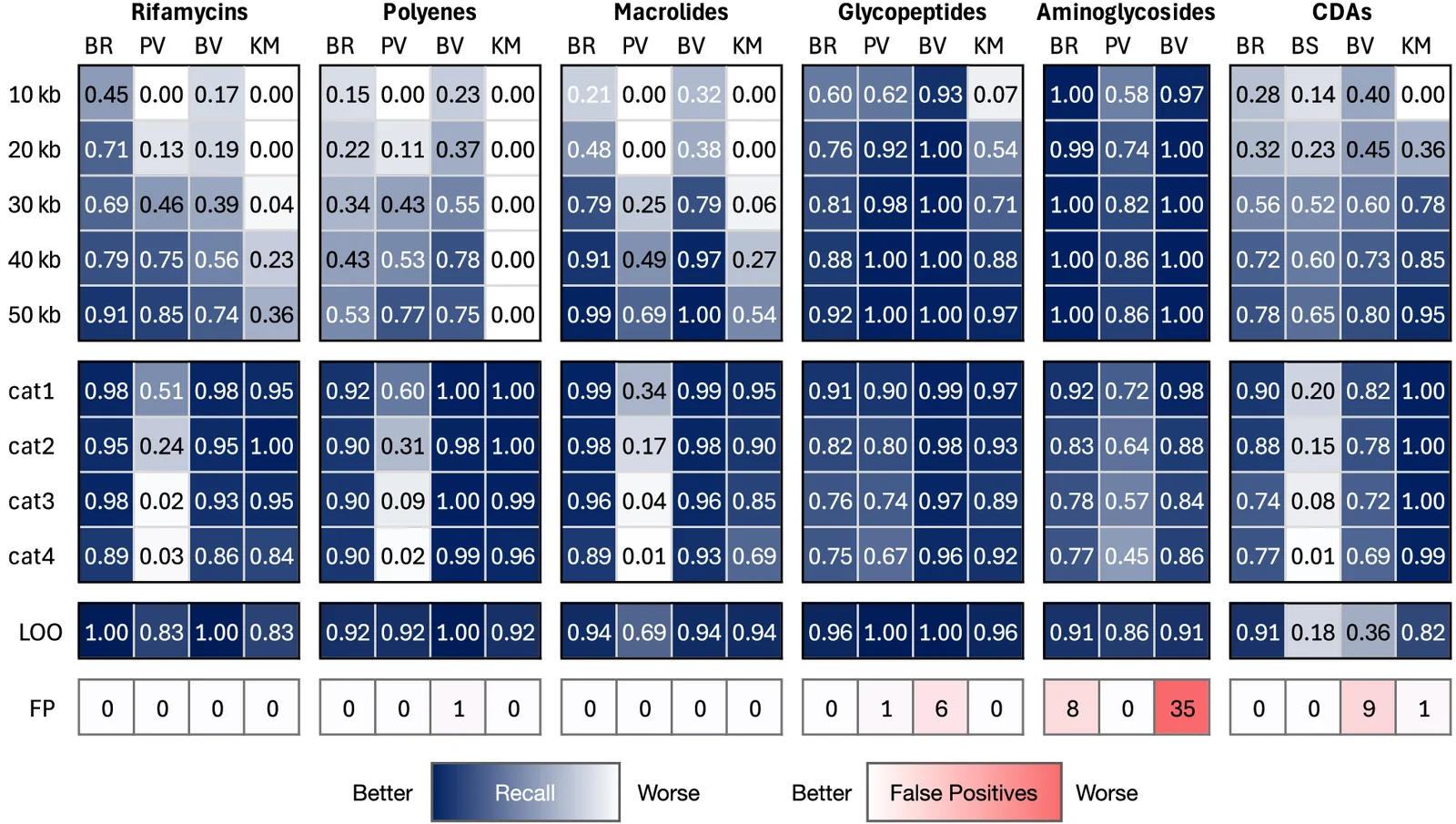
Evaluation of performance for each classification method. Heat maps indicate performance of each method in classification of BGCs belonging to six manually defined antibiotic families. Each panel represents a single antibiotic family. Column labels indicate classification methods: BR blast-rank, BV blast-vec, PV pfam-vec, KM structural k-mer intersection. Row labels indicate assessment metrics: 10–40 kb = Sensitivity of approach when applied to 100 randomly generated BGC sub-fragments of the indicated size. Cat1–4 = Sensitivity of approach when applied to 100 BGCs concatenated with 1–4 additional randomly selected BGCs. LOO = Sensitivity of approach in leave-one-out benchmarking. FP = total false positives among 2613 BGCs extracted from 150 randomly selected actinomycetes genomes. Source data are provided in the Source Data file.

The centroids and optimal cosine distances determined for each BGC family comprise a model that can be used for classification. In our workflow, pfam-vecs are generated for all BGCs to be classified, and their cosine distance from a target BGC family centroid calculated, with BGCs below the previously determined optimal threshold classified as belonging to the family in question. A leave-one-out analysis using this approach to assess out-of-train sensitivity showed variable performance among BGC families. While glycopeptides performed perfectly (recall = 1.0) and others reasonably well (rifamycins, polyenes, aminoglycosides; recall = 0.83–0.92), the macrolide model was average (recall = 0.69) and the CDA model exhibited poor performance (recall = 0.18) (Fig. 2). When challenged with a test set of five recently discovered calcium dependent antibiotics not present in the training data^20,37–40^, the model’s limited generalisability was confirmed, as it correctly identified only three of the five test cases.

### Evaluation of robustness to sequencing artefacts

We further evaluated robustness by simulating fragmented BGCs and concatemers of adjacent BGC, common artifacts arising from fragmented genome assemblies and overlapping BGC boundaries, respectively^41,42^. We generated concatemers by appending a randomly selected BGC (from the antiSMASH 7.0 output of *Actinomycetes* genomes) to each of the MIBiG reference BGCs in our chosen antibiotic families. Fragments were generated by extracting random sequence segments of defined lengths (10, 20, 30, 40, and 50 kb for glycopeptides, lipopeptides, and macrolides; 10 and 20 kb for the smaller aminoglycoside clusters) from the reference BGCs and re-analysing them with antiSMASH.

For each reference BGC, we generated 100 concatemers, as well as 100 fragments of each specified size. These simulated fragmented and concatenated BGCs were then analysed using the models trained for each compound class. The models performed reasonably well on larger fragments but exhibited significantly reduced performance on smaller fragments and concatemers (Fig. 2). This highlighted a need for an alternative scoring method to improve sensitivity in these challenging scenarios.

### Evaluation of specificity

To evaluate the specificity, we compiled a BGC collection by analysing 150 randomly selected *Actinomycetes* genomes using antiSMASH version 7.0^14^, resulting in the annotation of 2613 BGCs. We then applied the models generated using each of our six manually curated antibiotic BGC families to this collection. To our knowledge, there are no existing automated tools that perform the task of targeted expansion of user defined BGC families, so manual analysis conducted by experienced practitioners was the best method for defining ground-truth and subsequently calculating false positive rate. A total of seven hits were returned across all six BGC families. Only one of these was deemed to be a false positive, indicating relatively high specificity for all six families tested.

### Development and performance of sequence similarity classifiers

To improve sensitivity, especially for fragmented BGCs and out-of-sample cases, we developed two further classifiers based on direct sequence similarity. These were assessed for sensitivity, specificity and robustness to sequencing artefacts as already described (Fig. 2).

The blast-vec method creates “blast-vectors” by treating the cumulative BLAST score of a query BGC against each MIBiG reference as a dimension. Using the same centroid-based approach, this method improved sensitivity but led to a reduction in specificity compared to the Pfam-based method (Fig. 2).

The blast-rank method uses a rank-based similarity metric. It defines a family based on the number of non-self, in-family BLAST hits (*n*) found within a certain rank (*k*) for all known family members. A query BGC is then classified as a hit if it has an in-family BGC as its top hit and at least *n* in-family hits within the top *k* results. The blast-rank method demonstrated improved out-of-train sensitivity and greater robustness to fragmented and concatenated BGCs, with only a slight reduction in specificity compared to the Pfam vector method. Its performance on fragmented data was comparable to or better than the blast-vector method (Fig. 2).

### Development and performance of the predicted product structure classifier

For NRPS/PKS families, we also developed the structural k-mer intersection“method. This method parses antiSMASH JSON outputs to extract a linearized monomer sequence for each ORF in a BGC. These sequences are converted into unique k-mer feature sets. For classification, a query BGC’s k-mer set is compared against reference families, and it is assigned to the family with the best k-mer overlap, provided the score exceeds a pre-optimized threshold. This approach performed well, with recall values ranging between 0.82 and 0.96 for the five antibiotic families tested in our leave-one-out analysis (Fig. 2). Specificity was also high, with just one false positive returned across all five antibiotic families when tested using our test collection of 150 actinomycetes genomes (2613 BGCs). The k-mer intersection method performed relatively well when tested using concatemers of target BGCs. In contrast, the method’s performance degraded significantly on smaller BGC fragments (Fig. 2). This was expected, as fragmented clusters often lack the complete set of megasynth(et)ase modules required to reconstruct a meaningful k-mer profile, thereby precluding accurate classification based on product structure.

### Development of a flexible ensemble method for intuitive automated analysis

Evaluation of the algorithms we developed for BGC classification was confounded by the lack of a complete ‘gold standard’ dataset with known true negatives and false negatives, a common issue in the development of novel bioinformatics tools. The MIBiG database provides an excellent source of characterised BGCs to serve as labelled training data, however the relative scarcity of characterised BGCs means that partitioning this into test/train sets for cross validation is not feasible. Therefore, we evaluated the effectiveness of the component algorithms that were subsequently combined in DiscERN using a multi-faceted approach focused on the metrics most relevant to a real-world discovery scenario: sensitivity to known family members using leave-one-out, robustness to common data artifacts, and the specificity of predictions on an uncharacterized dataset. The four approaches we developed had variable performance across the six datasets we compiled and tested. In light of this, to leverage the strengths of the different approaches, we combined them into an ensemble method named DiscERN (Discoverer of Evolutionarily Related Natural products).

DiscERN is a command line application, implemented in python, that utilises a combination of all four classification methods along with an optional filtering step based on specific Pfam domain presence/absence. DiscERN allows users to define their own custom families of natural product BGCs for expansion by either specifying the MIBiG BGC numbers for each member, or by providing separate antiSMASH formatted GenBank and JSON files as a reference. It then scans user provided antiSMASH outputs for additional members of the BGC family and outputs a collection of combined GenBank files for the hits, grouped according to the number of algorithms (between 1 and 4) that independently identified a given hit. Hits are additionally segregated into those passing or failing a Pfam content filtering step that is automatically implemented based on common Pfam domains in the BGCs defining the family to be expanded. Outputs can then be optionally passed to a local install of antiSMASH for analysis. The primary outputs of DisCERN are a collection of genbank files, and optionally, collated antiSMASH outputs, that are grouped according to likelihood of truly representing on-target BGCs. These outputs are intended to facilitate selection of targets for heterologous expression or compound isolation studies. DiscERN can be installed for Linux and MacOSX and is available at https://github.com/MaxMeta/DiscERN.

### Performance of the DiscERN ensemble on a real-world discovery task

To assess performance in a realistic discovery scenario, we applied DiscERN to a large dataset of 3561 publicly available Actinomycete genomes. To construct this dataset, we queried the NCBI Assembly Database^43^ for all genomes belonging to the class Actinomycetes and refined the resulting list to include only those designated as ‘representative’ or ‘reference’ genomes. Downloaded fasta files were then processed with antiSMASH 7.0 to yield 59,236 BGCs.

**Fig. 3:**
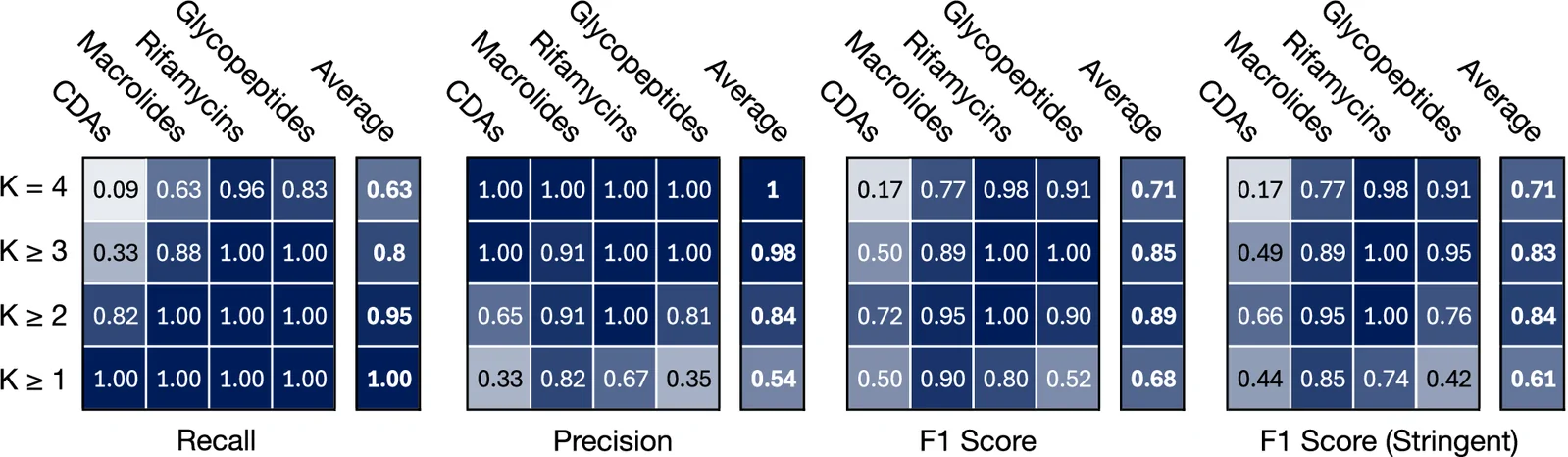
Performance of the DiscERN ensemble. From left to right, heat maps indicate: Recall as assessed by leave one out cross validation; precision determined by manual examination of putative hits; F1-scores calculated from respective recall and precision values at each confidence tier; stringent F1 scores calculated by treating all indeterminant hits as false positives. Source data are provided in the Source Data file.

DiscERN was used to parse antiSMASH outputs for our collection of 3561 genomes using four different antibiotic families as references: calcium-dependent lipopeptides, glycopeptides, rifamycin like polyketides and 16-membered macrolides. This search returned 688 putative hits, which were ranked based on the number of algorithms in the DiscERN ensemble providing support (a confidence score of k = 1–4). To measure the precision of these confidence tiers, we manually curated hits for each family and tier to assign hits as either true-positives, false-positives. Hits not able to be determined owing to fragmentation were excluded from the analysis. This analysis revealed a clear trend: the precision of a prediction increased markedly with the level of ensemble agreement (Fig. 3, Supplementary Data 2, Supplementary Data 3).

Owing to the large number of BGCs in our Actinomycete dataset, complete manual analysis to count false negatives was not feasible. In lieu of this, we estimated recall for four benchmark BGC families using the leave-one-out (LOO) cross-validation framework previously conducted for each of the algorithms individually. For each fold of the LOO analysis, the held-out BGC was classified by the full DiscERN ensemble, and the number of supporting algorithms (k) was recorded. We then calculated the cumulative recall for each confidence threshold (k ≥ 1 to k = 4) by determining the fraction of family members that were successfully identified at or above that level of ensemble support. This approach provides a robust estimate of the ensemble’s sensitivity and its ability to identify known true positives at different levels of stringency.

Our analysis allowed us to construct a comprehensive performance profile for DiscERN, balancing the precision observed in a real-world discovery scenario with the recall estimated from a curated dataset (Fig. 3). To summarise this trade-off, we calculated estimated F1 scores for each confidence level. The results show that a threshold of k ≥ 2 achieves the highest F1 score (0.89), representing the optimal mathematical balance between a high recall (0.95) and strong precision (0.84). However, for many practical discovery applications where minimising experimental validation of false positives is a primary concern, a higher confidence threshold may be preferable. At k ≥ 3, the precision is excellent (0.98), meaning nearly all predicted hits are true positives. This near-certainty is achieved while maintaining a robust recall of 0.80, resulting in a very strong F1 score of 0.85. This demonstrates that DiscERN can be tuned for different discovery goals: a threshold of k ≥ 2 provides the optimal balance between precision and recall, while k ≥ 3 offers a highly precise set of candidates that may be preferred if minimising downstream manual analysis is a priority. We also calculated a “worst case scenario” outcome, treating all hits classified as N/D as false positive. Even under this more stringent estimate DiscERN still performed well with average F1 scores of 0.83 and 0.84 at k ≥ 3 and k ≥ 2, respectively (Fig. 3).

The relatively weaker performance for the calcium-dependent antibiotic (CDA) family likely reflect its unusually high biosynthetic and chemical diversity. CDA BGCs are distributed across multiple actinomycete genera and often share relatively low sequence identity despite producing cyclic lipopeptides with related bioactivity and conserved structural features, suggesting ancient divergence from an ancestral BGC. In addition, CDA clusters lack a small set of consistently co-occurring, family-specific biosynthetic markers that are rare outside the family, which reduces the strength of discriminative signals relative to more tightly conserved families.

### Utility of DiscERN for expansion of RiPP families

To further test the generality of DiscERN for targeted BGC family expansion, we examined the thiostrepton-like family of thiopeptide antibiotics. In addition to providing another example outside of the NRPS and PKS BGCs, the thiostrepton like BGCs were chosen as they share a conserved peptide core which would readily allow any hits to be classified as false positives or true positives, as well as allowing conservative assignment of novelty based on differences in core sequence when compared to previously characterised thiostrepton-like compounds. At the time of conducting this analysis, there were five thiostrepton-like BGCs in the MIBiG repository of characterised BGCs (Supplementary Data 1), each of which contained similar core sequences, cluster architecture and collection accessory genes. Interrogation of our collection of 3561 actinomycetes genomes with this collection returned eleven hits, nine of which were assigned high confidence (k ≥ 3). Manual examination revealed that ten of the eleven hits contained a clearly identifiable peptide precursor gene, with the remaining fragmented BGC lacking precursor (Supplementary Data 9). Extracting the peptide core sequences from the ten precursor genes and aligning these against the known reference compounds revealed that eight of the ten were an exact match for one or more of the known compound cores. The remaining two cores differed from their closest matches at one and two positions, respectively (Supplementary Fig. 15).

### Visualizing BGC relatedness to identify novel compound candidates

DiscERN generates several outputs to facilitate hit prioritization. It calculates a mean pairwise distance matrix that integrates all three similarity measures (sequence similarity, Pfam content, and, where applicable, predicted product structure). This matrix, as well as the individual matrices for each distance metric, are used to construct dendrograms that intuitively display the degree of relatedness among the hit BGCs and the reference BGCs used to define the family. The combined matrix is also provided as a Newick formatted file for visualization in external tools like iTOL^44^.

**Fig. 4:**
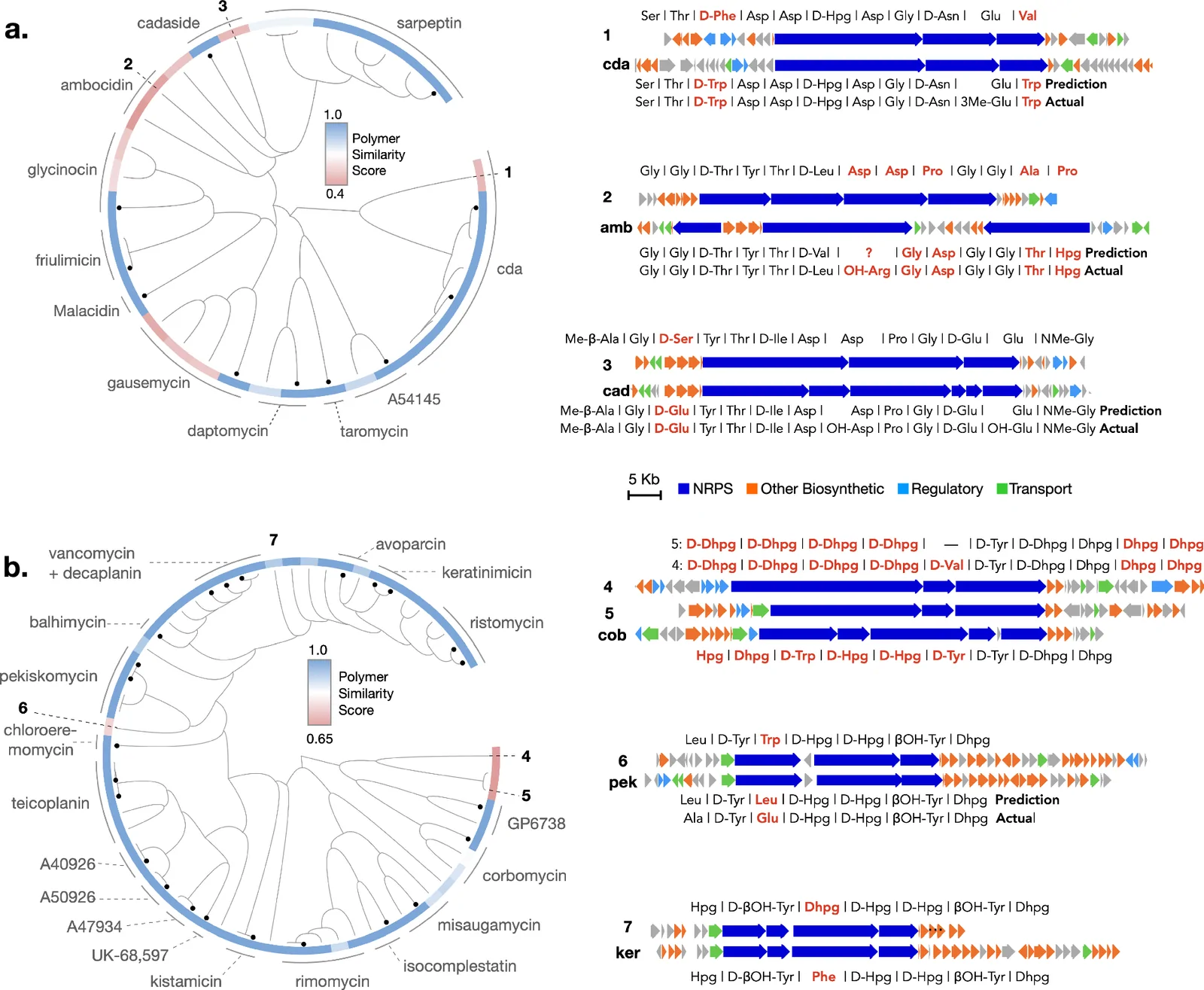
Visualisation of BGC relatedness using DiscERN. Putative hits for lipopeptide (**a**) and glycopeptide (**b**) antibiotics are shown. Left hand panels show hierarchical clustering results calculated by DiscERN using an average three distance metrics. The coloured bars at the end of each leaf indicate polymer similarity score, as determined by the kmer intersection algorithm implemented as part of the DiscERN ensemble. Black dots at leaves indicate the location of previously characterised reference BGCs that are used to define clades. Right hand panel shows selected BGCs predicted to encode new family members. Numbers match leaf labels. Predicted differences in NRPS specificity are highlighted in red.

When applied to the four BGC families from our Actinomycetes analysis, this feature allowed us to clearly identify distinct sub-clusters and outliers that were strong candidates for producing uncharacterised family members (Fig. 4, Supplementary Fig. 14). In the case of the glycopeptides, the combined tree constructed by DiscERN clearly delineates the five previously described compound classes^45^. This delineation most closely resembles the relationship produced in phylogenies based on concatenated A-domain specificity codes^46^.

To further investigate candidates for potentially new compounds, we compared the predicted NRPS/PKS domain compositions and substrate specificities of selected hits against their closest characterised relatives. We found 29 BGCs that had substrate specificities and/or domain composition that clearly differed from their closest characterised relative, suggesting they encode novel compounds, and 85 BGCs that had identical domain composition and substrate specificity to their closest relative (Fig. 4, Supplementary Data 2, Supplementary Data 3). This number is likely a conservative estimate of novelty, as this analysis does not account for variations in post-scaffold tailoring reactions, which are common in families like the rifamycins and glycopeptides.

### Discovery of the discomycin BGC

Among the collection of BGCs identified as potentially encoding new bioactive compounds, a putative hit in the CDA family located within the genome of *S. kanamyceticus* ATCC12853 was chosen for further study (Fig. 4a, BGC-1). This BGC, which we name the discomycin (*dsc)* BGC was chosen as it was present in a commercially available strain with no previous reports of calcium dependent antibiotic activity, and contained a SARP gene that suggested a plausible means for activation^47^.

**Fig. 5:**
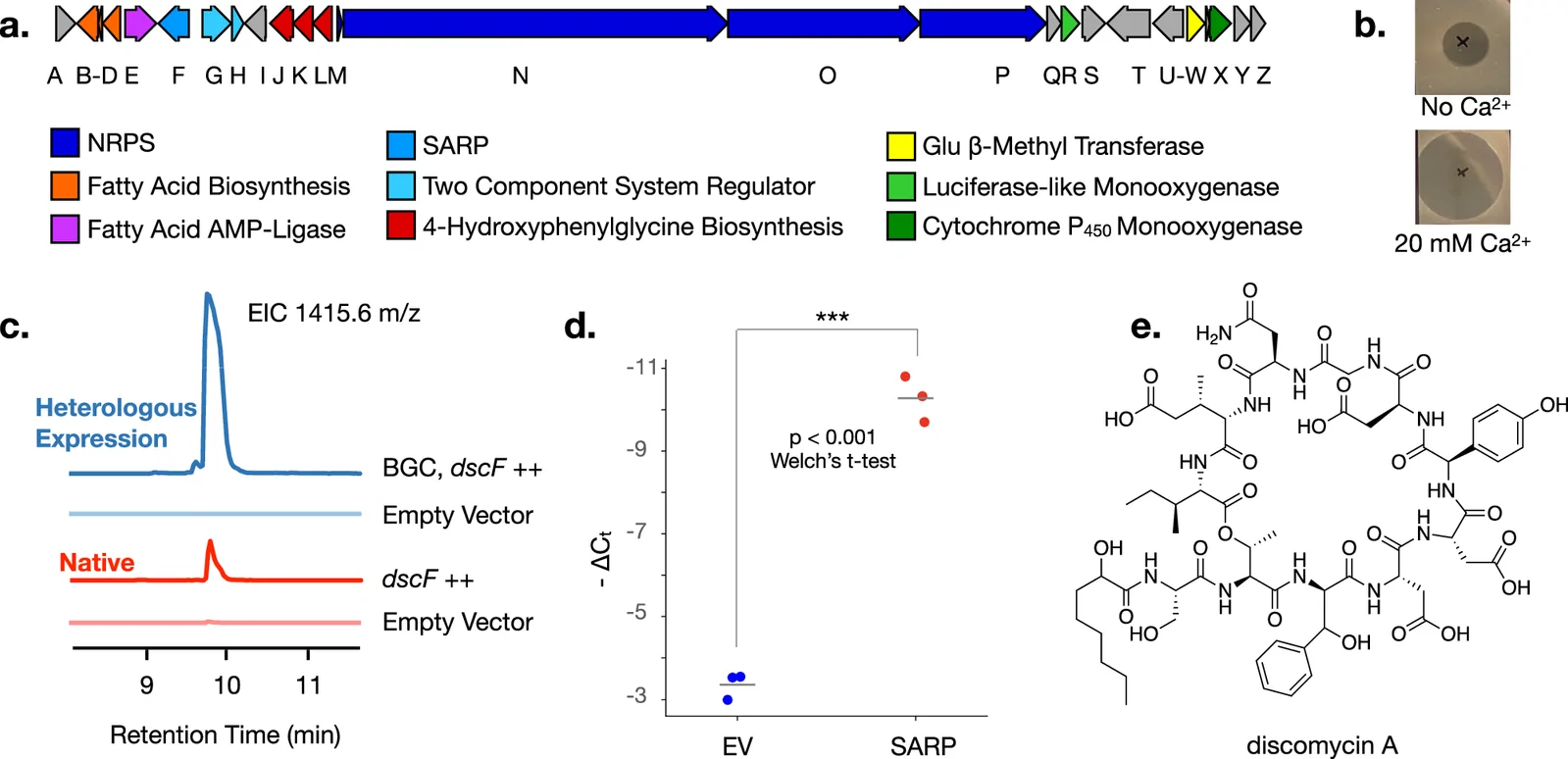
Discovery of discomycin A using DiscERN. **a** Map of the discomycin BGC predicted to encode a new calcium dependent lipopeptide antibiotic. **b** Zone of inhibition assays showing calcium dependent antibiotic activity of crude extract from a culture of S. albus-del14 harbouring the discomycin BGC and an additional copy of the SARP gene *dscF* under the control of a strong constitutive promoter. **c** LC-MS analysis of crude extracts from *S. kanamyceticus* and *S. albus*-del14 harbouring the discomycin BGC, each with an additional copy of the SARP gene *dscF* under the control of a strong constitutive promoter. **d** NRPS expression (qPCR) in EV and SARP strains. Ct values for NRPS were normalised to gyrA; data are plotted as −ΔCt. Points show means of means of technical triplicates for each of three biological replicates, black bars indicate the mean of biological replicates. Significance was tested using Welch’s *t* test on *Δ*C_t_ values (unpaired, *p* = 0.0002, two-sided). **e** Elucidated structure for discomycin A. Source data are provided in the Source Data file.

The *dsc* BGC has clear similarity with the *cda* BGC from *S. coelicolor*. Both feature an 11-module NRPS architecture split across three contiguous genes. The *dsc* BGC contains the expected genes for for 4-hydroxyphenylglycine (Hpg) biosynthesis (*dscAJKL)* and glutamate 3-methylation (*dscV*). However, predicted A-domain specificities suggest the *dsc* BGC encodes a novel lipodepsipeptide, differing from CDA at residues 3, 9, and 11 (Figs. 4a and 5a).

Like the *cda* BGC, the *dsc* BGC contains a dedicated Type II fatty acid synthase (FAS) system for synthesizing its lipid tail, including genes for chain initiation (*dscC*, KAS III), extension (*dscB*, KAS I), and an acyl carrier protein (*dscD*, ACP). The *dsc* cluster lacks the desaturase (*hxcO*) and epoxidase (*hcmO*) homologues required to produce the 2,3-epoxyhexanoyl moiety of CDA and possess a fatty acyl-AMP ligase (FAAL) gene, *dscE*. The presence of this FAAL suggests a hybrid lipidation mechanism, distinct from both the canonical CDA and daptomycin pathways. We hypothesize that the fatty acid is first synthesized by the on-pathway FAS system and then released, after which it is re-activated as an adenylate by the FAAL before being transferred to the NRPS assembly line.

### Activation of the silent discomycin BGC

To activate the silent *dsc* BGC, we first integrated an additional copy of the SARP gene, *dscF*, into the chromosome of the native producer, *S. kanamyceticus*, under the control of a strong constitutive promoter to give the strain *S. kanamyceticus::dscF*. Comparison of organic extracts from *S. kanamyceticus::dscF* to an empty vector control using LC-MS revealed the production of a new compound with a [M + H]^+^ ion at *m/z* 1415.6023 (Fig. 5c). To confirm that *dscF* overexpression transcriptionally activates the cluster, we performed RT-qPCR on cDNA from S. kanamyceticus::*dscF* and an empty-vector control, using primers targeting the first NRPS gene in the dsc assembly line and normalising to the housekeeping gene *gyrA*. This analysis showed a strong induction of NRPS transcript levels in the dscF strain (≈120-fold relative to control, Fig. 5d), consistent with SARP-mediated activation of the dsc BGC.

To definitively link this compound to the BGC, we cloned the entire *dsc* cluster using the CRISPR-Cas12a CAPTURE method^48^ and expressed it in the genome-minimized host *S. albus* Del14^49^. This resulted in calcium dependent antibiotic activity in crude extracts from the resulting heterologous expression strain (Fig. 5b) and LC-MS/MS analysis of this recombinant strain revealed an ion with an identical *m/z* and MS² fragmentation pattern to that observed in the native host, confirming that discomycin A is the direct product of the *dsc* BGC.

Finally, to optimise production for isolation and structure elucidation, we introduced the *dscF* overexpression cassette into the heterologous host. The resulting strain, *S. albus* Del14::*dsc-dscF*, had higher production of discomycin A than either the heterologous host alone, *or S. kanamyceticus::dscF*, and was therefore selected for large-scale fermentation (Fig. 5c)

### Isolation and characterisation of discomycin A

For structure elucidation, discomycin A was isolated from *S. albus* Del14::*dsc-dscF* grown in liquid TSB medium containing HP-20 resin. The compound was purified from the methanolic resin extracts of the harvested HP-20 resin using calcium-mediated precipitation followed by reversed-phase HPLC. Its planar structure was then elucidated using a combination of HRESI-MS/MS, 1D, and 2D NMR experiments (Supplementary Table 3, Supplementary Figs. 4–13).

The data revealed that discomycin A is a cyclic lipodepsipeptide containing 11 amino acids with an N-terminal 2-hydroxyoctanoyl tail. A 31-membered macrolactone ring is formed between the hydroxyl of Thr² and the C-terminal carboxyl of Ile¹¹. The elucidated peptide sequence—Ser¹-Thr²−3OHPhe³-Asp⁴-Asp⁵-Hpg⁶-Asp⁷-Gly⁸-Asn⁹−3MeGlu¹⁰-Ile¹¹—was in full agreement with the antiSMASH predictions for the *dsc* BGC. The structure of discomycin A was further supported by MS² fragmentation of the NaOMe linearised the product (Supplementary Fig. 6).

Finally, to determine the absolute stereochemistry of discomycin A, we performed C3 Marfey’s analysis^50^ on the hydrolysed peptide (Supplementary Fig. 7, Supplementary Table 4). This confirmed the presence of D-Phe³, D-Hpg⁶, and D-Asn⁹, consistent with the epimerase domains predicted in the corresponding NRPS modules and allowed assignment of absolute configurations for all stereo centres except the β-carbon of 3-OH-Phe and C2 of the lipid tail.

### Spectrum of activity of discomycin A

**Fig. 6:**
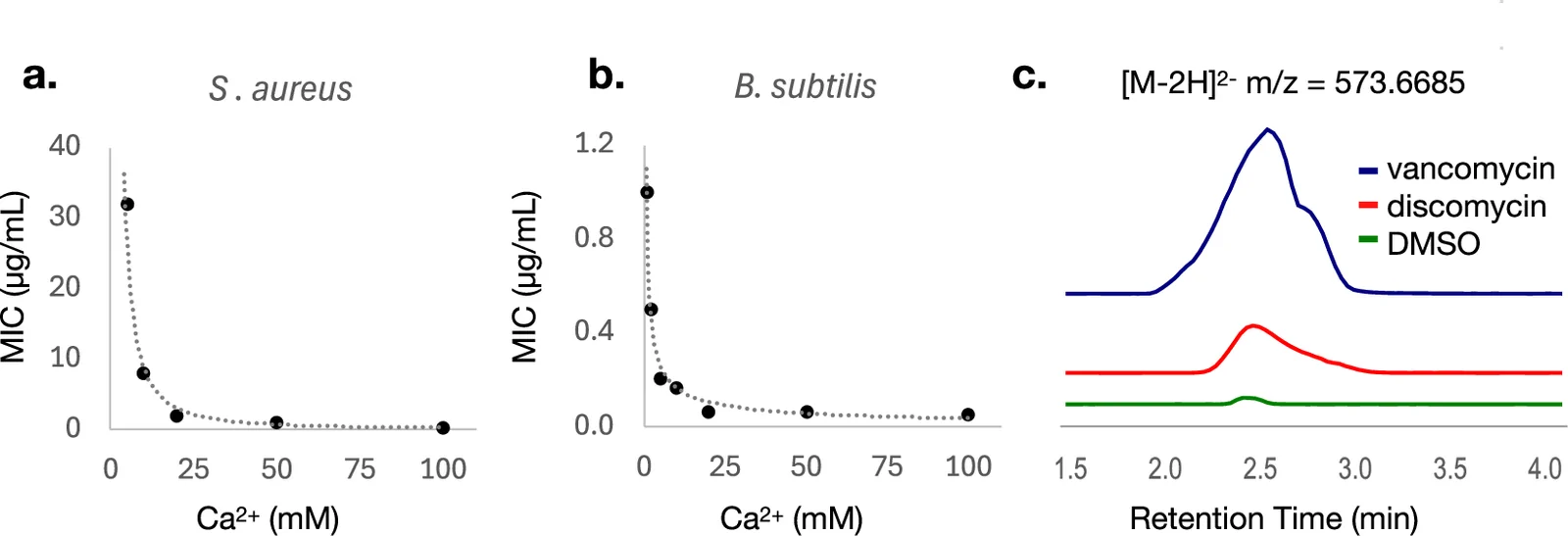
Discomycin has calcium-dependent antibiotic activity against Gram-positive bacteria and inhibits cell-wall biosynthesis. **a** Average MIC values from triplicate assays against *S. aureus* at various concentrations of Ca^2+^. **b** Average MIC values from triplicate assays against *B. subtilis* at various concentrations of Ca^2+^. **c** HPLC-HRMS analysis of cell lysate from *S. aureus* cultivated in the presence of discomycin A, DMSO (negative control) or vancomycin (positive control), monitoring the [M-2H]^2-^ ion of UDP-MurNAc-pentapeptide (m/z = 573.6685) in ESI negative mode. Source data are provided in the Source Data file.

The biological activity of purified discomycin A was evaluated using broth microdilution assays against a panel of Gram-positive and Gram-negative bacteria, alongside a cytotoxicity assessment against the HCT-116 human colon carcinoma cell line (Supplementary Table S5). Discomycin A exhibited potent, Ca^2+^-dependent antibacterial activity against a range of Gram-positive pathogens (Fig. 6a, Supplementary Table S5). with minimal inhibitory concentrations (MICs) in the low-micromolar range. In contrast, no antibacterial activity was observed against any of the Gram-negative bacteria in our panel, even at the highest tested concentration. Furthermore, discomycin A displayed no discernible cytotoxicity against HCT-116 cells, indicating a favourable selectivity for bacterial over mammalian cells. We also treated *S. aureus* with a sub-inhibitory concentration of compound. This lead to accumulation of N-acetylmuramic acid pentapeptide, indicating inhibition of cell wall biosynthesis (Fig. 6c).

### Benchmarking against BiG-SCAPE and GATOR-GC for CDA family expansion

We benchmarked DiscERN against two recently developed tools that are also capable of multi-reference interrogation of biosynthetic space: BiG-SCAPE^16,27^ and GATOR-GC^29^. Our benchmarking experiments comprised a targeted family expansion task on 573 Streptomyces genomes (19,521 antiSMASH BGCs), with an identical CDA reference definition for all methods (11 experimentally validated CDA-family BGCs from MIBiG). For BiG-SCAPE, we clustered the *Streptomyces* BGC set together with the all MIBiG 4.0 BGCs across a range of clustering-distance cutoffs. For each cutoff, we extracted all BGCs connected to one or more of our 11 CDA reference BGCs and manually classified the resulting dataset. BiG-SCAPE achieved its best performance at a clustering cutoff of 0.4, yielding high precision but limited recall (Supplementary Table S6).

For GATOR-GC, the raw output comprises many focal-window alignments and does not directly provide a ranked candidate list suitable for prioritisation. We therefore implemented a minimal post-processing python script to (i) retain only query BGCs that matched at least one CDA reference and (ii) collapse multiple alignments per query to the single best-scoring reference match. This yielded a comparable ranked hit list for downstream evaluation. Manual annotation of the top-ranked GATOR hits revealed a clear optimal score threshold. Above this threshold the candidate set remained enriched for true positives, while relaxing the threshold produced a disproportionate increase in false positives for minimal true-positive gain (Supplementary Table 7).

DiscERN outperformed both comparator tools in this task, achieving an F1-score of 0.72 versus maximal F1-scores of 0.55 and 0.49 for BiG-SCAPE and GATOR-GC, respectively. DiscERN also returned more of true positives with a higher proportion of putatively novel peptide backbones than either comparator tool (Table 1, Supplementary Tables 6, 7). BiG-SCAPE did not identify the discomycin BGC, even at the most relaxed clustering threshold, and the BiG-SCAPE hits recovered across all clustering thresholds were a strict subset of those identified by DiscERN. GATOR-GC did correctly identify the discomycin BGC and returned one hit which was not identified by either DiscERN or BiG-SCAPE.

DiscERN is a multi-modal bioinformatics tool that solves a critical challenge in genome mining: the targeted, hypothesis-driven expansion of specific natural product families. By integrating four complementary algorithms, DiscERN allows users to strategically balance discovery sensitivity with predictive precision, creating a direct and reliable path from genomic data to a prioritised list of high-confidence candidate BGCs. This tuneable approach serves diverse research goals, from exhaustive screening where no potential hit can be missed, to high-precision workflows where minimising downstream analysis and experimental effort is paramount.

We have validated this entire pipeline not only through comprehensive computational benchmarking, but also by prediction-led discovery of the novel antibiotic discomycin A from a silent BGC. The journey from an in silico prediction to an isolated and structurally characterised molecule provides a concrete demonstration of DiscERN’s real-world utility. This work therefore demonstrates both a powerful and accessible new tool for the natural products community and a clear workflow for how integrated computational and synthetic biology approaches can effectively translate the promise of the genomics era into new chemical entities.

## Methods

### Annotation of actinomycetes genomes using antiSMASH 7.0

Genomes for antiSMASH analysis were downloaded from the NCBI Assembly database using a python script that queried for all genomes belonging to the class Actinomycetes and refined the resulting list to include only those designated as ‘representative’ or ‘reference’ genomes. Downloaded genomes were then distributed into subdirectories and annotated using a local install antiSMASH 7.0 with the ‘knownclusterblast’ function enabled.

### Compilation of reference data for DiscERN

Reference BGC data for DiscERN was compiled by downloading all MiBiG entries as GenBank files (Version 3.1 (October 7, 2022) and analysing these with antiSMASH 7.0.

### Performance evaluation of the DiscERN ensemble

The performance of the DiscERN ensemble was evaluated using a multi-faceted approach to robustly estimate its effectiveness in a real-world discovery scenario. We focused on two key metrics: Recall, estimated via a Leave-One-Out (LOO) cross-validation on curated datasets, and Precision, estimated by manually curating predictions from a large, uncharacterized genomic dataset. All performance metrics were calculated independently for each of the six benchmark BGC families, and the unweighted mean of these results was also calculated to provide a measure of performance on an “average” BGC family, preventing bias from families with a higher or lower number of members or hits.

### Recall estimation via Leave-One-Out (LOO) cross-validation

To estimate the sensitivity of the ensemble at different confidence thresholds, a LOO cross-validation was performed for each of the six benchmark BGC families. For a given family containing *N* members, the process was repeated *N* times. In each fold *i*, the *i*-th BGC was held out while the remaining *N-1* BGCs were used to define the family model. The held-out BGC was then classified, and the number of algorithms supporting its correct identification, ${k}_{{hits}}$, was recorded.

The recall for a given confidence threshold *k* (where *k* ∈ {1, 2, 3, 4}) was calculated as the fraction of BGCs in the family that were successfully identified by *k* or more algorithms. Let $T{P}_{k}$ be the count of true positives identified by at least *k* algorithms. The recall, ${R}_{k}$, is given by:

1 $${R}_{k}=\frac{T{P}_{k}}{N}=\frac{\mathop{\sum }_{i=1}^{N}{\mathbb{I}}\left({k}_{{hits},i}\ge k\right)}{N}$$

where II(*condition*) is the indicator function, which is 1 if the condition is true and 0 otherwise.

### Precision estimation and handling of undetermined cases

To estimate precision, DiscERN was applied to a large dataset of 59,236 BGCs derived from 3561 Actinomycete genomes. For each confidence tier *k* (where hits are supported by exactly *k* algorithms), putative hits were manually curated. Owing to large numbers of hits, especially at lower confidence tiers, a maximum of 20 hits were examined for each confidence tier.

Each BGC was manually classified as a True Positive (TP), False Positive (FP), or Not Determined (ND) if fragmentation prevented a confident assignment. BGCs were classified as FP according to the following criteria

**TP:** Family-defining biosynthetic architecture/core features present. Some examples of such features are X-domains and cross-linking enzymes in glycopeptides. Predicted number of amino acids in peptide backbones or carbons in polyketide backbones. Predicted presence and spacing of D-configured alpha-carbons, predicted presence of key functional groups (for example acidic groups in CDA families and aromatic amino acids in glycopeptides).

**FP:** Incompatible architecture, substrate specificity and/or core features as described above or missing critical core features.

**N/D:** insufficient information due to fragmentation/boundary ambiguity. Our analysis also includes a conservative estimate of performance where all such cases are treated as FP (see Fig. 3, panel labelled “F1-score, stringent”.

To calculate a final precision estimate for each tier, we first calculated the precision based only on the subset of classifiable hits. To account for the ND cases, we calculated two sets of precision estimates: a primary estimate and a conservative “worst-case” estimate.

Let, for each tier *k:*

- ${N}_{k}$ = Total number of hits predicted by DiscERN.
- $t{p}_{k},f{p}_{k},n{d}_{k}$ = The counts of TPs and FPs and NDs, respectively.

In cases where ${N}_{k} > 20$, the precision was estimated using 20 randomly selected hits. Otherwise, the entire collection of hits was used. Our primary estimate assumes that the ND cases are distributed in the same proportion as the classifiable cases. Here, the precision for the classifiable subset, ${P}_{\det,k}$, was first calculated:

2 $${P}_{det,k}=\frac{t{p}_{k}}{t{p}_{k}+f{p}_{k}}$$

This precision was then applied to the entire population of ${N}_{k}$ hits for that tier to estimate the total adjusted True Positive ($\widehat{{{TP}}_{k}}$) and False Positive ($\widehat{{{FP}}_{k}}$) counts. This approach assumes that the ND cases are distributed in the same proportion as the classifiable cases.

3 $$\widehat{{{TP}}_{k}}={{N}_{k} \times P}_{det,k}$$

4 $$\widehat{F{P}_{k}}={{N}_{k}} \times (1-P_{det,k})$$

For our conservative “worst-case” estimate, we assumed that all ND cases were False Positives. In this scenario, the total True Positives are only those directly observed and extrapolated, while False Positives include both observed FPs and all NDs. The conservative precision, ${P}_{{cons},k}$, is thus calculated from the sample counts as:

5 $${P}_{{cons},k}=\frac{t{p}_{k}}{t{p}_{k}+f{p}_{k}+n{d}_{k}}$$

The corresponding conservative estimates for the total population were then calculated by applying this rate to the *N*_*k*_ hits.

### Calculation of cumulative precision and F1 score

Using both sets of adjusted counts (primary and conservative), we calculated the cumulative precision $({CP}_{k})$ for a threshold of *k* is given as:

6 $${{CP}}_{k}=\frac{{\sum }_{i=k}^{4}\widehat{T{P}_{i}}}{{\sum }_{i=k}^{4}(\widehat{T{P}_{i}}+\widehat{F{P}_{i}})}$$

So, for example:

7 $${{CP}}_{3}=\frac{\widehat{T{P}_{3}}+\widehat{T{P}_{4}}}{\widehat{T{P}_{3}}+\widehat{T{P}_{4}}+\widehat{F{P}_{3}}+\widehat{F{P}_{4}}}$$

To provide a single metric summarizing the balance between precision and recall, an estimated F1 score was calculated for each cumulative threshold. The F1 score, $F{1}_{k}$, is the harmonic mean of the estimated cumulative precision (${{CP}}_{k}$) and the estimated recall from the LOO analysis $({R}_{k})$:

8 $$F{1}_{k}=2\times \frac{{{CP}}_{k}\times {R}_{k}}{{{CP}}_{k}+{R}_{k}}$$

### BGC cloning and heterologous expression

The discomycin BGC boundary was chosen by comparison to the known boundaries of the CDA BGC from Streptomyces coelicolor. CRISPR RNAs (crRNAs) for Cas12a excision of the targeted BGC flanking sequence were designed and prepared according to previous published protocol^48^. Briefly, crRNA was synthesised using HiScribe T7 Quick High-Yield RNA Synthesis Kit (NEB) from an annealed dsDNA oligo template with unique spacer sequence targeting either upstream or downstream of the BGC. The BGC was then cloned from purified genomic DNA using Cas12a directed capture cloning.

CRISPR-Cas12a-mediated direct capture method^48^ was used with modification. Briefly, the blue-white screening *lacZ* gene in pBE45 vector was replaced with PheS negative selection marker with strong constitutive promoter J23100 and pET-RBS. This reduces empty background colonies during cloning stage since empty vector colonies would express the PheS gene and exerts negative selection pressure in presence of suicide substrate 4-chlorophenylalanine. N-terminal His6-FnCas12a was purified from IPTG-induced BL21 DE3 *E. coli* cells harbouring the expression construct pET-FnCas12a-TEV (gifted by Huimin Zhao) using HisTrap column on a AKTA Pure FPLC system and stored as 10 µM aliquots in storage buffer (20 mM Tris-HCl pH 8, 150 mM NaCl, 15% (v/v) glycerol) at −80 °C. The genomic DNA was prepared from *S. kanamyceticus* ATCC12853 using the salting out method^51^ and digested using Cas12a guided by crRNA targeting BGC flanking sequences.

PCR fragments amplified from pBE48 and modified pBE45 (pBE45-*pheS*) containing homologous arms to the *dsc* cluster were mixed with digested gDNA for ligation by T4 DNA exo + fill-in protocol as described previously^48^. The ligation mixture was desalted then transformed into electrocompetent NEB10b containing the pBE14 helper plasmid and plated on LB agar with 100 µg/mL apramycin, 0.2% D-glucose and 2.5 mM 4-chlorophenylalanine. Colonies were checked using BGC-specific primers to verify presence of BGC, and cloned BGC plasmids were purified using NucleoBond Xtra Midi kit (Macherey-Nagel) and verified by KpnI-HF (NEB) restriction digest. Verified BGC plasmid was conjugated into *S. albus* Del14 via ET12567/pUZ8002 *E. coli* conjugation donor strain, generating Del14::dsc.

Exconjugants were recovered on fresh ISP4 (Himedia laboratories), re-streaked onto ISP4 plates containing, 50 μg/mL apramycin and 50 μg/mL nalidixic acid, and verifying the presence of the integrated *dsc* cluster by PCR. Spore stocks were made and stored at −80 °C in 10 % glycerol. All PCR cloning steps used Q5 High-Fidelity 2X Master Mix (NEB). For colony screening BioMix Red 2X reaction mix (Bioline) was used. All oligonucleotides used are listed in Supplementary Table S1. Antibiotic selection was performed using the following concentrations: kanamycin 50 µg/mL, apramycin 50 µg/mL, spectinomycin 50 µg/mL, nalidixic acid 50 µg/mL, ampicillin 100 µg/mL.

### Cloning of SARP-activation plasmid and heterologous expression

SARP gene (*dscF*) associated with *dsc* BGC was amplified from *S. kanamyceticus* genomic DNA with Q5 HiFi DNA Polymerase (NEB) and cloned downstream of a strong constitutive promoter A21RS into pOJ436 (with PhiC31 integrase system) or pIJ10257 (with PhiBT1 integrase system) with NEBuilder HiFi Assembly Master mix. Cloned plasmids were verified with PCR and Sanger sequencing before conjugating into *S. kanamyceticus* (PhiC31) or Del14*::dsc*(PhiBT1) via ET12567 *E. coli* conjugation donor strain, generating Skan::dsc*F* and Del14::*dsc-dscF*, respectively. Exconjugants were recovered from fresh ISP4 re-streaked plates supplemented with appropriate antibiotics (PhiBT1: 100 μg/mL hygromycin; PhiC31: 50 μg/mL apramycin; both: 50 μg/mL nalidixic acid) and checked with PCR to verify BGC integration, and spore stocks were harvested and stored at −80 °C.

### Isolation of discomycin A

A single colony colonies of Del14::*dsc-dscF* (PhiBT1) from 5-day old ISP4 agar was inoculated into 50 mL autoclaved tryptic soy broth (TSB) media and incubated at 30 °C 200 rpm for 2 days. Pre-culture was visually inspected for contamination before inoculating 0.5% (v/v) into 10 × 1 L liquid TSB medium in baffled 2.5 L culture flasks containing 25 g/flask sterile HP-20 resin incubated Fermentation was allowed to proceed at 30 °C for 10 days before collection of HP-20 resin by filtration. HP-20 was washed extensively with distilled water and packed into a glass column. The column was then sequentially eluted with 500 mL each of 20, 40, 60, 80 and 100% acetonitrile. Fractions were then checked for calcium-dependent antibiotic activity, which was found predominantly in the 60% fraction. This was evaporated to dryness under reduced pressure and redissolved in 15 mL of aqueous 0.1% (v/v) triethylamine. Insoluble compounds were removed by centrifugation, before addition of CaCl_2_ to a final concentration of 100 mM to induce precipitation of discomycin. After a 20 min incubation on ice, The precipitated discomycins were collected by filtration and the aqueous phase decanted. The precipitated discomycins were then washed using acetonitrile to remove lipids before being dissolved in a minimal volume of methanol. Semi-preparative HPLC was performed to separate the discomycins using a VP 150/10 Nucleodur C-18, HTEC, 5 µm column (Macherey-Nagel, Germany) on a 1260 Infinity II HPLC (Agilent, USA).

### Marfey’s analysis of discomycin A

*N*α-(2,4-Dinitro-5-fluorophenyl)-L-alaninamide (L-FDAA) and *N*α-(2,4-dinitro-5-fluorophenyl)-D-alaninamide (D-FDAA) were purchased from Santa Cruz Biotechnology. Amino acids and standards were purchased from Chem-impex int’l Inc, Sigma, or Merck. HPLC-grade MeCN and MeOH were degassed and filtered through a 0.45 μm polytetrafluoroethylene (PTFE) membrane prior to use. Water for HPLC was filtered through High-Q 103S Still. All other chemicals and solvents were of analytical grade. A sample of discomycin A (500 μg) in 6 M HCl (500 μL) was heated to 100 °C in a sealed vial for 48 h (monitored by mass spec), after which the hydrolysate was concentrated to dryness via reduced pressure. The hydrolysate was then treated with 1 M NaHCO3 (100 μL) and the sample was split into two even fractions. To each L-FDAA or D-FDAA (1% solution in acetone, 500 μL) was added and heated at 40 °C for 1 h, after which the reaction was neutralised using 1 M HCl (50 μL), diluted with ACN (500 μL) and filtered (0.45 μm PTFE) prior to HPLC-DAD-ESIMS analysis. An aliquot (10 μL) of each analyte was injected into an Agilent Zorbax SB-C3 column, 5 μm, 150 × 4.6 mm, 50 °C, with a 1 mL/min, 55 min linear gradient elution from 15% to 60% MeOH/H_2_O with a 5% isocratic modifier of 1% formic acid in MeCN (5-min hold at 60%, 5-min re-equilibration at 15%). The analyte amino acid content was assessed by UV (340 nm) and ESI(+)MS monitoring, compared to authentic standards. D/L-Amino acids (L-Ser, L-Thr, L-Allo-Thr, L-Asp, D-Hpg, Gly, L-Asn, L-Ilu, L-Allo-Ilu) standards (50 μl of 50 μM solutions) were dissolved in 20 μL of 1 M NaHCO_3_. This was done in duplicate, and to each L-FDAA or D-FDAA (1% solution in acetone, 100 μL) was added and heated at 40 °C for 1 h, after which the reaction was neutralised with 1 M HCl (20 μL), diluted with MeCN (500 μL) and filtered (0.45 μm PTFE) prior to HPLC-DAD-ESIMS analysis as above. In order to obtain a reference retention time for β-Me-Glu, which was not available for purchase, daptomycin was hydrolysed and analysed as described above.

### Elucidation of the structure of discomycin A

The structure of discomycin A was elucidated using a combination of high-resolution electrospray ionisation mass spectrometry (HRESI-MS), 1D and 2D NMR experiments, and C3 Marfey’s analysis^50^. NMR data was acquired in deuterated methanol at 25 °C on a JEOL JNM-ECZ600R spectrometer equipped with a 5 mm FG/RO digital autotune probe (600 MHz for ^1^H nuclei and 150 MHz for ^13^C nuclei). The residual solvent peak was used as an internal standard (δC 49.00; δH 3.31). An Agilent 6530 Q-TOF coupled to an Agilent 1260 HPLC system was used to obtain HRESI-MS data. The observed mass of discomycin A (*m/z* 1415.5865 [M + H]^+^) corresponds to a molecular formula of C_62_H_87_N_12_O_26_ (calcd, 1415.5849; Δ =1.13 ppm) (Supplementary Fig. 2).

Analysis of the NMR data (COSY, HSQC, HMBC) showed the presence of spin systems attributable to three aspartic acids (Asp), asparagine (Asn), serine (Ser), threonine (Thr), glycine (Gly), and isoleucine (Ilu), β-hydroxyphenylalanine (β-OH-Phe), 4-hydroxylphenylglycine (Hpg) and β-methylglutamate (β-Me-Glu), (Supplementary Fig. 4). The N-terminal acyl substituent was determined to be 2-hydroxyoctanoic acid, consisting of one carbonyl (δc 177.4), one oxymethine (δc 72.9), five methylenes (δc 35.6, 32.9, 30.2, 26.1, 23.7, 14.4) and one methyl (δc 14.4). Elucidation of the lipid tail was achieved via COSY correlations between H-2 (δh 4.07), H_2_−3 (δh 1.77, 1.61), H_2_−4 (δh 1.45, 1.43), H_2_−5(δh 1.30), along with connections between H_2_−7 (δh 1.31) and H_3_−8 (δh 0.89). The overlapping protons of H_2_−5, H_2_−6, H_2_−7 (δh 1.30-1.31) obscured COSY correlations, but connection of these carbons could be established via HMBC correlations from H_3_−8 to C−7 (δc 23.7) and C−6 (δc 32.9) and H_2_−4 to C-5 (δc 30.2) and C-6.

The configuration of chiral centres in each amino acid was defuced using C_3_ Marfey’s analysis(Supplementary Fig. 7, Supplementary Table 4). HMBC correlations from each amino acid alpha protons to the nearby neighbouring carbonyl defined connectivity of the molecule, with the exception Ilu_11_ where HMBC correlations from the beta carbon protons established connectivity (Supplementary Fig. 5). The connectivity was corroborated by ESI-MS/MS fragmentation (Supplementary Fig. 6).

### Broth dilution MIC assays

All MIC bioassays and mechanism of action studies were performed in LB broth supplemented with various concentrations of CaCl_2_.A single colony of indicator strain (*Bacillus subtilis* E168, *Staphylococcus aureus* ATCC25923, methicillin-resistant *Staphylococcus aureus* USA 300, *Acinobacter baumannii* Ab5075, *Escherichia coli* ΔtolC) was resuspended in 1 mL media and the OD600 was measured. Bacteria suspensions were diluted to OD600 = 0.0005 with media and kept aside while two-fold drug dilution plates were prepared in a final volume of 100 µL MHB medium supplemented with CaCl_2_. 50 µL diluted culture of indicator strains were added to corresponding wells. Plates were sealed with sterile breathable membrane, wrapped in aluminium foil, and incubated at 37 °C statically (without shaking) for 18 h. The lowest concentrations of antibiotic that resulted in no visible growth of indicator strains were recorded as the MIC. The broth dilution MIC bioassays were carried out in biological triplicate (*n* = 3).

### Mammalian cytotoxicity assay

Cytotoxicity was assessed against the human carcinoma cell line, HCT116, using a standard 48 h cell proliferation assay. In brief, cells were plated into the wells of a 96-well plate at a seeding density of 1.2 ×10^4^ cells/well before incubating for 24 h (37 °C, 5% CO_2_). Cells were treated with discomycin A at various concentrations then incubated for a further 48 h (*n* = 2–3 independent experiments each comprising of two technical replicates). MTS was added and the plate was incubated for 1–2 h, then the absorbance measured at 480 nm on a spectrophotometer.

### LC-MS/MS analysis

Except where otherwise noted, crude extracts, fractions and purified compounds were dissolved in LC-MS grade MeOH, and filtered with 0.22 µm PTFE syringe filter. Each sample was analysed on an Agilent 1260 Infinity binary pump fitted with Agilent InfinityLab Poroshell 120 Reversed-Phase EC-C18 column, 100 ×2.1 mm, 2.7 µm particle size. Mobile phase A was 99.9% H_2_O/0.1% NH_4_HCO_2_. Mobile phase B was 99.9% MeCN/0.1% HCOOH. The flow rate was 0.4 mL/min with the gradient 0–1 min 5.0% B, 1–20 min 5.0–100% B, 20–23 min 100 % B, 23–28 min 5% B. MS data was collected using an Agilent 6530 Accurate Mass Q–TOF LC/MS. Data was collected in both ESI positive and negative modes with m/z range of 100–3000. The MS source settings used were: capillary voltage of 3500 V, nebulizer gas (N_2_) pressure of 30 psig, ion source temperature of 275 °C, sheath gas temperature of 300 °C and flow of 0.4 mL/min, and the acquisition rate was three spectra/s. The ion isolation width was set wide at 7 amu. Minutes 0–1.5 were sent to waste and minutes 1.5–25 recorded. For untargeted ion fragmentation (auto–MS/MS), collision induced dissociation (CID) energies were dependent on the precursor mass determined by the pre–set equations CID = 2.62*x* + 14.75 (low energy) and CID = 3.93*x* + 22.13 (high energy). The four most intense ions per MS scan were subjected to CID and were actively excluded after three spectra for 0.4 min. For targeted fragmentation of purified compounds were directly injected and optimal CID energy were determined empirically.

### BiG-SCAPE benchmarking experiments

The subset of our 3561 Actinomycete genome dataset belonging to the genus Streptomyces (*n* = 573) was used in benchmarking experiments. Symbolic links for all antiSMASH region genomes were collated in a single directory and the gbk files for the entire MiBiG-4.0 database were added to this directory as a reference. BiG-SCAPE v2.02 was then called on this directory with the following command:

bigscape cluster \

-i <path_to_input_dir> \

-o <path_to_output_dir> \

-p <path_to_pfam_dir> \

-c 24 \

-m 4.0 \

--classify none \

--mix \

--gcf-cutoffs 0.35,0.4,0.45,0.5,0.55,0.6

The resulting mixed clustering tsv files for each cutoff (Supplementary Data 7) were then parsed to extract all query genbank files that were linked to one or more CDA reference BGCs using the python scripts found in the jupyter notebook accompanying this manuscript (Supplementary Data 8). The antiSMASH outputs for the resulting collection of 101 BGCs (Supplementary Data 10) were then manually evaluated to categorise each putative hit as either TP of FP or ND (Supplementary Data 5) as already described. A theoretical positive value of 42.68 total CDA-like BGCs was calculated for the Streptomyces dataset using the observed TP number returned by DiscERN at k ≥ 2 and dividing this by the validated recall for DiscERN with the CDA-family at k ≥ 2 (recall=0.82). All ND data were excluded from subsequent calculations and the number of TP and FP classifications, as well as the theoretical positive value of 42.68 were used to calculate precision, recall and F1 scores at each clustering threshold (Supplementary Table 6).

### GATOR-GC benchmarking experiments

GATOR-GC was called on all antiSMASH identified BGCs from the 573 *Streptomyces* genomes already described and the 11 CDA-family BGCs present in the MiBiG 4.0 database were included as references for subsequent parsing. The required protein file for this analysis contained the first NRPS from the CDA BGC (BGC0000315) as well as the terminal thioesterase from this BGC, and captured an example of all domains universally found in the 11 reference BGCs. The optional protein file contained the acyl-CoA dehydrogenase, SARP, glutamic acid 3-methyltransferase, alpha-beta hydrolase, Type II FAS and FabH proteins from the CDA BGC as well as the fatty-acyl-CoA ligase gene from the daptomycin BGC (BGC0000336). Pre-GATOR-GC was run on the collated genbank files to derived db files and the subsequent command line call for GATOR-GC was:

gator-gc -rq /<path_to_required_protein_fasta> \

-op <path_to_required_protein_fasta> \

-g <glob_pattern_for_query_gbk_files> \

-d <path_to_db_files_from_pre_gator_gc> \

-v \

-o <path_to_output_dir>

The resulting concatenated gator focal score file was then parsed using the python scripts found in the jupyter notebook accompanying this manuscript (Supplementary Data 8) to collate all query gator windows that aligned with one or more CDA reference BGC, as well as the maximum alignment score for each query. AntiSMASH outputs for the top 100 hit BGCs (Supplementary Data 11) were manually examined to classify as TP/FP/ND (Supplementary Data 6), and the gator focal score threshold that maximised F1 score was determined using the workflow and scripts contained in the Jupyter notebook accompanying this manuscript (Supplementary Data 8).

### qPCR analysis of *dsc* BGC induction by SARP overexpression

Spore stocks for *S. kanamyceticus* harbouring the SARP overexpression vector, as well as an empty vector were streaked onto TSB plates supplemented with 50 µg/mL hygromycin. Three single colonies from each plate were then patched onto TSB plates to establish biological triplicates for the EV and SARP conditions. Following four days incubation at 30 °C, ~50 mg mycelium was harvested and suspended in 500 µL Tri-Reagent^®^ (Sigma-Aldrich). 200 µL zirconium beads were then added to each of the six tubes and the samples were lysed by bead beating.

Following lysis, 100 µL chloroform was added to each tube an samples were mixed by gentle inversion. Samples were then centrifuged at 17,000 × *g* for 10 min to separate phases and the upper (aqueous) phase was removed to a fresh tube. Nucleic acids were then precipitated by adding an equal volume of isopropanol, followed by gentle inversion and incubation at room temperature for 10 min. Precipitated nucleic acids were then pelleted by centrifugation (17,000 × *g*, 15 min) and the pellet was washed once with 1 mL 70% ethanol. Washed pellets were airdried for 5 min and resuspended in 50 µL nuclease free water.

Samples were then treated with RNAase free DNAase (2U enzyme, 1x NEB DNAase I reaction buffer, 37 °C, 60 min) before cleanup using an NEB Monarch^®^ Spin RNA Cleanup Kit (50 µg) according to the manufacturer’s directions. Following clean-up, purified RNA was quantitated, and reverse transcription reactions were carried out using NEB LunaScript^®^ RT SuperMix according to the manufacturer’s directions, with no-RT control reactions run in parallel.

Following heat inactivation, RT reactions were diluted 1/20 with nuclease free water, and triplicate reactions established for each biological replicate using Platinum™ SYBR™ Green qPCR SuperMix-UDG (with ROX). 25 µL reactions contained 12.5 µL mastermix, 2 µL diluted template and 0.4 µM Fw and Rv primer. Primers were Fw: 5’-TCCAGACCCTGTTGACCTTC-3’, Rv: 5’-TCGTCGAAGGAGAAGGACAG-3’ for the first NRPS gene in the discomycin BGC and Fw: 5’-CCACATTCATCGAGAGCAGC-3’, Rv: 5’-AAGAAGGCCCAGTCGATCAG-3’ for the *S. kanamyceticus* gyrA gene. Amplification was carried out using an Applied Biosystems StepOnePlus Real-Time PCR System using SYBR detection parameters with the following two step cycle parameters (95 °C,1 min, 40× {95 °C, 5 s; 60 °C, 30 s}) followed by a melt curve.

### Statistics & reproducibility

This study primarily describes the development and evaluation of a computational tool (DiscERN), together with supporting biological experiments that validated in‑silico predictions. No statistical method was used to predetermine sample size. Sample sizes for biological experiments (biological triplicates for minimum inhibitory concentration qPCR and cytotoxicity) reflect standard practice in the field, and computational benchmarks used the full datasets generated for each comparison unless otherwise stated.

For precision estimation, a maximum of 20 randomly sampled putative hits per confidence tier were manually curated, and hits that could not be confidently classified as true or false positives due to assembly fragmentation or boundary ambiguity (“not determined”) were excluded from the primary precision calculation; a conservative analysis treating all such cases as false positives is additionally reported (Fig. 3). No other data were excluded from the analyses.

Random selection was used in the computational benchmarks where indicated, including selection of 150 Actinomycetes genomes for specificity testing, sampling of hits for manual precision curation, and generation of simulated BGC fragments and concatemers. The biological experiments were not randomized. The investigators were not blinded to allocation during experiments and outcome assessment.

Statistical comparison of NRPS transcript levels between the *dscF*-overexpressing and empty-vector strains (Fig. 5d) was performed using a two-sided, unpaired Welch’s *t* test on ΔCt values (*n* = 3 biological replicates per condition). Ensemble performance was evaluated by leave-one-out cross-validation for recall and by manual curation of randomly sampled hits for precision, with F1 scores calculated as described in Methods. Computational analyses used fixed pipelines and parameters detailed in the Methods; the DiscERN source code and installation instructions are available at https://github.com/MaxMeta/DiscERN to enable reproduction.

### Reporting summary

Further information on research design is available in the Nature Portfolio Reporting Summary linked to this article.

## Supplementary information

Supplementary Information

Description of Additional Supplementary Files

Supplementary Data 1

Supplementary Data 2

Supplementary Data 4

Supplementary Data 5

Supplementary Data 6

Supplementary Data 7

Reporting Summary

Transparent Peer Review file

## Source data

Source data

## Data Availability

All genome sequences analysed in this study are publicly available from the NCBI Assembly database; the complete list of assembly accession numbers is provided in Supplementary Data 4. Reference biosynthetic gene clusters were obtained from the MIBiG database (v3.1) and the MIBiG identifiers used to define each antibiotic family are listed in Supplementary Data 1. Raw NMR supporting the structure elucidation of discomycin A have been deposited at Zenodo under 10.5281/zenodo.19692590. Source data are included with this paper. Supplementary Data 3 and 8–11 are available on Zenodo under https://zenodo.org/records/16791517 and https://zenodo.org/records/18667964, respectively. Supplementary Data 8 is a jupyter notebook containing parsing and analysis code used for benchmarking experiments. Supplementary Data 3, 9–11 are zipped folders containing antiSMASH outputs referred to in the manuscript. Source data are provided with this paper.

## Code availability

Instructions and source code for installation of DiscERN: https://github.com/MaxMeta/DiscERN^52^ The DOI for this release is: 10.5281/zenodo.19689229.
