# Supplementary material for: DiscERN: an automated genome mining tool for the discovery of evolutionarily related natural products
Source: Nat Commun. 2026 Jul 14;17:8657. doi: 10.1038/s41467-026-75491-x (PMC13490569; doi:10.1038/s41467-026-75491-x)
Supplement: Supplementary file 1 [file 41467_2026_75491_MOESM10_ESM.pdf]

nature portfolio

# Peer Review File

# DiscERN: An Automated Genome Mining Tool for the Discovery of Evolutionarily Related Natural Products

Corresponding Author: Dr Jeremy Owen

**This file contains all reviewer reports in order by version, followed by all author rebuttals in order by version.**

Version 0:

Reviewer comments:

Reviewer #1

(Remarks to the Author)
The manuscript by Owen et al. describes a new targeted genome mining approach to identify new members of gene cluster families of interest, and thus guide natural product discovery of prioritized compound classes. The principles used in the method are a combination of those from previously published algorithms, supplemented by matching of predicted monomers from antiSMASH. The tool comes with a nicely documented GitHub page and clear instructions for usage. The code is also well-documented and well-structured. While the tool itself does not appear conceptually/algorithmically groundbreaking, it does look practically quite useful.
A targeted genome mining approach like DiscERN is useful, and the approach chosen makes sense. Yet, the manuscript apparently lacks a comparison with other relevant similar targeted genome mining tools such as GATOR-GC, CORASON and cblaster, which are also not mentioned in the introduction, as well as the query mode of BiG-SCAPE.
It would also be useful to know how well the algorithm performs for BGCs other than those encoding NRPSs and PKSs, because e.g. RiPP and terpene BGCs can be quite small, which make them challenging to cluster by similarity.
The visualization of hierarchically clustered BGCs (in Figure 4) is very useful. For e.g. the glycopeptides, it would be very interesting to see a comparison of the clustering with published detailed phylogeny-based classifications of the glycopeptide BGC families (e.g., from the Ziemert and Wright labs).
The discovery of discomycin is a very nice practical showcase of how the DiscERN approach can be used and how it provides practical benefit to natural product researchers.

(Remarks on code availability)
The code is very nicely documented.
I was a bit puzzled by the choice of the AGPL license, as there is apparently no web service offered. An MIT, Apache or general GPL license would probably work well here. But of course this is the authors' own choice.

Reviewer #2

(Remarks to the Author)
Owen et al. present a robust and multi-modal bioinformatics tool that addresses the gap in targeted genome mining: the hypothesis-driven expansion of user-defined natural product families. The authors demonstrate that by integrating four complementary classification algorithms, including pfam domain similarity, sequence homology, and predicted product structure. The DiscERN provides a tunable strategy to balance discovery sensitivity with predictive precision. The tool's application led directly to the successful isolation and characterization of a novel antibiotic, discomycin A, from a silent biosynthetic gene cluster. This work bridges the gap between genomic data and natural product discovery, offering the community a accessible platform for prioritizing candidate BGCs. The ensemble approach, which smartly mitigates the inherent limitations of any single method, is a particular strength. Overall, this work deserves to be published after the authors address the following questions.

Major Concerns
1. In line 269, "The collection of BGCs identified as potentially encoding new bioactive compounds..."
What is the specific definition of "new (novelty)" in the context of this paper? Does it refer to significant structural differences

between the product of this BGC and known compounds, or does it indicate that the species or gene cluster background is being reported for the first time? How does the DiscERN model quantify "novelty"? Is it based on similarity to the MIBIG database (for example, is a value below a certain threshold considered new)? How was this threshold determined?

2. In the Figure 3, the model performance (e.g., Recall) for the CDA (calcium-dependent antibiotic) family was relatively weaker among the tested families.
Why was a BGC from the CDA family, which exhibited relatively weaker model performance, selected as the experimental validation example instead of opting for a family with near-perfect model performance, such as the glycopeptide family? What considerations influenced this decision? (For instance, does the CDA family hold greater potential for discovering novel compounds?) Does this introduce additional risks?

3. This question may appear to be an extension of Question 2: Why does the model performance for certain families (e.g., CDA) lag behind that of others? Is this due to higher inherent diversity within the family, or is it attributable to data quality issues? Such an analysis would be highly valuable for guiding future improvements to the model or its application to other families.

4. During the methodology development phase, the authors selected six specific natural product types for analysis in Figure 2. However, in the subsequent performance evaluation (Figure 3), polyenes and aminoglycosides were omitted. What is the reason for this?

5. The authors have developed a good BGC sorting pipeline and selected six specific natural product categories as the basis for model construction and testing. However, this selection appears to introduce a dependency on specific datasets. Could the authors clarify the rationale behind choosing these six particular types of natural products for building the models?

6. In natural product research, the precise delineation of BGC boundaries is a fundamental challenge. The authors' approach of fragmenting BGCs into linear subsequences reasonably demonstrates the model's sensitivity in classifying short DNA segments. However, as the model's input relies heavily on data from tools like antiSMASH or databases like MIBIG, which themselves often suffer from imprecise BGC boundary predictions. Therefore, a critical question arises, Does the model developed in this study address the inherent challenge of BGC boundary prediction, and to what extent are its results affected by inaccuracies in the boundary definitions of the input data?

Minor Concerns
1. In line 109, based on the context, "CDA" appears to refer to calcium ion-dependent antibiotics. Given that this is its first occurrence in the text, the authors should provide the full term rather than an abbreviation.

2. The presentation of Figure 1 is currently unacceptable. The current diagram portrays "Pfam similarity," "Sequence similarity," and "Structure similarity" as three parallel inputs, which may mislead readers into thinking that users need to precompute these features. Actually, the main text clarifies that these similarity calculations are core components of DiscERN's internal algorithms (corresponding to the pfam-vec, blast-vec/rank, and k-mer methods, respectively). It is recommended to relocate the three icons in section (c) into the blue box representing DiscERN and use arrows, lists, or other visual cues to indicate that they are integrated analytical modules within the model.

3. The authors need to provide more scientific evidence to demonstrate that SARP overexpression is directly correlated with the activation of the discomycin A BGC, such as through RT-PCR. Additionally, further literature support should be provided for the related claim that "a SARP gene that suggested a facile means for activation."

(Remarks on code availability)

Version 1:

Reviewer comments:

Reviewer #1

(Remarks to the Author)
I am glad to see the authors addressed all my points well.

I just have two minor grammatical points related to the new paragraph in the Introduction:
- line 92: "clustering tools like and..." -> Remove 'and'
- line 95: "To address this challenge" -> This now no longer refers to the paragraph directly preceding it, so I suggest adjusting the language a bit.

(Remarks on code availability)
No changes have been made since last time.

Reviewer #2

(Remarks to the Author)

I would like to emphasize that the authors have replied the majority of our comments. A few additional concerns remain, however, that should be addressed prior to final acceptance. These are detailed below,

Major Concerns
1. DiscERN integrates four similarity-based classifiers (Pfam vector, BLAST vector, BLAST rank, and structural k-mer). However, these approaches appear to rely largely on existing similarity metrics rather than introducing a fundamentally new computational framework. Could the authors clarify what methodological innovation DiscERN provides beyond the integration of these existing strategies?

2. DiscERN combines several existing similarity-based approaches (Pfam vectorization, BLAST similarity, rank-based homology, and structural k-mer comparison). Could the authors clarify what methodological advance DiscERN provides beyond integrating these existing strategies into a unified workflow? In particular, did the authors consider more recent representation learning approaches for BGC similarity (e.g., protein language models or learned embeddings), and how would DiscERN compare with such approaches?

3. The benchmarking relies heavily on manual annotation of predicted hits to define true positives and false positives. Could the authors clarify how reproducible this process is and whether objective criteria or independent validation were used?

Minor Concerns
Some of the figure captions appear inconsistent. The author should carefully review these sections.
For example, the label for Figure 4e at line 376, and the label for Figure 4c at line 383, as well as other similar instances elsewhere.

(Remarks on code availability)

Version 2:

Reviewer comments:

Reviewer #2

(Remarks to the Author)
The authors have answered my questions, and this manuscript can be published.

(Remarks on code availability)

**Open Access** This Peer Review File is licensed under a Creative Commons Attribution 4.0 International License, which permits use, sharing, adaptation, distribution and reproduction in any medium or format, as long as you give appropriate credit to the original author(s) and the source, provide a link to the Creative Commons license, and indicate if changes were made.

In cases where reviewers are anonymous, credit should be given to 'Anonymous Referee' and the source.
The images or other third party material in this Peer Review File are included in the article's Creative Commons license, unless indicated otherwise in a credit line to the material. If material is not included in the article's Creative Commons license and your intended use is not permitted by statutory regulation or exceeds the permitted use, you will need to obtain permission directly from the copyright holder.
To view a copy of this license, visit https://creativecommons.org/licenses/by/4.0/

Dear Editor and Reviewers,

Thank you for your careful reading of the manuscript and for the constructive comments and suggestions. We have revised the manuscript accordingly. Below, we provide a point-by-point response to each comment. Reviewer text is reproduced in black, followed by our response and a description of the corresponding changes in the revised manuscript in blue.

Sincerely,

Jeremy Owen

**Response to reviewer 1:**

1) Yet, the manuscript apparently lacks a comparison with other relevant similar targeted genome mining tools such as GATOR-GC, CORASON and cblaster, which are also not mentioned in the introduction, as well as the query mode of BiG-SCAPE.

   We have revised the Introduction to include GATOR-GC, CORASON, cblaster, and BiG-SCAPE, and to distinguish their functionality from that of DiscERN. We also added benchmarking experiments against GATOR-GC and the latest BiG-SCAPE release, as these are the closest comparators for the specific classification and clustering tasks evaluated here. The benchmarking analyses required custom downstream processing. All scripts, raw datafiles, and Jupyter notebooks used to generate the revised figures and tables are now available as supplementary files and/or archived on Zenodo. A brief summary of the benchmarking outcomes is included in the main text, with full methods and performance statistics provided in the revised Supplementary Materials.

2) It would also be useful to know how well the algorithm performs for BGCs other than those encoding NRPSs and PKSs, because e.g. RiPP and terpene BGCs can be quite small, which make them challenging to cluster by similarity.

   We have extended the evaluation to the thiostrepton-like RiPP family. This family was selected because it contains a conserved, but variable, core peptide motif that can be identified independently from biosynthetic context, providing an external check on classification accuracy. Importantly, thiostrepton-like RiPPs were not used during model development. DiscERN performed well on this task, supporting its applicability beyond NRPS/PKS containing BGCs. This analysis is now described in the main text, and the underlying data have been uploaded to Zenodo as an additional datafile that is referenced in the main text.

3) The visualization of hierarchically clustered BGCs (in Figure 4) is very useful. For e.g. the glycopeptides, it would be very interesting to see a comparison of the clustering with published detailed phylogeny-based classifications of the glycopeptide BGC families (e.g., from the Ziemert and Wright labs).

   We have now compared our hierarchical clustering results with published glycopeptide antibiotic (GPA) classifications based on structure and BGC features.

Overall, DiscERN's hierarchical clustering recapitulates the established GPA groups (I–V) and is broadly consistent with prior phylogeny-based analyses, including approaches using A-domain specificity features. We have added a concise description of this comparison to the main text.

**Response to reviewer 1:**

1) In line 269, "The collection of BGCs identified as potentially encoding new bioactive compounds..." What is the specific definition of "new (novelty)" … How was this threshold determined?

   We opted for a conservative definition of novelty that likely underestimates the proportion of identified BGCs that produce previously uncharacterised compounds. Specifically, for the purpose of determining the proportion of BGCs which were "novel" we restricted our analysis to NRPS/PKS BGCs and a BGC was labelled as potentially producing a new member of its targeted family if the substrate specificities and/or order of substrate incorporation differed from any other member within that family of natural products. This point has been clarified in the revised manuscript text and supplementary methods.

2) In the Figure 3, the model performance (e.g., Recall) for the CDA (calcium-dependent antibiotic) family was relatively weaker among the tested families. Why was a BGC from the CDA family, which exhibited relatively weaker model performance, selected as the experimental validation example instead of opting for a family with near-perfect model performance, such as the glycopeptide family? What considerations influenced this decision? (For instance, does the CDA family hold greater potential for discovering novel compounds?) Does this introduce additional risks?

   We selected the discomycin BGC as a proof-of-concept for several practical and scientific reasons: (i) the model predicted clear substrate specificity differences consistent with a new analogue; (ii) the presence of a SARP regulator suggested a plausible activation strategy for what appeared to be a silent cluster in a well characterised strain; (iii) the strain was readily available (DSMZ) and compatible with existing import permits; and (iv) we have prior experience with isolation and structure elucidation workflows for this compound class, reducing experimental risk. Although CDA performance is lower than some other families, this made it an informative test case for validating model utility on a challenging, diverse family.

3) This question may appear to be an extension of Question 2: Why does the model performance for certain families (e.g., CDA) lag behind that of others? Is this due to higher inherent diversity within the family, or is it attributable to data quality issues? Such an analysis would be highly valuable for guiding future improvements to the model or its application to other families.

   While we cannot definitively separate these effects, we consider BGC diversity within the CDAs to be a major contributor. CDA-like antibiotics occur across diverse

actinomycete taxa and show substantial variation in both BGC sequence and product structure despite shared functional activity. In addition, CDA-like BGCs appear to lack a small set of consistently co-occurring, family-specific biosynthetic markers that are rare outside the family, which likely reduces discriminative signal relative to more conserved families. These properties make CDA a stringent test for model generalisation.

4) During the methodology development phase, the authors selected six specific natural product types for analysis in Figure 2. However, in the subsequent performance evaluation (Figure 3), polyenes and aminoglycosides were omitted. What is the reason for this?

We included these families in the initial model development and benchmarking experiments (i.e. LOO analysis, specificity and fragment/concatenation robustness) but opted to exclude them from the large-scale application and evaluation of the tool owing to the large burden of required manual curation of outputs they would have entailed.

5) The authors have developed a good BGC sorting pipeline and selected six specific natural product categories as the basis for model construction and testing. However, this selection appears to introduce a dependency on specific datasets. Could the authors clarify the rationale behind choosing these six particular types of natural products for building the models?

We agree that model development and evaluation can become dependent on the availability and quality of curated reference datasets. We selected these families to balance (i) biosynthetic diversity (ii) sufficient numbers of characterised representatives in MIBiG to enable training and manual validation, and (iii) tractable expert validation, given that reliable performance assessment requires domain-specific review of predicted hits. To address generalisability beyond the initial set, we have now extended the analysis to an additional and biosynthetically distinct family (thiostrepton-like RiPPs), where DiscERN also performed well. Together, these results support DiscERN as a broadly applicable approach rather than one restricted to a particular dataset

6) In natural product research, the precise delineation of BGC boundaries is a fundamental challenge. The authors' approach of fragmenting BGCs into linear subsequences reasonably demonstrates the model's sensitivity in classifying short DNA segments. However, as the model's input relies heavily on data from tools like antiSMASH or databases like MIBIG, which themselves often suffer from imprecise BGC boundary predictions. Therefore, a critical question arises, Does the model developed in this study address the inherent challenge of BGC boundary prediction, and to what extent are its results affected by inaccuracies in the boundary definitions of the input data?

We agree that BGC boundary definition is a fundamental challenge and that inputs derived from antiSMASH/MIBiG may include boundary inaccuracies. DiscERN does

not explicitly solve boundary prediction, instead, we assessed robustness to boundary artefacts by simulating increasingly severe overextension through random concatemer experiments. Specifically, an experimentally characterised family member BGC was concatenated with 1–4 randomly selected non-family BGCs, and we evaluated whether trained models still correctly identified the family signal within these chimeric inputs (100 concatemers per concatemer length across multiple reference BGCs). As expected, accuracy decreased as the amount of extraneous sequence increased, but performance remained relatively robust when the added sequence was modest.

Minor Concerns

1) In line 109, based on the context, "CDA" appears to refer to calcium ion-dependent antibiotics. Given that this is its first occurrence in the text, the authors should provide the full term rather than an abbreviation.

   We have fixed this in the revised manuscript.

2) The presentation of Figure 1 is currently unacceptable. The current diagram portrays "Pfam similarity," "Sequence similarity," and "Structure similarity" as three parallel inputs, which may mislead readers into thinking that users need to precompute these features. Actually, the main text clarifies that these similarity calculations are core components of DiscERN's internal algorithms (corresponding to the pfam-vec, blast-vec/rank, and k-mer methods, respectively). It is recommended to relocate the three icons in section (c) into the blue box representing DiscERN and use arrows, lists, or other visual cues to indicate that they are integrated analytical modules within the model.

   We have revised Figure 1 so that Pfam-, sequence-, and k-mer–based similarity calculations are presented as internal DiscERN modules, rather than inputs that users must compute externally.

3) The authors need to provide more scientific evidence to demonstrate that SARP overexpression is directly correlated with the activation of the discomycin A BGC, such as through RT-PCR. Additionally, further literature support should be provided for the related claim that "a SARP gene that suggested a facile means for activation."

   We have added RT-qPCR data showing that *dscF* overexpression increases transcription of the first NRPS gene in the discomycin BGC by ~120-fold relative to the empty-vector control. We added citation for a recent review of the role of SARPs in regulation of secondary metabolism that supports the role of SARP-family regulators as pathway-specific activators of secondary metabolite BGCs. We have also replaced ‘facile” with ‘plausible’ in the quoted text to avoid making an overly strong assertion.

Dear Editor and Reviewers,

Thank you for your careful reading of the revised manuscript and for the additional constructive comments and suggestions. We have revised the manuscript accordingly. Below, we provide a point-by-point response to each comment. Reviewer text is reproduced in italics, followed by our response and a description of any corresponding changes in the revised manuscript and accompanying files.

**Reviewer 1**

*I just have two minor grammatical points related to the new paragraph in the Introduction:*

We thank the reviewer for identifying these grammatical errors, We have corrected them in the revised version of the manuscript.

**Reviewer 2**

*DiscERN integrates four similarity-based classifiers (Pfam vector, BLAST vector, BLAST rank, and structural k-mer). However, these approaches appear to rely largely on existing similarity metrics rather than introducing a fundamentally new computational framework. Could the authors clarify what methodological innovation DiscERN provides beyond the integration of these existing strategies?*

We thank the reviewer for this important point. We agree that several components of DiscERN build on established similarity concepts, and we do not wish to overstate novelty at the level of every individual metric. However, DiscERN is not simply an ad hoc aggregation of existing approaches. Its methodological contribution lies in the development of a dedicated, automated framework for targeted multi-reference BGC family expansion, a task that previously required bespoke combinations of tools, manual collation of outputs, and subjective prioritisation of candidate hits.

More specifically, DiscERN integrates orthogonal representations of relatedness at the levels of biosynthetic domain content, direct sequence similarity, rank-based homology, and predicted product architecture; combines these into an interpretable ensemble framework with tuneable confidence thresholds; applies automated family-specific filtering based on conserved Pfam content; and returns prioritisation-ready outputs for downstream validation. In addition, for NRPS/PKS families, DiscERN introduces structural k-mer intersection, a new predicted-product similarity metric based on local k-mer content derived from antiSMASH monomer predictions. This provides a product-level representation that is less dependent on strict global collinearity than direct sequence comparison alone.

*…did the authors consider more recent representation learning approaches for BGC similarity (e.g., protein language models or learned embeddings), and how would DiscERN compare with such approaches?*

We thank the reviewer for this thoughtful suggestion. We agree that learned embedding approaches are an interesting potential direction for future work, and that such methods may ultimately prove useful for some BGC detection or classification tasks. However, a learned embedding is not a universal representation, but one shaped by the objective used to train it. For targeted BGC family expansion, the relevant objective would be to retrieve clusters

belonging to the same family, or encoding closely related chemistry, from one or a few reference examples.

At present, this is not a setting in which a task-specific embedding approach can be rigorously developed and fairly evaluated. As noted in the manuscript, there is currently no complete gold-standard dataset for this task with well-defined true positives, true negatives, and false negatives, and the number of characterized BGCs remains too limited to support conventional train/test partitioning. A learned embedding method specifically optimised for targeted BGC family assignment would therefore require substantially larger volumes of high-quality labelled training data than are currently available in the field.

We therefore considered an interpretable, training-light framework to be the more appropriate methodological choice for the present objective. DiscERN is designed to operate directly on small, user-defined reference sets, standard antiSMASH outputs, and explicit similarity signals that can be readily interpreted by users.

*The benchmarking relies heavily on manual annotation of predicted hits to define true positives and false positives. Could the authors clarify how reproducible this process is and whether objective criteria or independent validation were used?*

We agree that the manual annotation procedure should be described explicitly. In the absence of an independent gold-standard benchmark for targeted BGC family expansion, manual curation and consensus by experts was deemed to be the only available option. We have updated the supplementary materials to include a more detailed description of manual classification criteria is provided supplementary methods to clarify the criteria used for assigning hits as true positives, false positives, and unresolved or indeterminate cases. **Please refer to section S3, subheading "Precision Estimation and Handling of Undetermined Cases".**

To improve transparency and reproducibility, and allow independent verification by the research community, we have also ensured that the relevant manual classification inputs, parsing steps, and downstream analysis code are available and clearly referenced in the manuscript. In particular, we refer the reviewer to the following paired supplementary data files that contain *class assignments → annotated BGC data* mappings: 2→3, 5→10, 6→11.

*Some of the figure captions appear inconsistent. The author should carefully review these sections. For example, the label for Figure 4e at line 376, and the label for Figure 4c at line 383, as well as other similar instances elsewhere.*

We thank the reviewer for noting these inconsistencies. We have carefully re-checked the figure captions and in-text panel references throughout the manuscript and corrected the mislabelled figure callouts.
