# Supplementary material for: DiscERN: an automated genome mining tool for the discovery of evolutionarily related natural products
Source: Nat Commun. 2026 Jul 14;17:8657. doi: 10.1038/s41467-026-75491-x (PMC13490569; doi:10.1038/s41467-026-75491-x)
Supplement: Supplementary file 2 [file 41467_2026_75491_MOESM1_ESM.pdf]

Supplementary Information for

DiscERN: An Automated Genome Mining Tool for the Discovery of Evolutionarily Related Natural Products

Jeremy G. Owen*, Ethan F. Woolly, Hung-En Lai, V. Helen Woolner and Rory F. Little

Table of Contents:

Supplementary Figures and Tables: **2**

Supplementary Table 1 – Specificity Analysis Details 2

Supplementary Figure 1. Comparison of the Discomycin and CDA BGCs 3

Supplementary Table 2. Putative Functions of Genes in the Discomycin BGC 3

Supplementary Figure 2. HRESI-MS Spectrum of Discomycin A 3

Supplementary Figure 3. Atom Numbering for Discomycin A 4

Supplementary Table 3: Tabulated NMR Data for Discomycin A 4

Supplementary Figure 4. Key Correlations Defining Amino Acid Identity in Discomycin A 5

Supplementary Figure 5. Key Correlations Defining Amino Acid Connectivity in Discomycin A 5

Supplementary Figure 6. HRESI-MS/MS Fragmentation of Discomycin A 6

Supplementary Table 4 – Summary of Marfey's Analysis for Discomycin A 6

Supplementary Figure 7: Marfey's analysis of discomycin A. 7

Figures show extracted ion chromatograms from LC-MS analysis L-and D-FDAA derivatives of the hydrolysate of discomycin and amino acid standards. Mass range selected for each amino acid derivative is indicated in the corresponding panel label 7

Supplementary Figure 8. 1H spectrum of discomycin A 10

Supplementary Figure 9. 1H-1H COSY Spectrum of discomycin A 11

Supplementary Figure 10. 13C spectrum of discomycin A 12

Supplementary Figure 11. 1H-13C HSQC spectrum of discomycin A 13

Supplementary Figure 12. 1H-1H ROESY spectrum of discomycin A 14

Supplementary Figure 13. 1H-13C HMBC spectrum of discomycin A 15

Supplementary Table 5. MIC values for discomycin A 16

Supplementary Figure 14. DiscERN Output Dendrogram Showing Relatedness of Putative Hits 16

A) Macrolides 16
B) Glycopeptides 17
C) CDAs 18
D) Rifamycin Like 19

Supplementary Table 6. Classification Performance for BiG-SCAPE at Various Clustering Thresholds: 19

Supplementary Table 7. Classification Performance for GATOR-GC at Various Gator Focal Score Thresholds: 20

Supplementary Table 8. qPCR Ct values for all biological and technical replicates: 20

Supplementary Figure 15. Comparison of new core sequences from thiostreoton-like RiPP BGCs identified by DiscERN to characterised thiostrepton-like core sequences 21

Supplementary References: **21**

Supplementary Figures and Tables:

Supplementary Table 1 – Specificity Analysis Details

Polymer	TP	FP	false positives per 1000 BGCs
CDA	0	1	0.38
polyene	7	0	0.00
glycopeptides	6	0	0.00
rif like	0	0	0.00
amino glycos	N/A	N/A	N/A
macrolides	0	0	0.00
**BS**	**TP**	**FP**	
CDA	0	0	0.00
polyene	1	0	0.00
glycopeptides	5	1	0.38
rif like	0	0	0.00
amino glycos	1	0	0.00
macrolides	0	0	0.00
**OL**	**TP**	**FP**	
CDA	3	0	0.00
polyene	8	0	0.00
glycopeptides	6	0	0.00
rif like	0	0	0.00
amino glycos	2	8	3.06
macrolides	0	0	0.00
**BV**	**TP**	**FP**	
CDA	3	9	3.44
polyene	10	1	0.38
glycopeptides	6	6	2.30
rif like	0	0	0.00
amino glycos	2	35	13.39
macrolides	0	0	0.00

Supplementary Figure 1. Comparison of the Discomycin and CDA BGCs

Outputs from the "known cluster-blast" function of antiSMASH are presented. Genes predicted to have the same function have the same colour. Letters in the Dsc cluster match the table below.

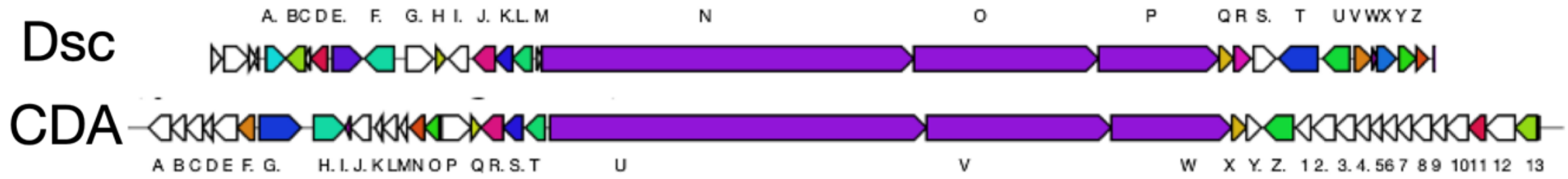

Supplementary Table 2. Putative Functions of Genes in the Discomycin BGC

Gene	Protein Type	Putative Function
**A**	prephenate dehydrogenase	Unknown
**B**	beta-ketoacyl-ACP synthase family protein	Fatty acid biosynthesis
**C**	acyl carrier protein	Fatty acid biosynthesis
**D**	ketoacyl-ACP synthase III	Fatty acid biosynthesis
**E**	fatty acyl-AMP ligase	Fatty acid biosynthesis
**F**	Streptomyces Antibiotic Regulatory Protein (SARP)	Regulatory
**G**	Histidine kinase	Regulatory
**H**	response regulator transcription factor	Regulatory
**I**	IS110 family transposase	N/A
**J**	PLP-dependent aminotransferase	HPG biosynthesis
**K**	alpha-hydroxy acid oxidase	HPG biosynthesis
**L**	4-hydroxyphenylpyruvate dioxygenase	HPG biosynthesis
**M**	acyl carrier protein	Core Biosynthesis
**N**	non-ribosomal peptide synthetase	Core Biosynthesis
**O**	non-ribosomal peptide synthetase	Core Biosynthesis
**P**	non-ribosomal peptide synthetase	Core Biosynthesis
**Q**	Alpha-beta hydrolase	Type-II Thioesterase
**R**	LLM class flavin-dependent oxidoreductase	aa or fatty acid oxidation
**S**	M20/M25/M40 family metallo-hydrolase	Unknown
**Y**	HAD-IC family P-type ATPase	Transport/export
**U**	ABC transporter ATP-binding protein	Transport/export
**V**	class I SAM-dependent methyltransferase	Glutamine Methylation
**W**	MbtH family protein	Core Biosynthesis
**X**	cytochrome P450	aa or fatty acid oxidation
**Y**	ABC transporter ATP-binding protein	Transport/export
**Z**	ABC transporter	Transport/export

Supplementary Figure 2. HRESI-MS Spectrum of Discomycin A

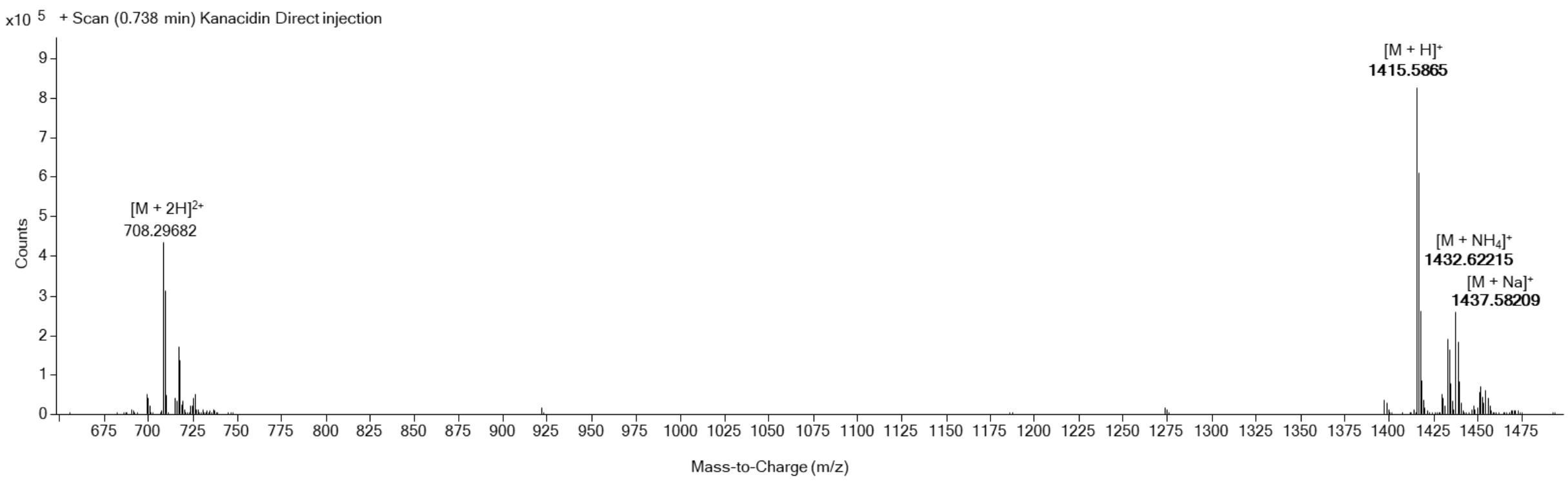

Supplementary Figure 3. Atom Numbering for Discomycin A

Numbers match descriptions in the main text and supplementary materials.

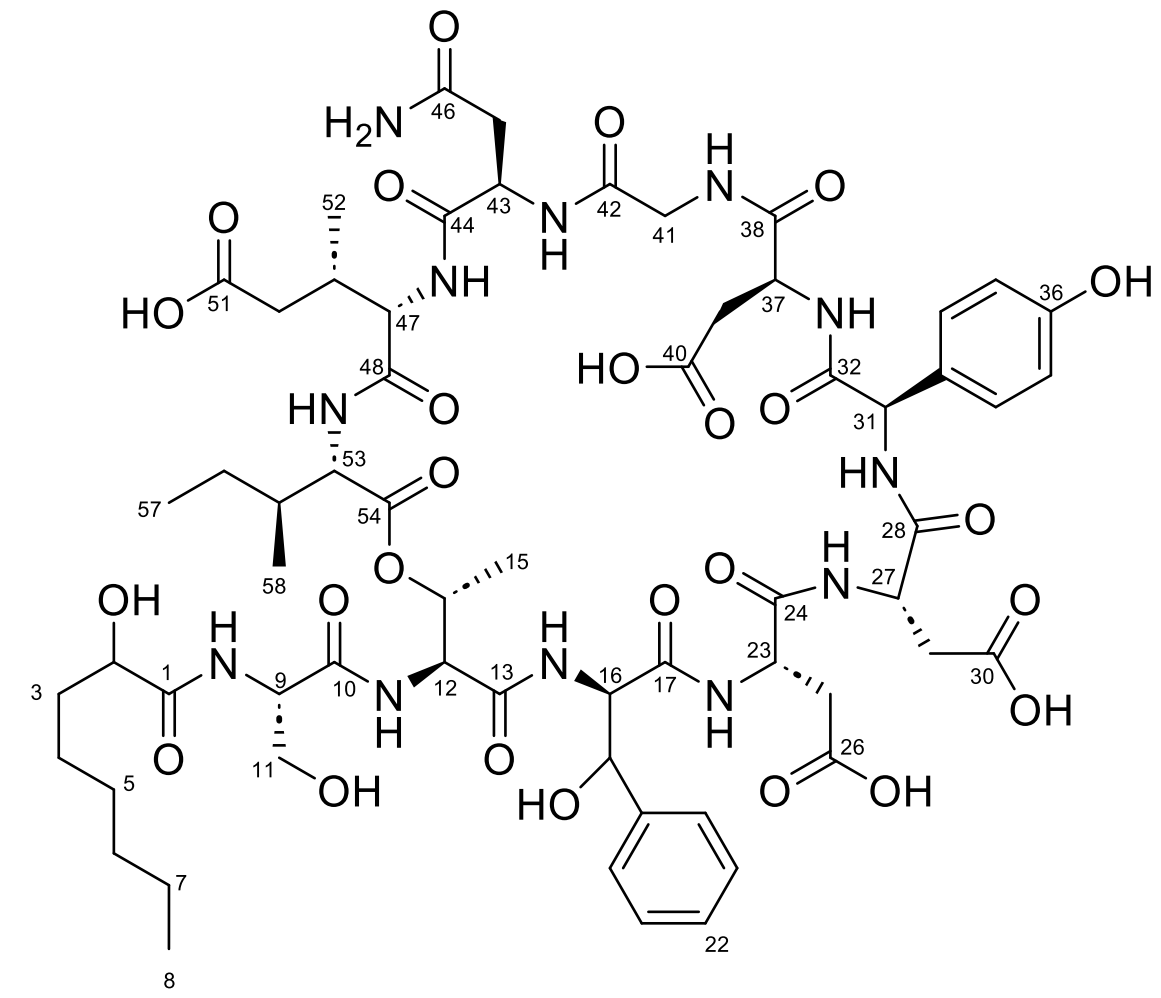

Supplementary Table 3: Tabulated NMR Data for Discomycin A

Pos.	δ_C, type	δ_H, mult. (J in Hz)
	**2-hydroxyoctanoic acid**	
1	177.4, C	-
2	72.9, CH	4.07, dd (8.0, 3.5)
3	35.6, CH_2	1.77
		1.61
4	26.1, CH_2	1.45
		1.43
5	30.2, CH_2	1.30
6	32.9, CH_2	1.30
7	23.7, CH_2	1.31
8	14.4, CH_3	0.89
	**Serine-2**	
9	55.5, CH	4.65
10	172.5, C	-
11	63.2, CH_2	3.88, dd, 11, 5.8
		3.78, dd, 10.7, 6.2
	**Threonine-3**	
12	57.7, CH	4.62, d (4.3)
13	171.1, C	-
14	71.0, CH	5.26, br s
15	16.2, CH_3	1.07
	**B-Hydroxyl phenylalanine-4**	
16	61.4, CH	4.66
17	172.5, C	-
18	73.8, CH	5.37, br s
19	142.0, C	-
20	127.4, CH	7.38, d (7.0)
21	129.3, CH	7.31, t (7.0)
22	128.7, CH	7.25
	**Aspartic Acid-5**	
23	51.6, CH	4.79
24	174.3, C	-
25	36.4, CH_2	2.79
		2.76
26	173.2, C	-
	**Aspartic Acid-6**	
27	51.5, CH	4.83
28	173.2, C	-
29	36.8, CH2	2.93
		2.84
30	174.5, C	-

Supplementary Figure 4. Key Correlations Defining Amino Acid Identity in Discomycin A

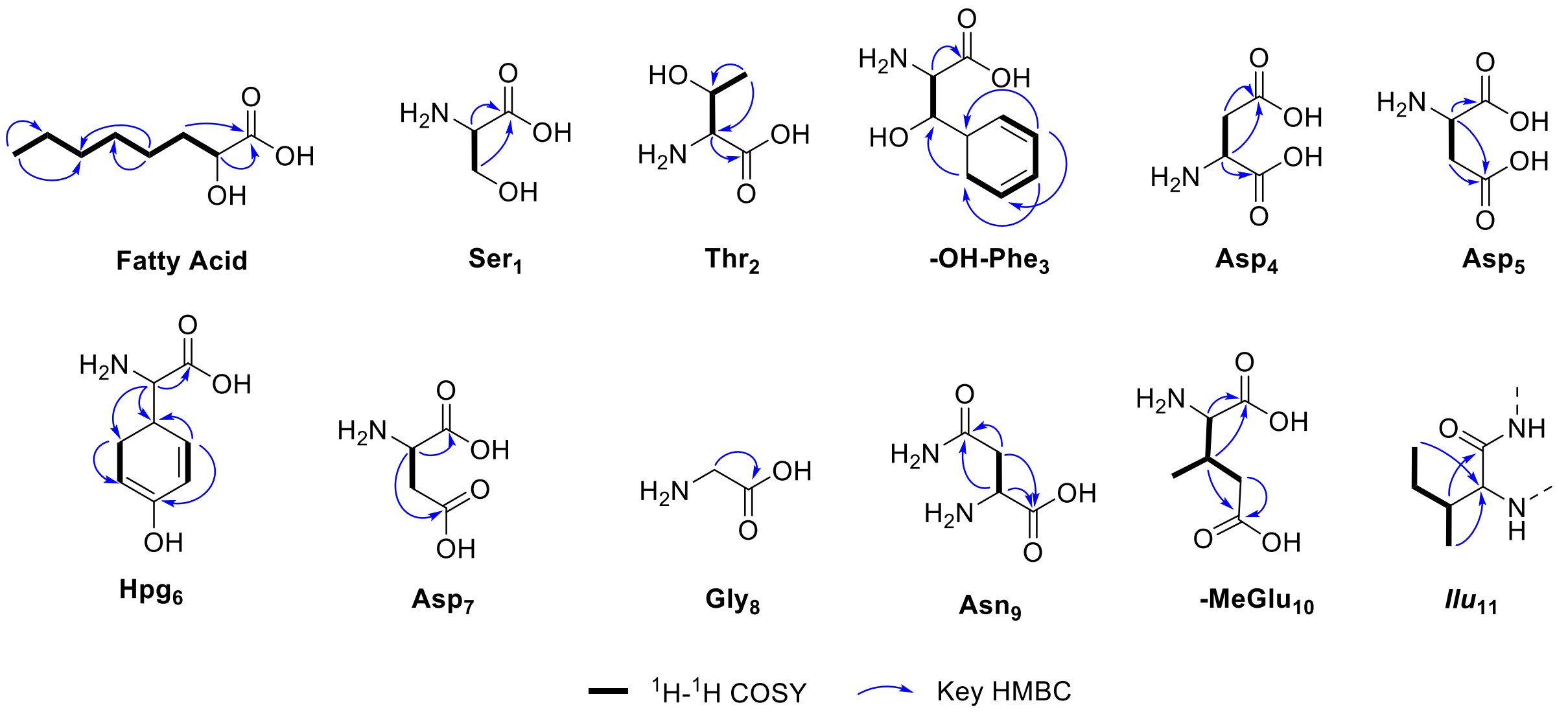

Supplementary Figure 5. Key Correlations Defining Amino Acid Connectivity in Discomycin A

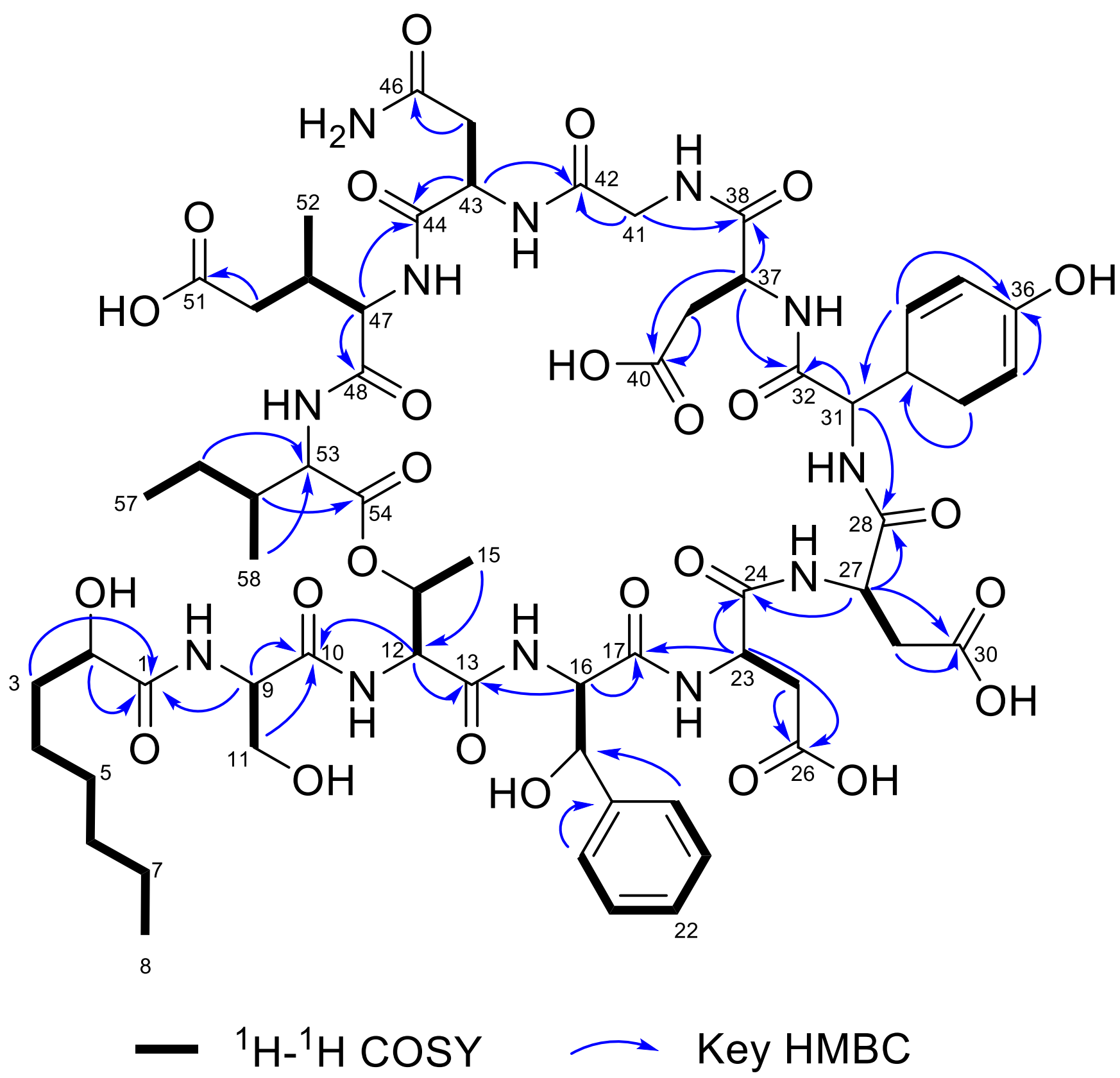

Supplementary Figure 6. HRESI-MS/MS Fragmentation of Discomycin A

Structures for B-ions (red labels) and Y-ions (blue labels) are indicated in the linear structure presented in the lower panel

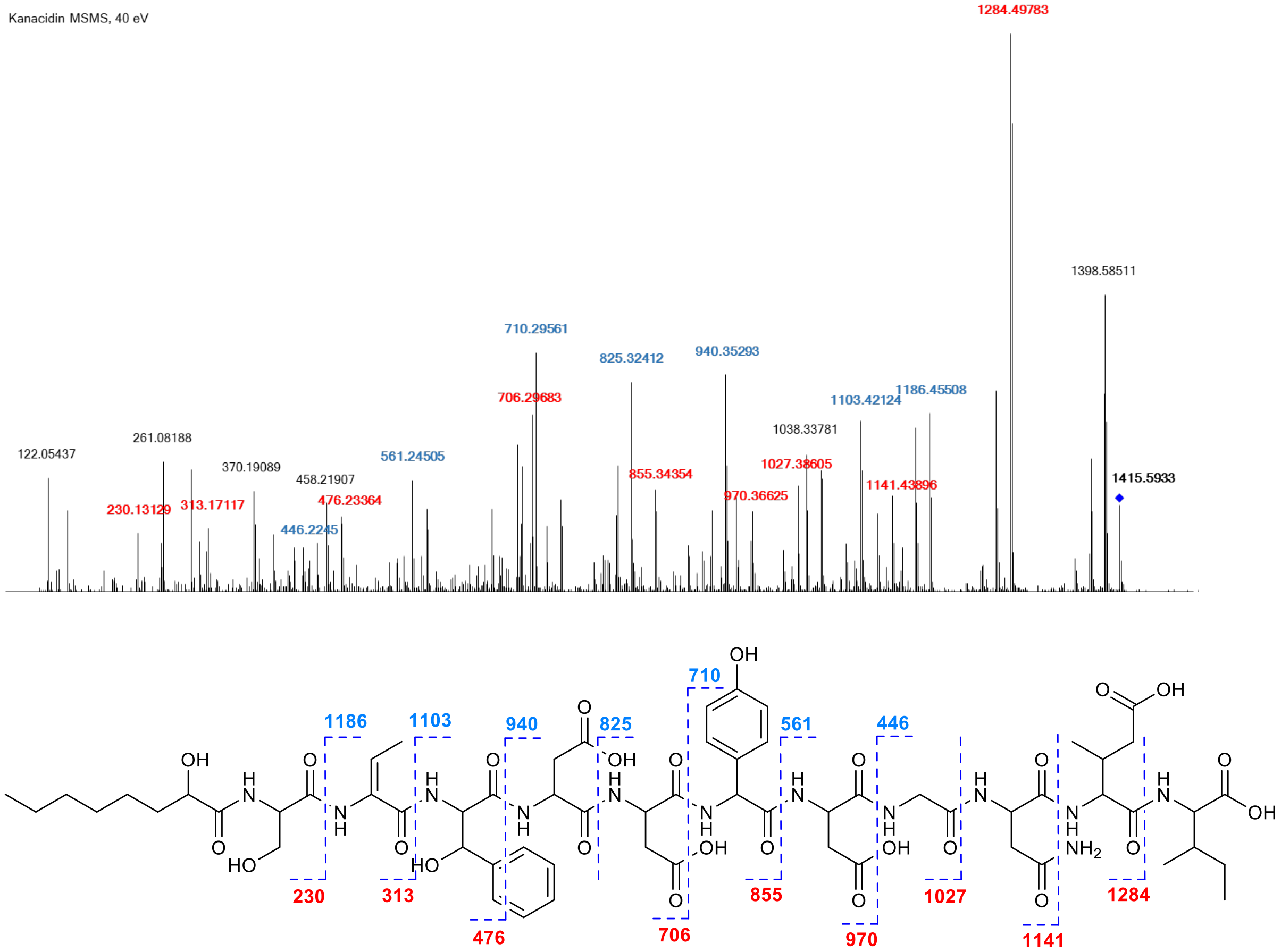

Supplementary Table 4 – Summary of Marfey's Analysis for Discomycin A

Amino Acid	Mass of AA + FDAA	Known Elution Order	Amino Acid standards		Hydrolysate of discomycin		L or D
			tR L-L or D-D	tR L-D or D-L	tR (AA-L-FDAA)	tR (AA-D-FDAA)	
Ser	358.0993	L→D[1]	17.56	18.50	17.48	18.22	L
Thr	372.1150	L→D[1]	19.00	25.70	18.89	25.57	L
Allo-Thr	372.1150	L→D[1]	20.07	22.56	-	-	-
β-OH-Phe	434.1306	L→D[2]	-	-	37.88	30.58	D
Asp	386.0943	L→D[1]	22.08	25.40	22.32	25.4	L
Hpg	420.1150	L→D[3]	14.71	14.84	-	-	-
Hpg Dimer	672.1645	-	53.23	62.63	62.59	53.15	D
Gly	327.1150	-	22.67	22.57	22.65	22.52	-
Asn	385.1102	L→D[1]	17.28	18.15	-	-	D*
β-Me-Glu	414.1256	L→D[4]	-	-	27.225	31.33	2S, 3S
Ile	384.1514	L→D[1]	39.53	48.05	39.88	48.44	L
Allo- Ile	384.1514	L→D[1]	38.39	47.06	-	-	-

[1]https://pubs.acs.org/doi/10.1021/acs.jnatprod.5b01125
[2]https://pubs.acs.org/doi/10.1021/acschembio.0c00663

Supplementary Figure 7: Marfey's analysis of discomycin A.

Figures show extracted ion chromatograms from LC-MS analysis L-and D-FDAA derivatives of the hydrolysate of discomycin and amino acid standards. Mass range selected for each amino acid derivative is indicated in the corresponding panel label

A) Ser, *m/z* = 357.5993 – 358.5993

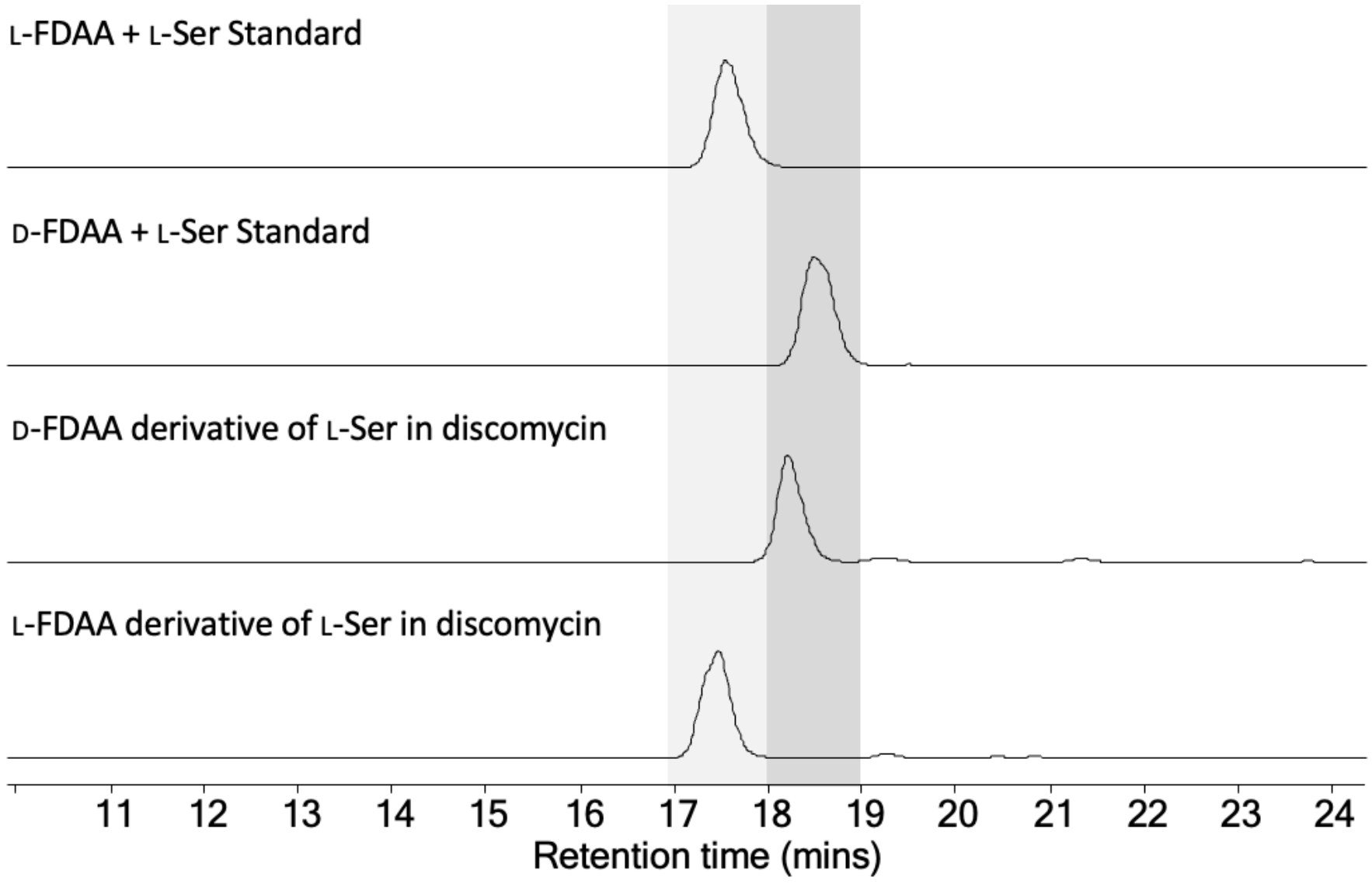

B) Thr, *m/z* = 371.615 – 372.625

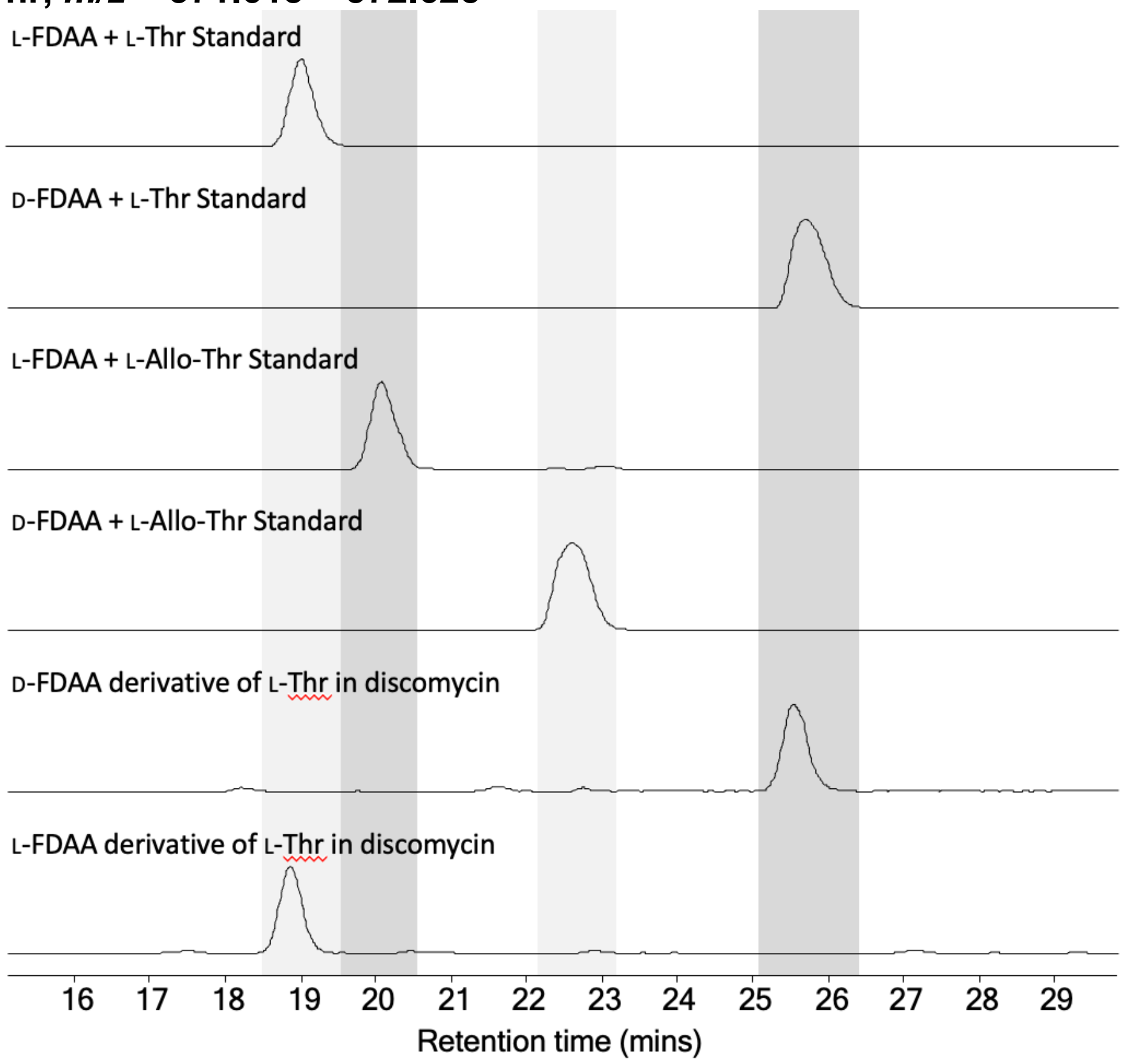

C) Asp, *m/z* = 385.5943 – 386.5943

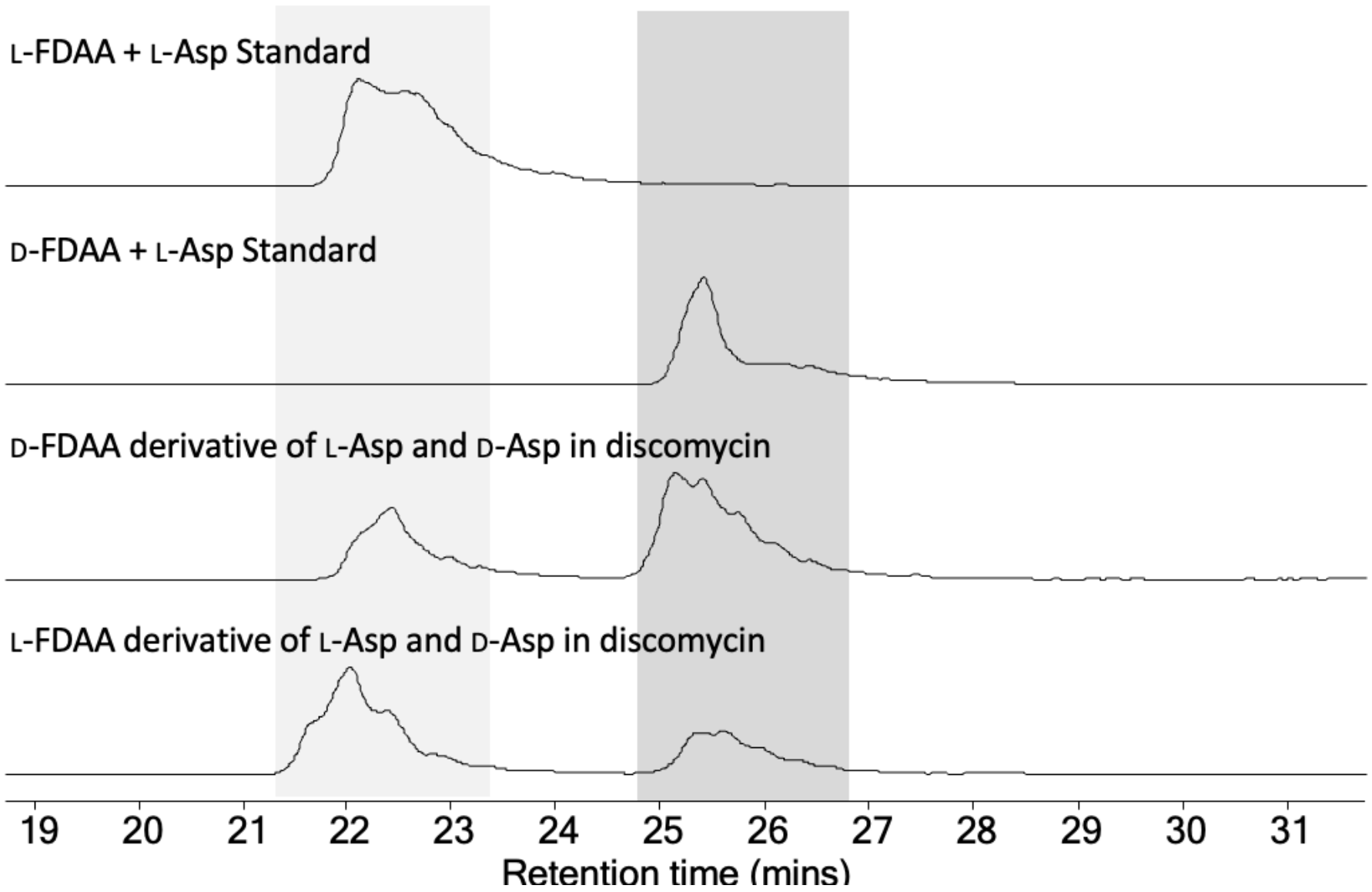

D) β-OH-Phe, *m/z* = 433.6306 – 434.6306

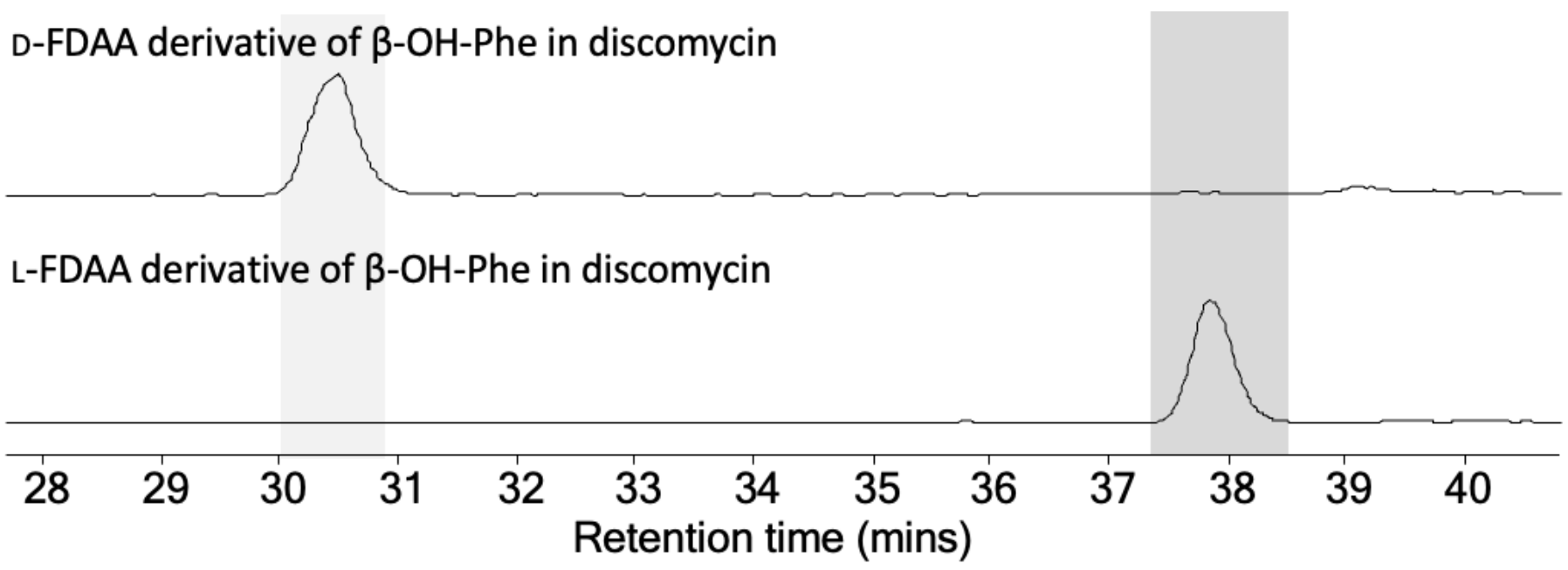

E) Hpg, *m/z* = 671.6645 – 672.6645

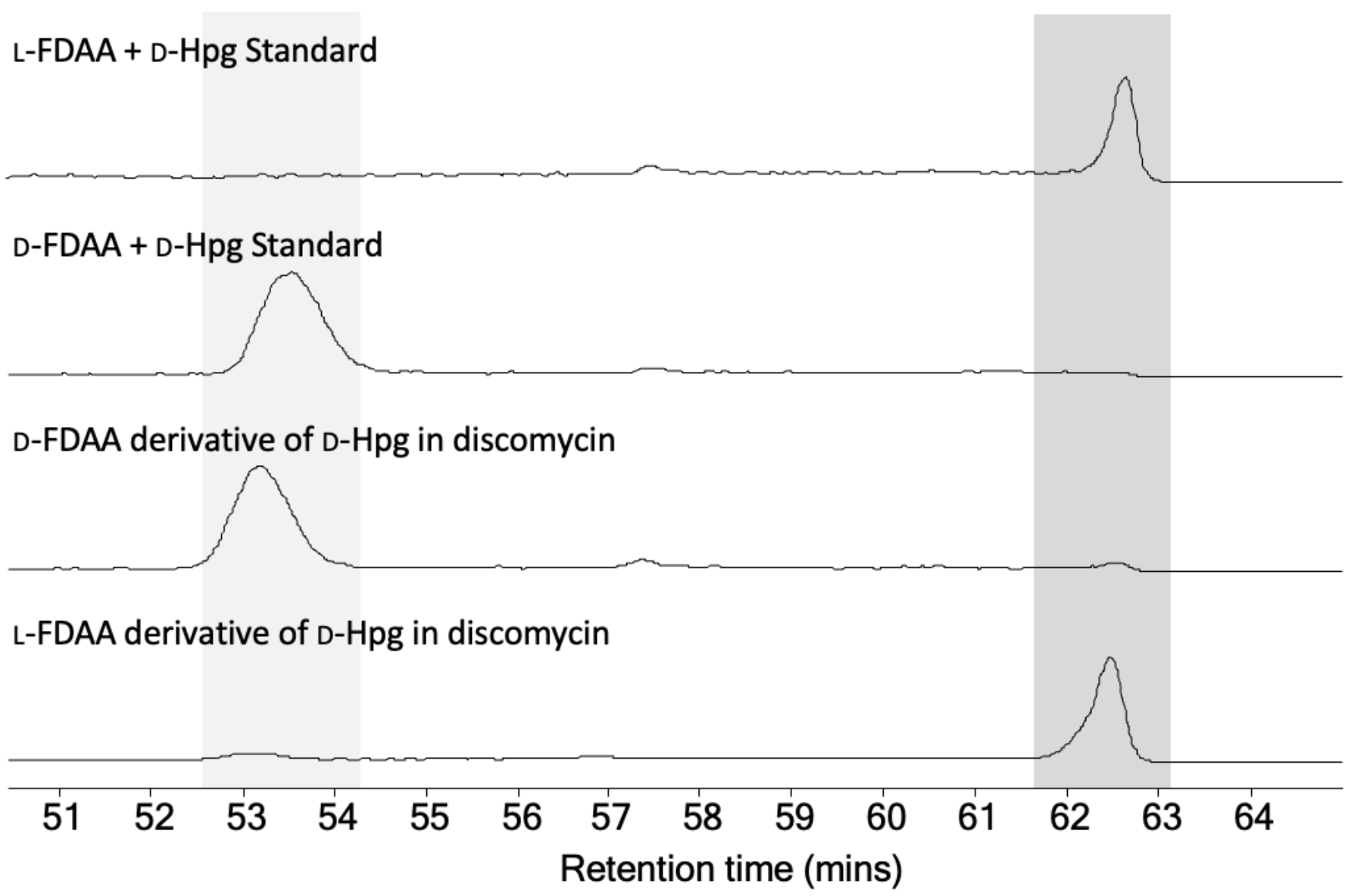

F) Gly, *m/z* = 327.5888 – 328.5888

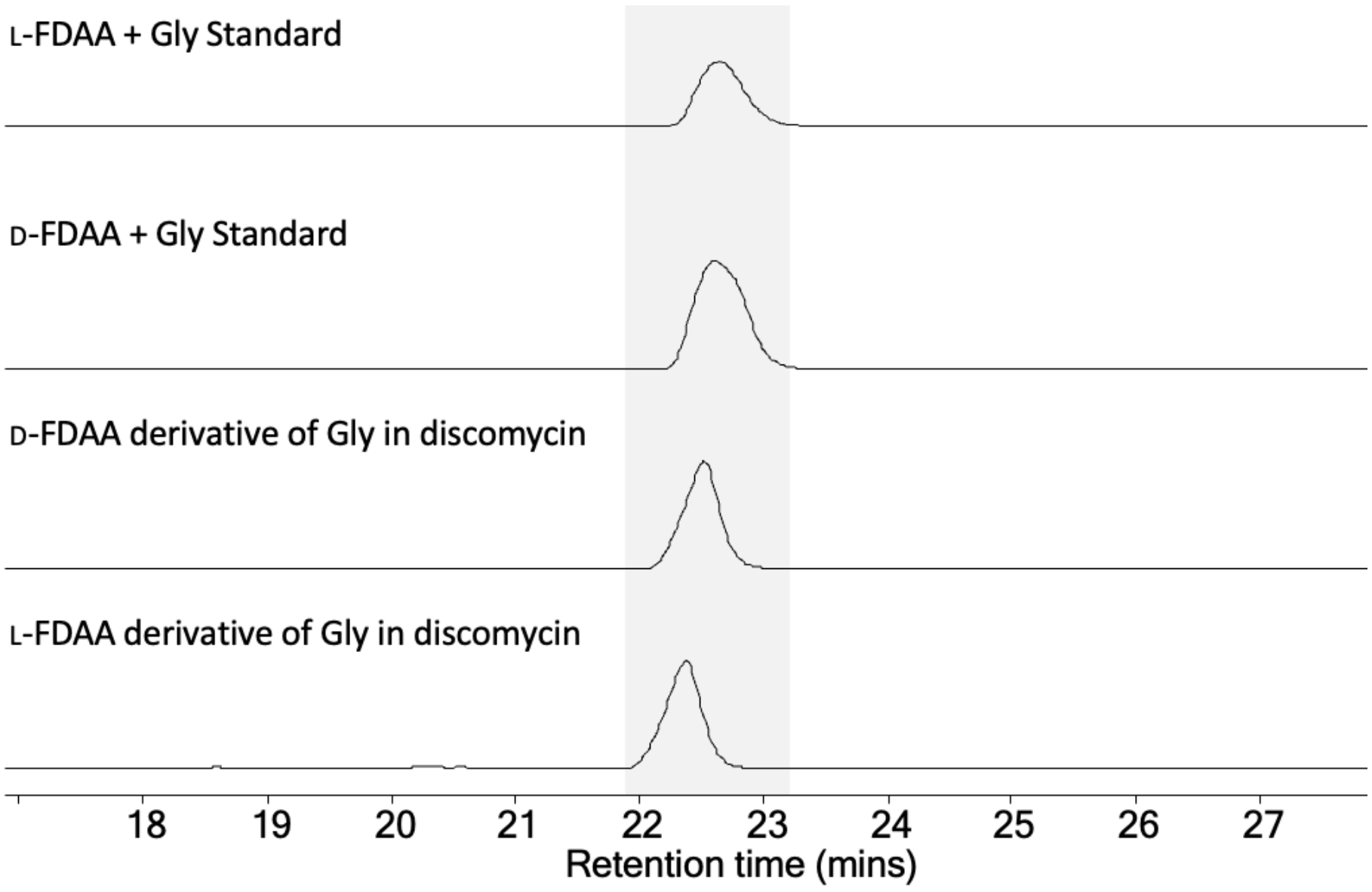

G) β Me-Glu, *m/z* = 413.6256 – 414.6256

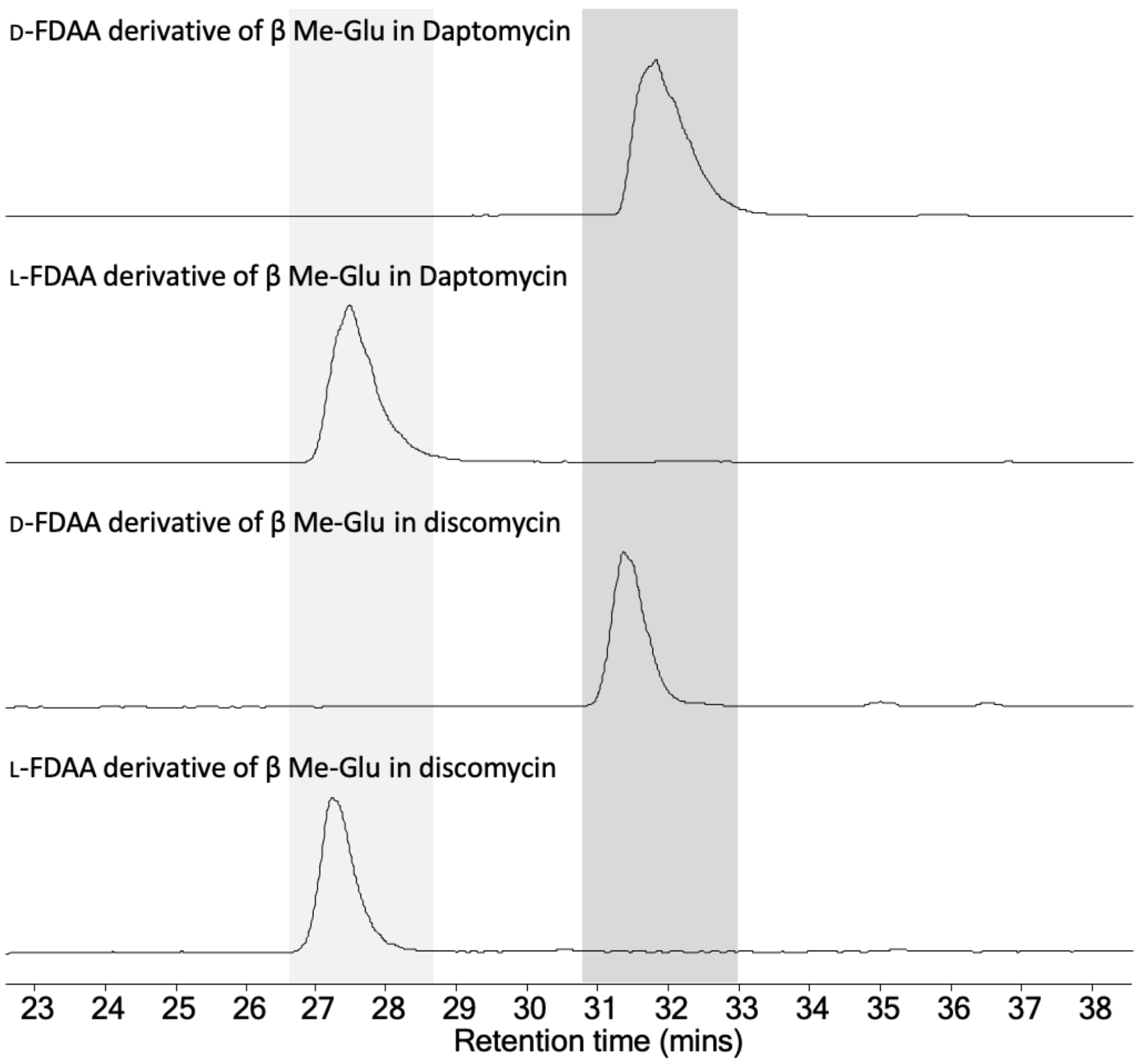

H) Ile, *m/z* = 383.6514 – 384.6514

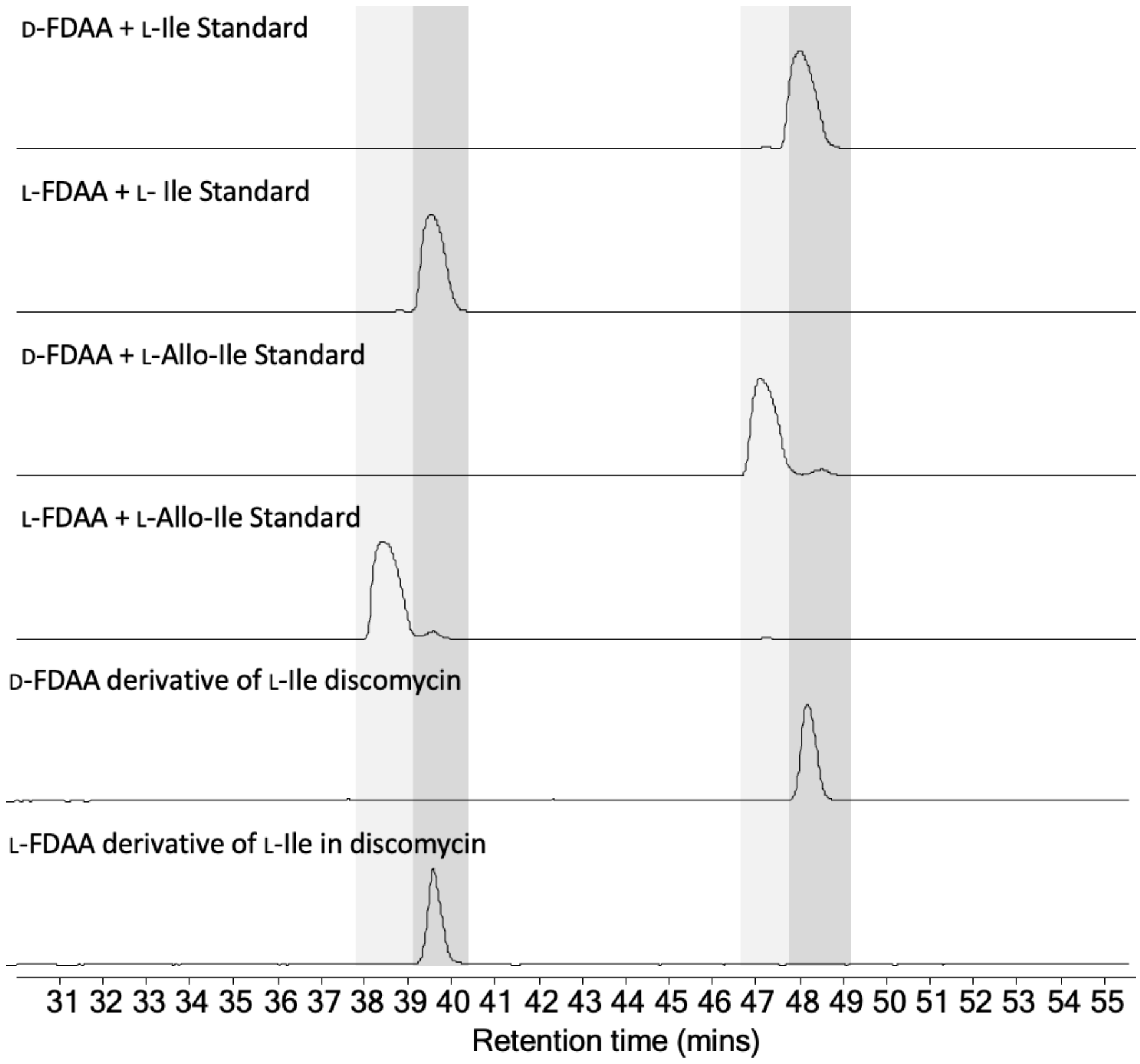

Supplementary Figure 8. 1H spectrum of discomycin A

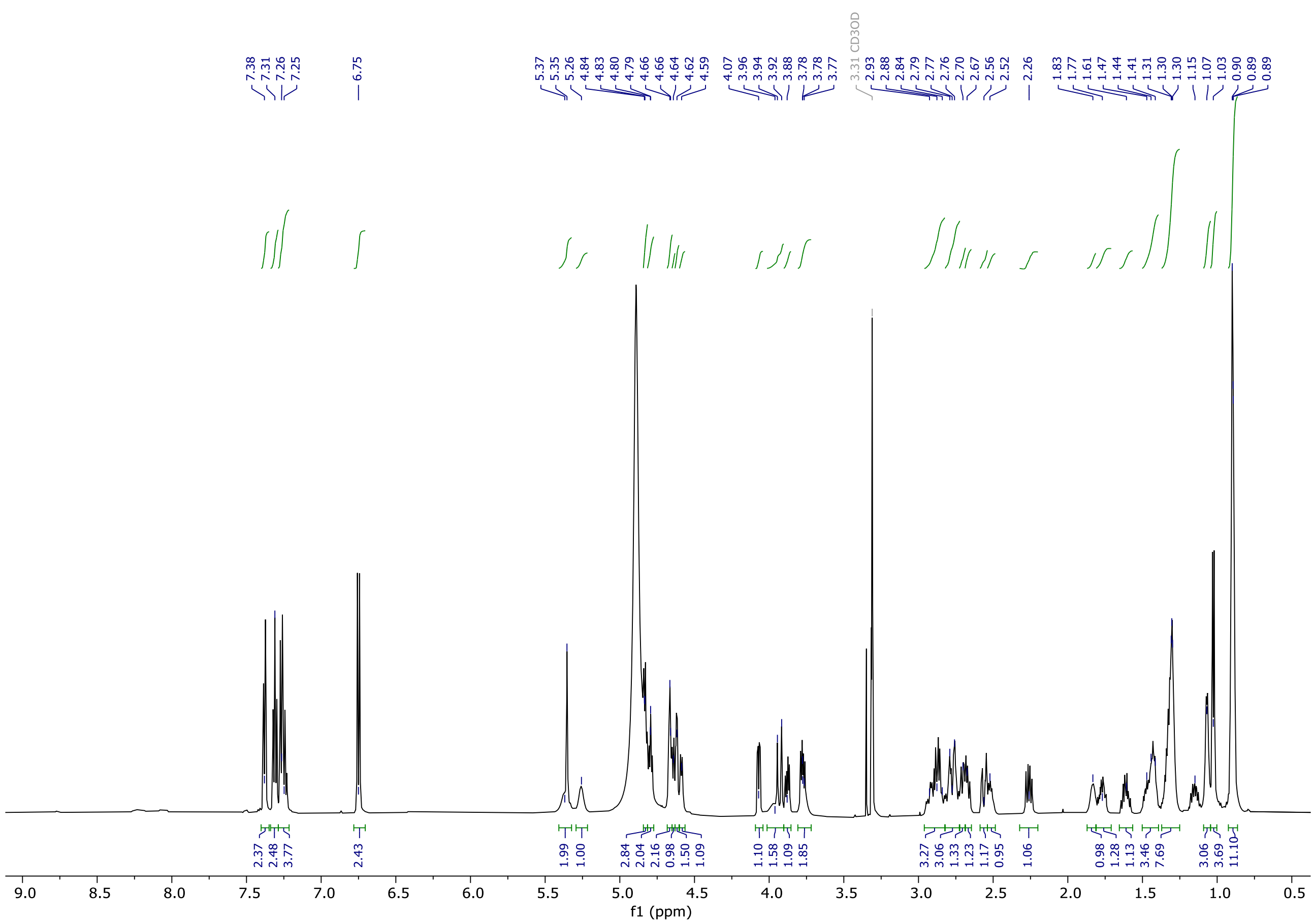

Supplementary Figure 9. 1H-1H COSY Spectrum of discomycin A

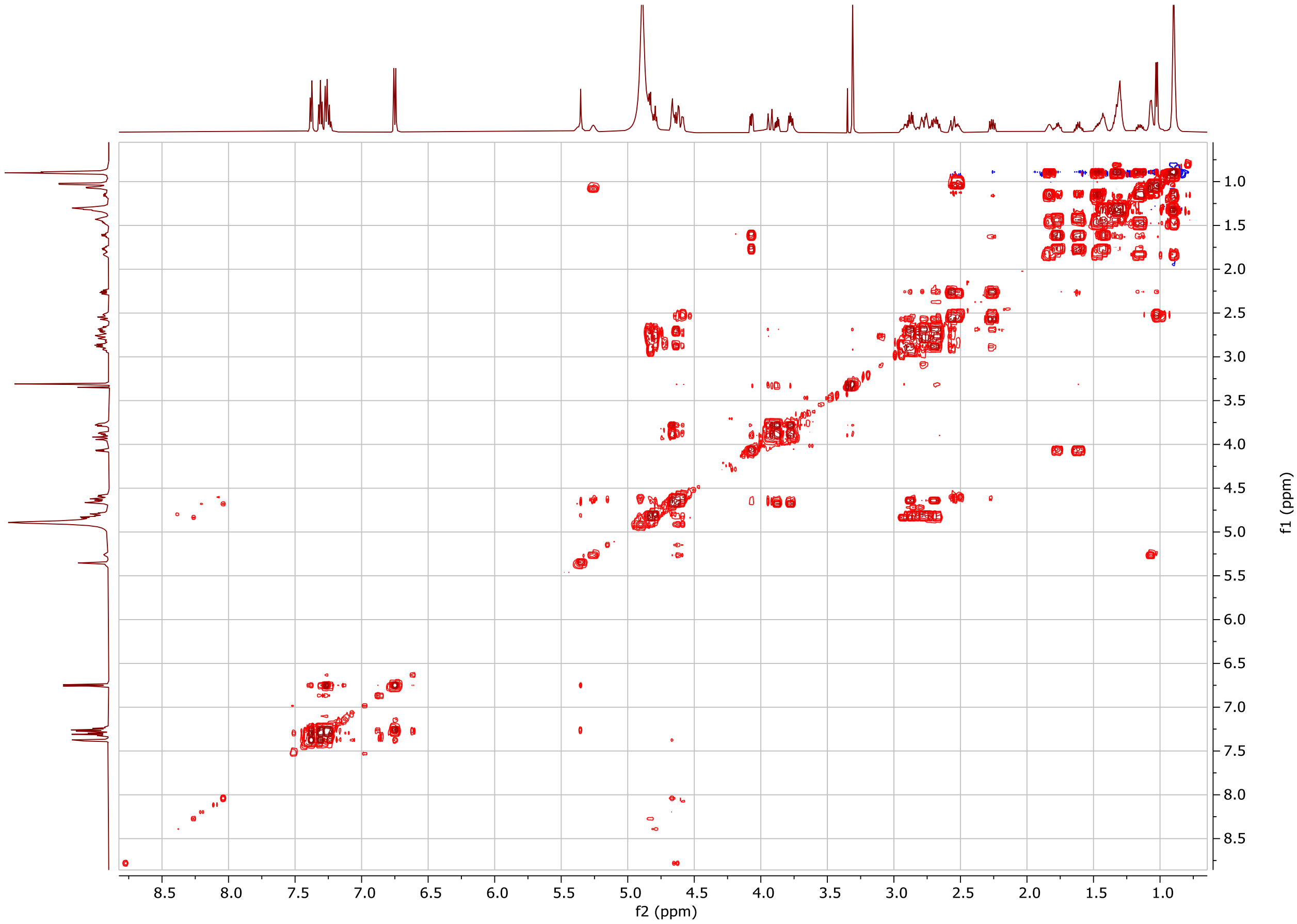

Supplementary Figure 10. 13C spectrum of discomycin A

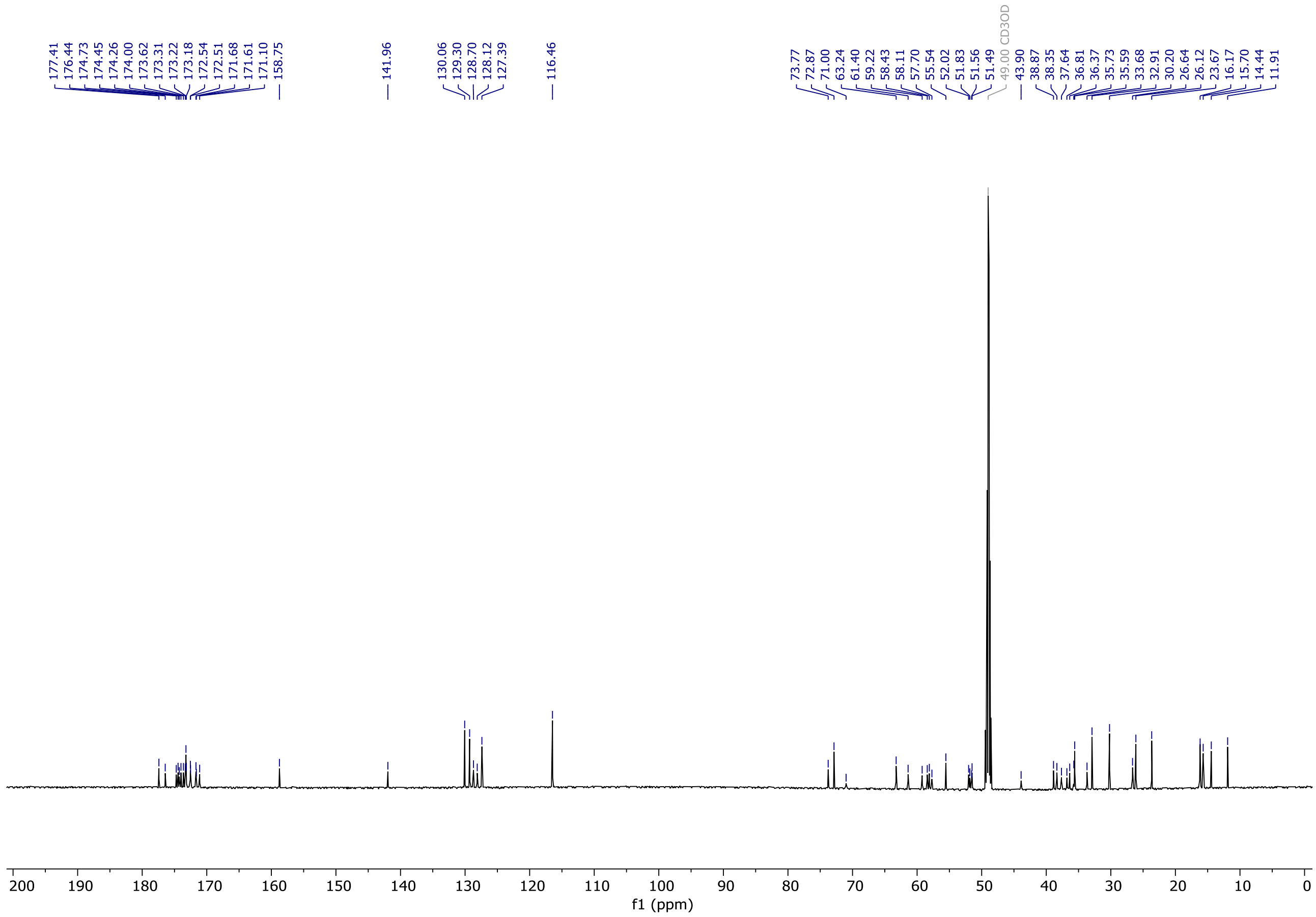

Supplementary Figure 11. 1H-13C HSQC spectrum of discomycin A

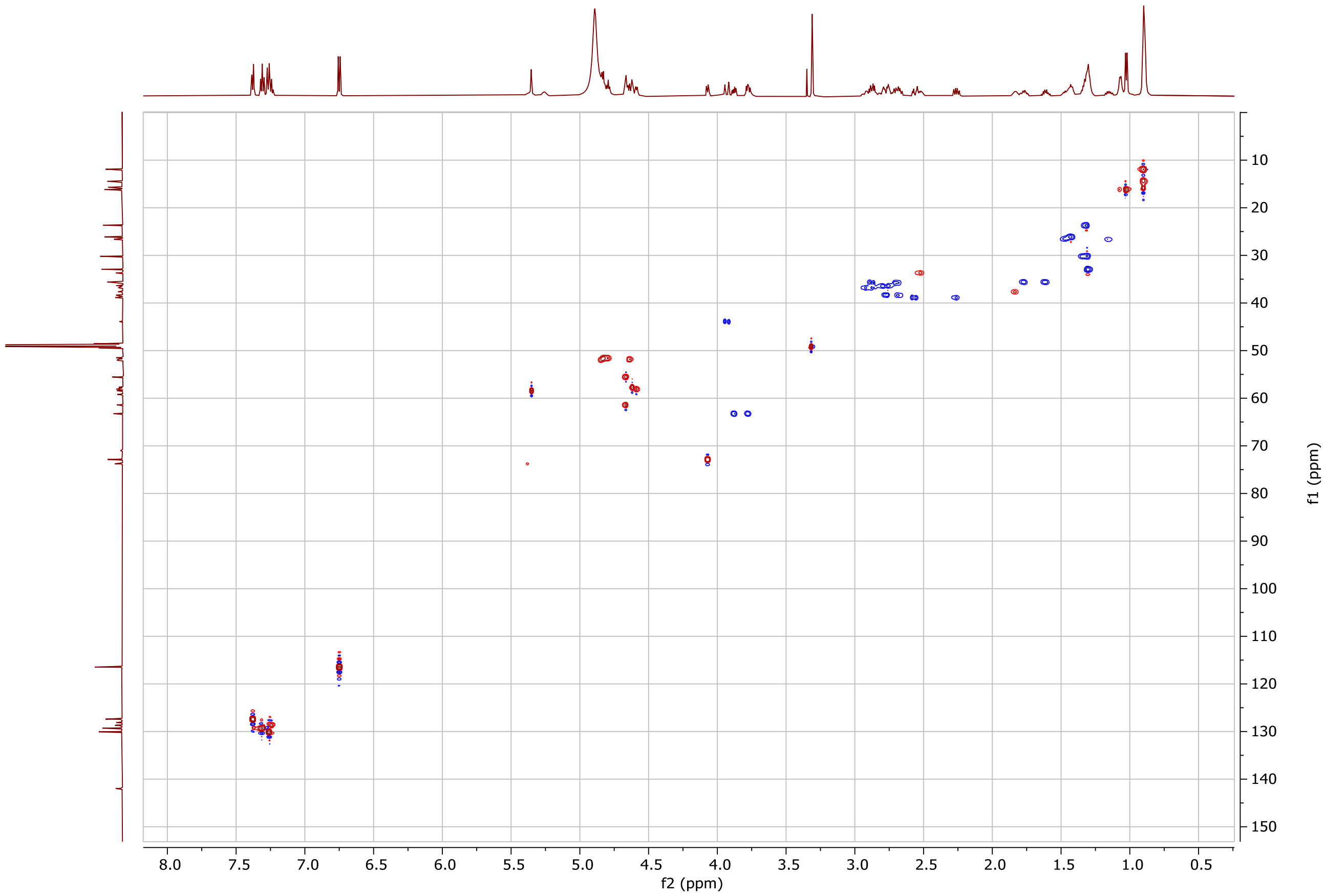

Supplementary Figure 12. 1H-1H ROESY spectrum of discomycin A

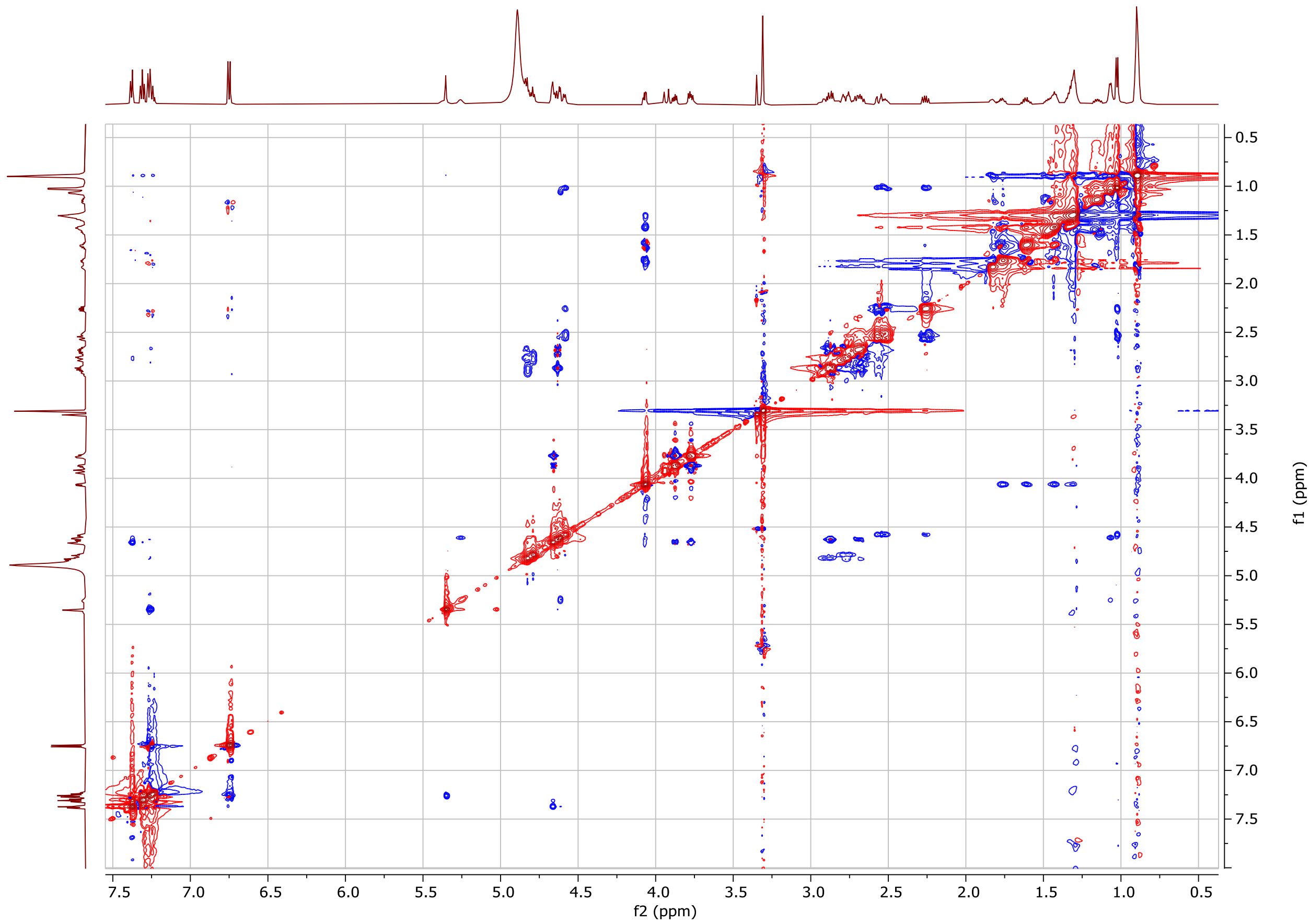

Supplementary Figure 13. 1H-13C HMBC spectrum of discomycin A

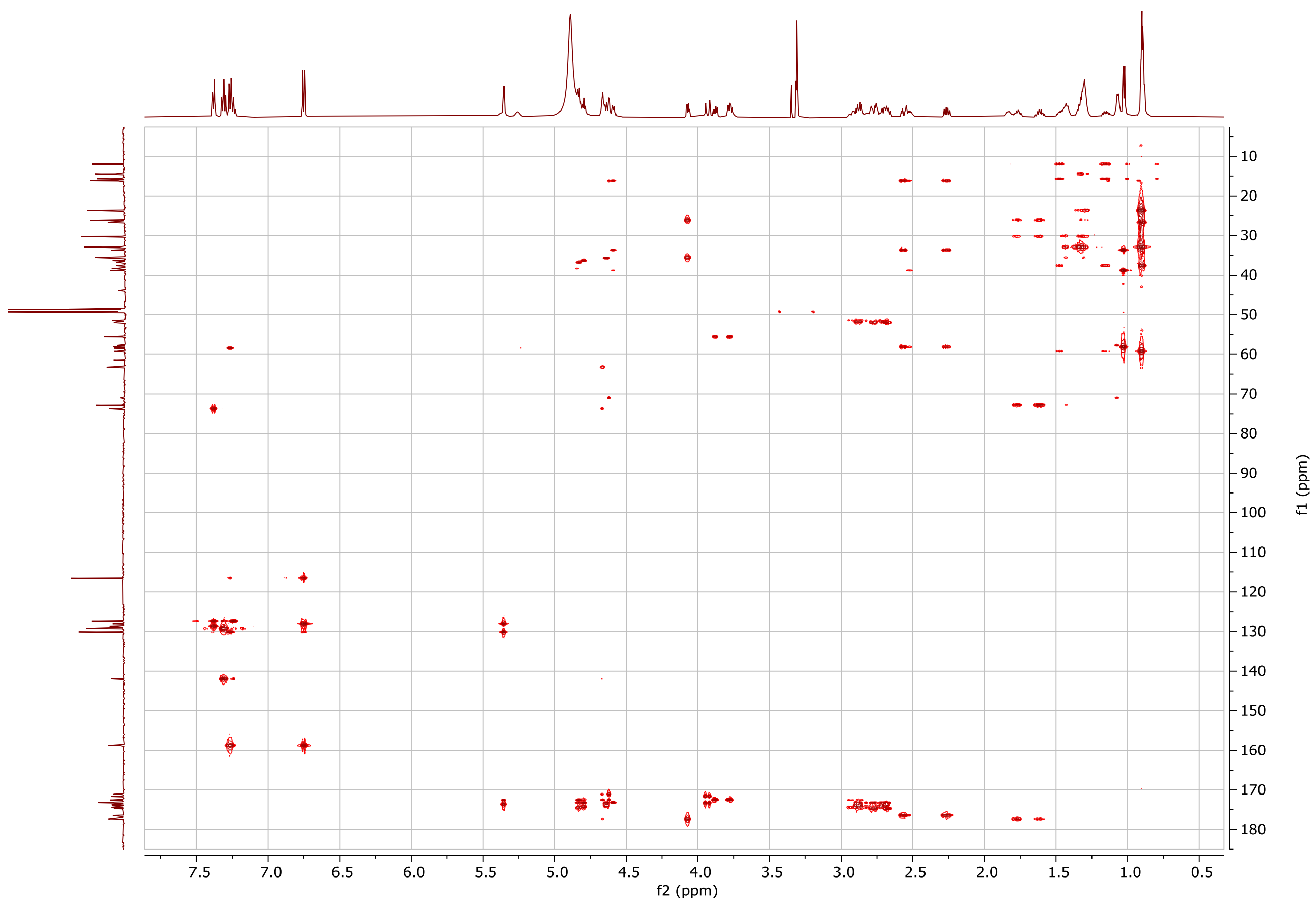

Supplementary Table 5. MIC values for discomycin A

Ca2+(mM)	B. subtilis	S. aureus	MRSA	HCT 116	E. coli	A. baumanii
**1**	1.0	>32	ND	ND	ND	ND
**2**	0.5	>32	ND	ND	ND	ND
**5**	0.125-0.25	32.0	ND	ND	ND	ND
**10**	0.125-0.25	8.0	ND	ND	ND	ND
**20**	0.1	2.0	4.0	>32.0	>32.0	>32.0
**50**	0.1	1.0	ND	ND	ND	ND
**100**	0.031-0.063	0.3	ND	ND	ND	ND

Presented data are range of values for biological. Assays were conducted in LB medium with the indicated concentration of $CaCl_2$

Supplementary Figure 14. DiscERN Output Dendrogram Showing Relatedness of Putative Hits

Hits supported by K≥3 algorithms are presented. With the exception of the rifamycin like dendrogram, only hits passing the automatic Pfam filter function of DiscERN are presented

A) Macrolides

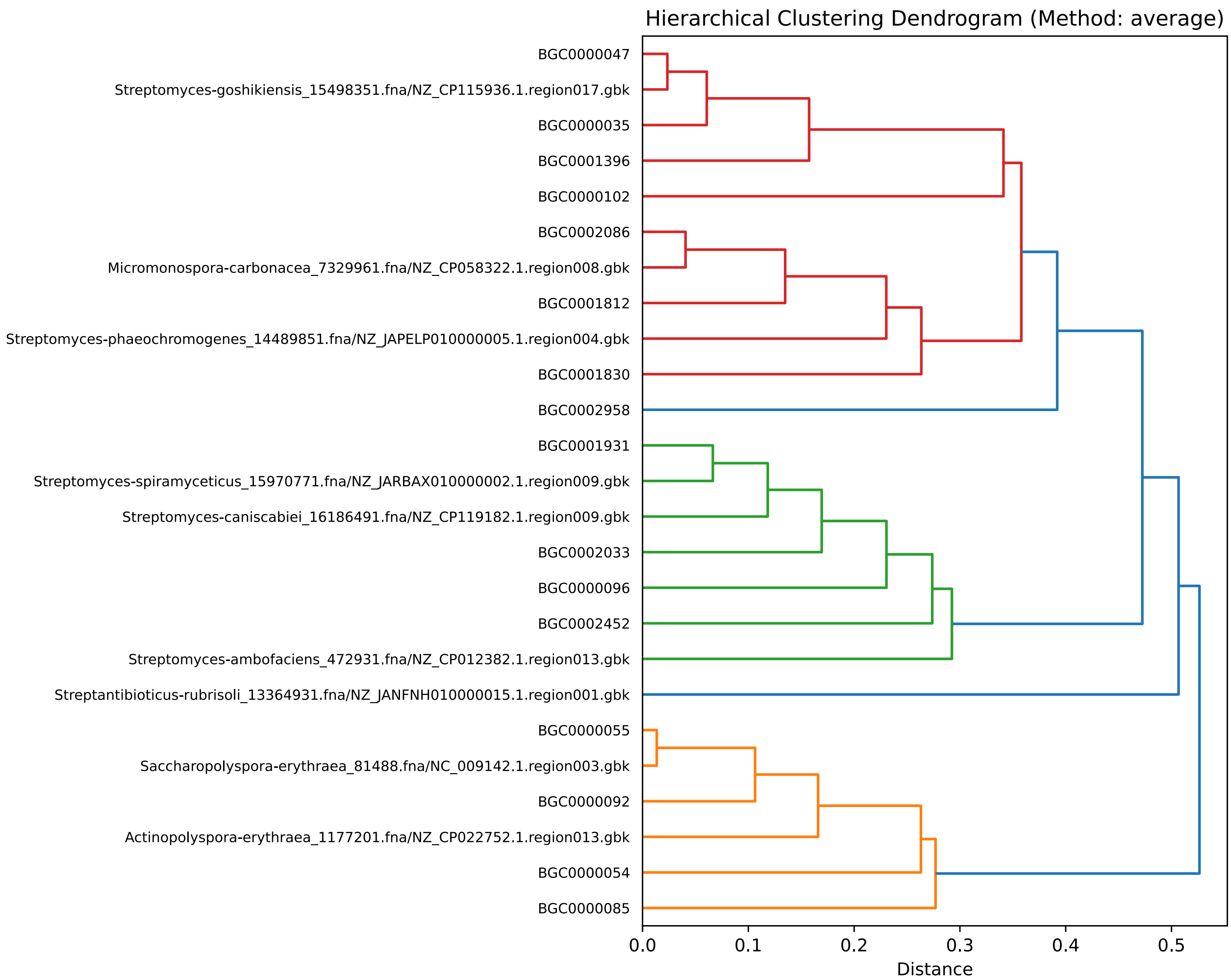

B) Glycopeptides

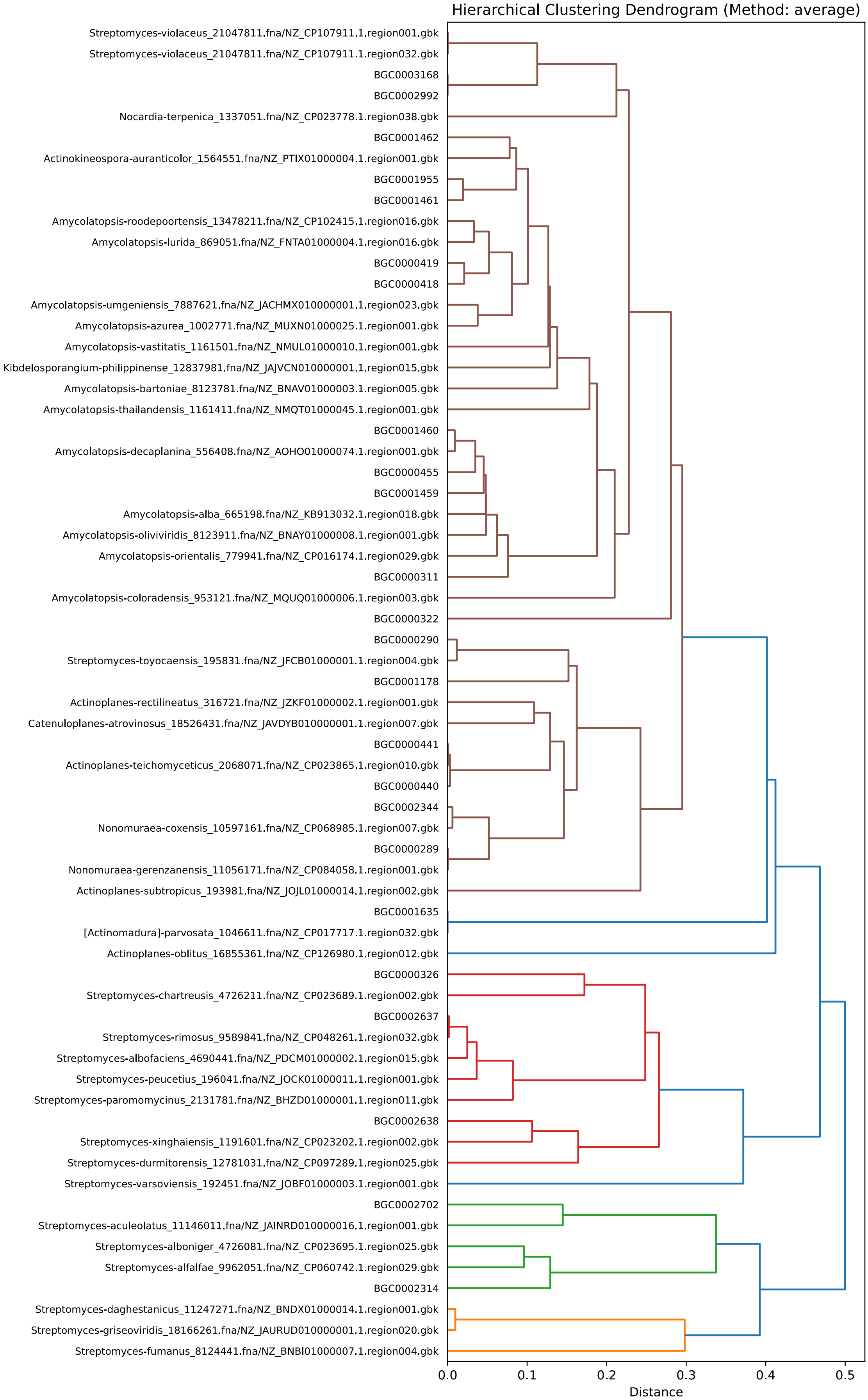

C) CDAs

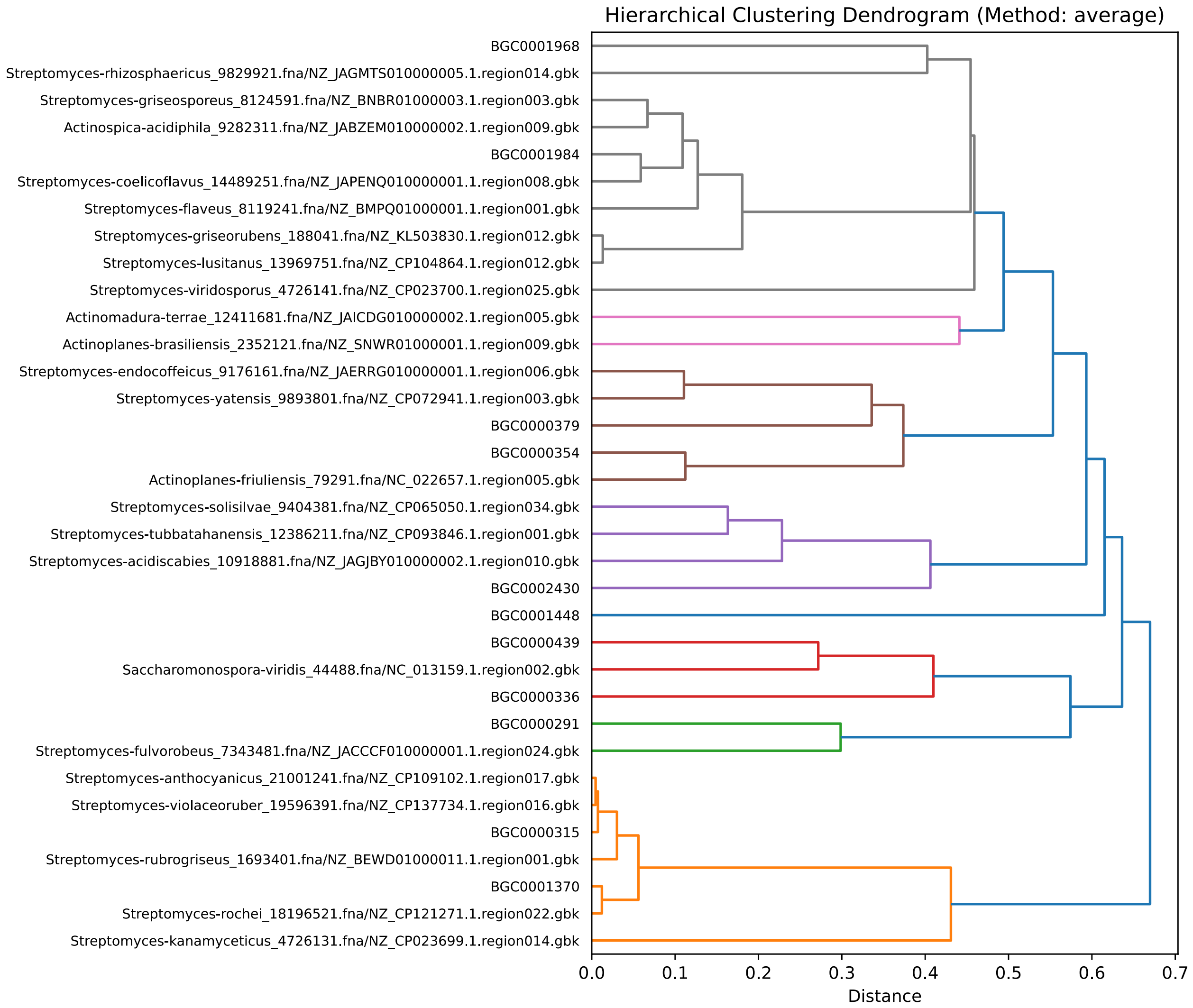
Hierarchical Clustering Dendrogram (Method: average)
BGC0001968
Streptomyces-rhizosphaericus_9829921.fna/NZ_JAGMTS010000005.1.region014.gbk
Streptomyces-griseosporeus_8124591.fna/NZ_BNBR01000003.1.region003.gbk
Actinospica-acidiphila_9282311.fna/NZ_JABZEM010000002.1.region009.gbk
BGC0001984
Streptomyces-coelicoflavus_14489251.fna/NZ_JAPENQ010000001.1.region008.gbk
Streptomyces-flaveus_8119241.fna/NZ_BMPQ01000001.1.region001.gbk
Streptomyces-griseorubens_188041.fna/NZ_KL503830.1.region012.gbk
Streptomyces-lusitanus_13969751.fna/NZ_CP104864.1.region012.gbk
Streptomyces-viridosporus_4726141.fna/NZ_CP023700.1.region025.gbk
Actinomadura-terrae_12411681.fna/NZ_JAICDG010000002.1.region005.gbk
Actinoplanes-brasiliensis_2352121.fna/NZ_SNWR01000001.1.region009.gbk
Streptomyces-endocoffeicus_9176161.fna/NZ_JAERRG010000001.1.region006.gbk
Streptomyces-yatensis_9893801.fna/NZ_CP072941.1.region003.gbk
BGC0000379
BGC0000354
Actinoplanes-friuliensis_79291.fna/NC_022657.1.region005.gbk
Streptomyces-solisilvae_9404381.fna/NZ_CP065050.1.region034.gbk
Streptomyces-tubbatahanensis_12386211.fna/NZ_CP093846.1.region001.gbk
Streptomyces-acidiscabies_10918881.fna/NZ_JAGJBY010000002.1.region010.gbk
BGC0002430
BGC0001448
BGC0000439
Saccharomonospora-viridis_44488.fna/NC_013159.1.region002.gbk
BGC0000336
BGC0000291
Streptomyces-fulvorobeus_7343481.fna/NZ_JACCCF010000001.1.region024.gbk
Streptomyces-anthocyanicus_21001241.fna/NZ_CP109102.1.region017.gbk
Streptomyces-violaceoruber_19596391.fna/NZ_CP137734.1.region016.gbk
BGC0000315
Streptomyces-rubrogriseus_1693401.fna/NZ_BEWD01000011.1.region001.gbk
BGC0001370
Streptomyces-rochei_18196521.fna/NZ_CP121271.1.region022.gbk
Streptomyces-kanamyceticus_4726131.fna/NZ_CP023699.1.region014.gbk
0.0
0.1
0.2
0.3
0.4
0.5
0.6
0.7
Distance

D) Rifamycin Like

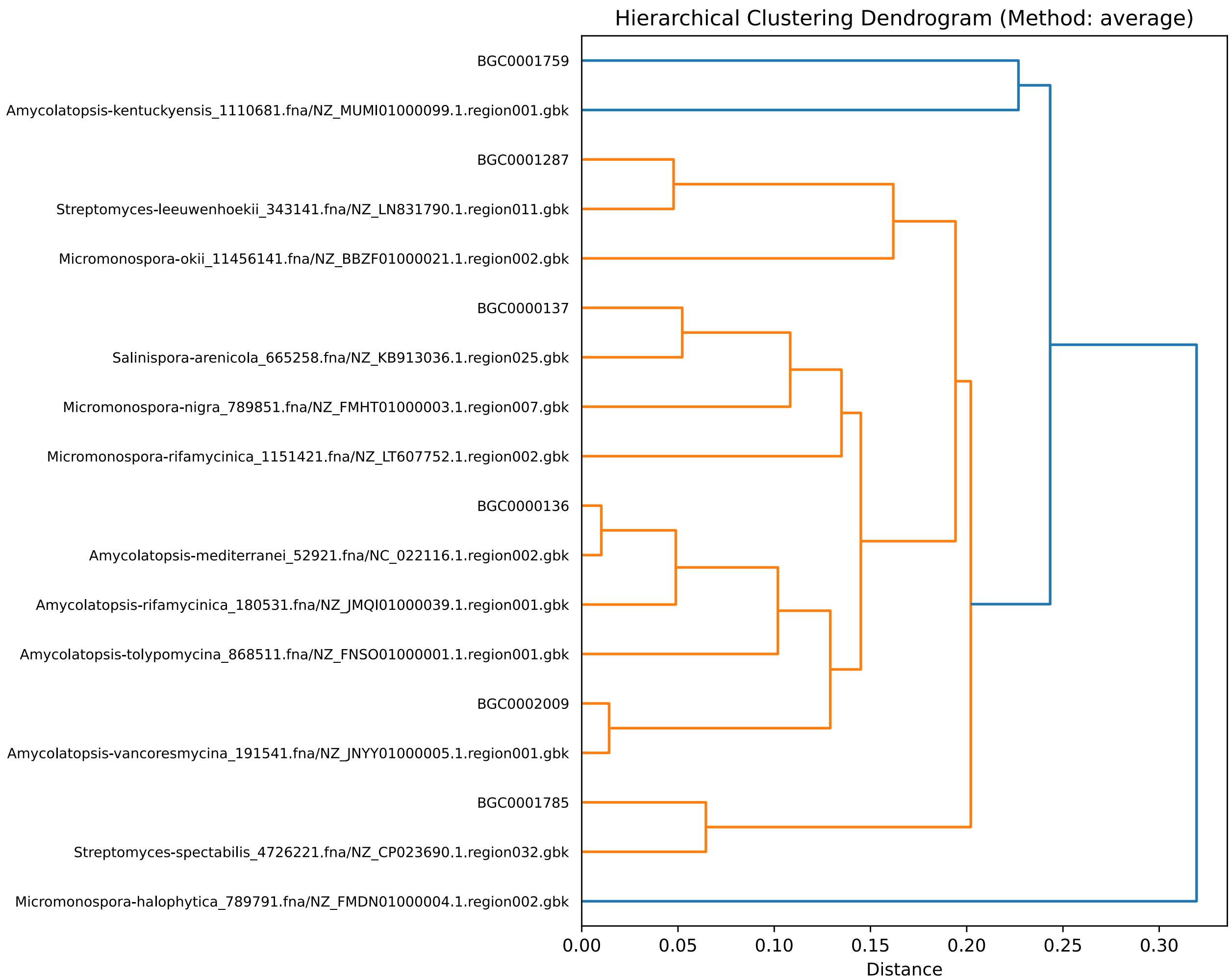

Supplementary Table 6. Classification Performance for BiG-SCAPE at Various Clustering Thresholds:

Cutoff	TP	FP	FN	Precision	Recall	F1 score
**0.3**	6	0	36.68	1.00	0.14	0.25
**0.35**	12	0	30.68	1.00	0.28	0.44
**0.4**	16	0	26.68	1.00	0.38	0.55
**0.45**	15	10	27.68	0.60	0.35	0.44
**0.5**	17	20	25.68	0.46	0.40	0.43
**0.55**	17	27	25.68	0.39	0.40	0.39
**0.6**	17	30	25.68	0.36	0.40	0.38

Table S6. Performance of BiG-SCAPE classification across clustering cutoffs (0.30–0.60), showing true positives (TP), false positives (FP), false negatives (FN), and derived precision, recall, and F1 score for each threshold. FN values were extrapolated using the theoretical positive value calculated from DiscERN results

Supplementary Table 7. Classification Performance for GATOR-GC at Various Gator Focal Score Thresholds:

Score	TP	FP	precision	recall_obs	recall_adj	f1_obs	f1_adj
1	1	0	1	0.043478	0.023429	0.08333	0.04578
0.98937	4	1	0.8	0.173913	0.093714	0.28571	0.16778
0.97329	6	6	0.5	0.26087	0.140571	0.34286	0.21945
0.94658	14	18	0.4375	0.608696	0.328	0.50909	0.37492
0.77897	15	18	0.454545	0.652174	0.351429	0.53571	0.39639
0.68251	19	18	0.513514	0.826087	0.445143	0.63333	0.47689
0.66709	19	19	0.5	0.826087	0.445143	0.62295	0.47098
0.65776	22	24	0.478261	0.956522	0.515429	0.63768	0.49615
0.63105	23	63	0.267442	1	0.538857	0.42202	0.35747

Table S7. Performance of GATOR-GC classification across Gator focal score thresholds, reporting true positives (TP), false positives (FP), precision, observed and adjusted recall (recall_obs, recall_adj), and corresponding observed and adjusted F1 scores (f1_obs, f1_adj). Adjusted values were extrapolated using the theoretical positive value calculated from DiscERN results. **Note**: The nine scores capture all observed gator focal scores from the 100 manually examined BGCs with all of the 100 BGCs being assigned one of these scores by GATOR-GC.

Supplementary Table 8. qPCR Ct values for all biological and technical replicates:

Biological replicate	Condition	Target (primers)	Ct (Tech 1)	Ct (Tech 2)	Ct (Tech 3)
1	EV	NRPS	33.93	33.34	33.17
1	EV	GyrA	23.93	22.27	22.9
1	SARP	NRPS	26.71	26.59	26.72
1	SARP	GyrA	23.52	23.53	23.38
2	EV	NRPS	34.7	35.21	34.97
2	EV	GyrA	24.56	24.5	24.42
2	SARP	NRPS	27.92	27.96	28.13
2	SARP	GyrA	24.91	24.03	24.05
3	EV	NRPS	34.21	34.95	34.38
3	EV	GyrA	23.59	23.52	23.41
3	SARP	NRPS	27.96	27.99	ND
3	SARP	GyrA	23.73	23.67	23.63

Table S8. Raw qPCR C_t values for the NRPS target and *gyrA* reference genes in EV and SARP strains across three independent biological replicates, each measured in technical triplicate; ND indicates no C_t value recorded.

Supplementary Figure 15. Comparison of new core sequences from thiostreoton-like RiPP BGCs identified by DiscERN to characterised thiostrepton-like core sequences

Upper box shows core sequences from known thiostrepton-like antibiotics. Lower box shows cores from BGCs identified by DiscERN that do not match any known sequences exactly. In addition to these divergent cores, eight BGCs identified by DiscERN had a core sequence identical to one or more of the currently known thiostrepton like antibiotics. Bolded residues indicate positions which are variable in known compounds. Red bolded positions indicate differences in the newly identified cores.

saalfelduracin:	**-S**SSSCTTCICTCSCSS
thiostrepton:	**IA**S**A**SCTTCICTCSCSS
Sch 18640:	**IA**S**A**SCTTCICTCSCSS
Sisomycin:	**VS**S**A**SCTTCICTCSCSS
Thiopeptin:	**VA**S**A**SCTTCICTCSCSS
NZ_DF968533_1:	**-S**S**V**SCTTCIC**S**CSCSS
NZ_JARJBC010000016:	**--**S**A**SCTTCICTCSCSS

Supplementary References:

1 Enghiad, B. *et al.* Cas12a-assisted precise targeted cloning using in vivo Cre-lox recombination. *Nat. Commun.* **12**, 1171 (2021).

2 Kieser, T., Bibb, M. J., Buttner, M. J., Chater, K. F. & Hopwood, D. A. *Practical Streptomyces Genetics*. (John Innes Foundation, 2000).

3 Vijayasarathy, S. *et al.* C3 and 2D C3 Marfey's Methods for Amino Acid Analysis in Natural Products. *J. Nat. Prod.* **79**, 421-427 (2016).
