# Supplementary material for: DiscERN: an automated genome mining tool for the discovery of evolutionarily related natural products
Source: Nat Commun. 2026 Jul 14;17:8657. doi: 10.1038/s41467-026-75491-x (PMC13490569; doi:10.1038/s41467-026-75491-x)
Supplement: Supplementary file 3 [file 41467_2026_75491_MOESM2_ESM.pdf]

Description of Additional Supplementary Files

Title: Supplementary Data 1.
Description: Excel workbook containing MIBiG accession numbers and BGC family assignments for all reference BGCs used in this study.

Title: Supplementary Data 2.
Description: Summary of manual analysis of BGCs for the four antibiotic families presented in figure 3. Numbering matches the antiSMASH outputs in Supplementary Data File 3.

Title: Supplementary Data 3.
Description: Zipped folder containing antiSMASH outputs for all putative hits from screen of 3,561 Actinomycetes genomes with four antibiotic families. https://zenodo.org/records/16791517

Title: Supplementary Data 4.
Description: Excel workbook containing accession numbers and species names for the genomes in the *Actinomycetes* dataset and *Streptomyces* subset.

Title: Supplementary Data 5.
Description: Excel workbook containing manual classification assignments for BiG-SCAPE CDA hits. Numbering matches the antiSMASH outputs in Supplementary Data File 10.

Title: Supplementary Data 6.
Description: Excel workbook containing manual classification assignments for GATOR-GC CDA hits. Numbering matches the antiSMASH outputs in Supplementary Data File 11.

Title: Supplementary Data 7.
Description: Zipped folder containing BiG-SCAPE and GATOR-GC output files from benchmarking experiments.

Title: Supplementary Data 8.
Description: Jupyter notebook containing output parsing and analysis code used for benchmarking experiments. Available at: https://zenodo.org/records/18667964

Title: Supplementary Data 9.
Description: Zipped folder containing antiSMASH outputs from analysis of thiostrepton-like antibiotic BGCs identified by DiscERN. Available at: https://zenodo.org/records/18667964

Title: Supplementary Data 10.
Description: Zipped folder containing antiSMASH outputs for all putative hits identified by BiG-SCAPE in benchmarking experiments. Available at: https://zenodo.org/records/18667964

Title: Supplementary Data 11.
Description: Zipped folder containing antiSMASH outputs for all putative hits identified by GATOR-GC in benchmarking experiments. Available at: https://zenodo.org/records/18667964
