# Supplementary material for: DiscERN: an automated genome mining tool for the discovery of evolutionarily related natural products
Source: Nat Commun. 2026 Jul 14;17:8657. doi: 10.1038/s41467-026-75491-x (PMC13490569; doi:10.1038/s41467-026-75491-x)
Supplement: Supplementary file 4 [file 41467_2026_75491_MOESM4_ESM.pdf]

	Modules	Closest	Hit	Reference
2	New	Rosamicin		
3	New	Rosamicin		
4	N/D, Partial	Spiramycin		
5	N/D, Partial	Rosamicin		
6	N/D, Partial	Mycinamicin		
7	False Positive	Carrimycin		
8	N/D, Partial	Leucomycin		
9	N/D, Partial	Chalcomycin		
	**Macrolides Unfiltered K1 (0/2 TP + 5 N/D Partial)**			
1	N/D, Partial	Rosamicin		
2	N/D, Partial	Lankamycin		
3	N/D, Partial	Ibomycin		
4	N/D, Partial	Carrimycin		
5	False Positive	Polyoxypeptin		
6	False Positive	Efomycin		
7	N/D	Dehydro-chalcomycin		
	**Rifamycin Filtered K3 (1/1 TP)**			
1	Identical	Chaxamycin		
	**Rifamycin Filtered K1 (1/1 TP)**			
1	Identical	Kanglemycin		
	**Rifamycin Unfiltered K4 (9/9 TP)**			
1	Identical, partial	Rifamycin		
2	Identical	Kanglemycin		
3	Identical	Rifamycin		
4	Identical	Kanglemycin		
5	Identical*	Kanglemycin		
6	V. Similar	Rifamycin		
7	Identical	Rifamycin		
8	New	Rifamycin		
9	Identical	Rifamycin		
	**Rifamycin Unfiltered K3 (2/2 TP)**			
1	Identical	Streptovaricin		
2	Identical, partial	Rifamycin		
	**Rifamycin Unfiltered K2 (3/3 TP)**			
1	Identical, partial	Rifamycin		
2	N/D, Partial	Streptovaricin		
3	Identical, partial	Rifamycin		
4	Identical, partial	Chaxamycin		
	**Rifamycin Unfiltered K1 (3/11 TP + 13 N/D Partial)**			
1	False Positive	N/A		
2	False Positive	Hygrocin		
3	Identical, partial	Streptovaricin		
4	N/D, Partial	N/A		
5	False Positive	Rubradirin		
6	N/D, Partial	Chaxamycin		
7	False Positive	Rubradirin		
8	False Positive	Microansamycin		
9	False Positive	N/A		
10	N/D, Partial	Napthomycin		
11	N/D, Partial	N/A		
12	N/D, Partial	Rifamycin		
13	N/D, Partial	N/A		
14	N/D, Partial	N/A		
15	N/D, Partial	N/A		
16	False Positive	rifamycin		
17	N/D, Partial	N/A		
18	False Positive	Kanglemycin		
19	N/D, Partial	N/A		
20	Identical, partial	Rifamycin		
21	N/D, Partial	N/A		
22	Identical, partial	Rifamycin		
23	N/D, Partial	N/A		
24	N/D, Partial	N/A		
	**Glycopeptides Filtered K4 (20/36 analysed, 20/20 TP)**			
1	Identical	Rimomycin		
2	New	Ristomycin		
3	Identical	Ristomycin		
4	Identical	UK-68,597		
5	Identical	Ristomycin		
6	Identical	Keratinimicin		
7	Identical	Decaplanin		
8	Identical	Rimomycin		
9	Identical	Teicoplanin		
10	Identical	misaugamycin		
11	Identical	Rimomycin		
12	Identical	Teicoplanin		
13	Identical	A40926		
14	Identical	Kistamycin		
15	Identical	Ristomycin		
16	Identical	Ristomycin		
17	Identical	Rimomycin		
18	Identical	A50926		
19	Identical	Avoparcin		
20	Identical	Decaplanin		
	**Glycopeptides filtered K3 (6/6 TP)**			
1	New	Cobromycin		
2	New	Cobromycin		
3	Identical	Cobromycin		
4	New	Cobromycin		
5	Identical	Cobromycin		
6	Identical	UK-68,597		
	**Glycopeptides filtered K2 (1/1 TP)**			
	New	Cobromycin		
	**Glycopeptides unfiltered K4 (3/3 TP)**			
1	Identical, partial	misaugamycin		
2	Identical	rimomycin		
3	Identical, partial	Kistamycin		
4	New, partial, fragmented	Cobromycin		
	**Glycopeptides unfiltered K3 (7/7 TP + 5 N/D Partial)**			
1	New, partial	Cobromycin		
2	Identical, partial	Kistamycin		
3	Identical, partial	rimomycin		
4	N/D, Partial	N/A		
5	N/D, Partial	N/A		
6	New	Cobromycin		
7	N/D, Partial	N/A		
8	Identical, partial	Cobromycin		
9	N/D, Partial	N/A		
10	New	Cobromycin		
11	New	Cobromycin		
12	N/D, Partial	N/A		
	**Glycopeptides unfiltered K2 (20/53 analysed, 2/5 TP + 15 N/D Partial)**			
1	N/D, Partial	N/A		
2	N/D, Partial	N/A		
3	Identical, partial	Cobromycin		
	N/D, Partial	N/A		
4	False Positive	Cobromycin		
5	N/D, Partial	N/A		
6	N/D, Partial	N/A		
7	N/D, Partial	N/A		
8	N/D, Partial	N/A		
9	N/D, Partial	N/A		
10	False Positive	N/A		
11	N/D, Partial	N/A		
12	N/D, Partial	N/A		
13	N/D, Partial	N/A		
14	N/D, Partial	N/A		
15	New, partial	Cobromycin		
16	N/D, Partial	N/A		
17	N/D, Partial	N/A		
18	N/D, Partial	N/A		
19	False Positive	feglymycin		
20	N/D, Partial	N/A		
	**Glycopeptides unfiltered K1 (20/198 analysed, 0/16 TP + 4 N/D Partial)**			
1	False Positive	N/A		
2	N/D, Partial	N/A		
3	False Positive	N/A		
4	False Positive	N/A		
5	False Positive	N/A		
6	N/D, Partial	N/A		
7	N/D, Partial	N/A		
8	False Positive	N/A		
9	False Positive	N/A		
10	False Positive	N/A		
11	False Positive	N/A		
12	False Positive			
13	N/D, Partial	N/A		
14	False Positive	coprisamide		
15	False Positive	N/A		
16	False Positive	N/A		
17	False Positive	N/A		
18	False Positive	feglymycin		
19	False Positive	feglymycin		
20	False Positive	Kistamycin		
